# Eating while distracted: a systematic review and meta-analysis of the effect of distraction on concurrent and later energy intake in adults

**DOI:** 10.1016/j.ajcnut.2026.101315

**Published:** 2026-04-16

**Authors:** Thomas Gough, Traci Mann, Khaleda Ahmadyar, Isla Finlay, Andrew Jones, Katy Tapper, Eric Robinson

**Affiliations:** 1 Department of Psychology, University of Liverpool, Liverpool, United Kingdom; 2 Department of Psychology, University of Minnesota Twin Cities, Minneapolis, MN, United States; 3 Department of Psychology, City St George’s, University of London, London, United Kingdom; 4 College of Health and Life Sciences, Aston University, Birmingham, United Kingdom; 5 School of Psychology, Liverpool John Moores University, Liverpool, United Kingdom

**Keywords:** distraction, food intake, appetite, attention, obesity

## Abstract

**Background:**

Eating while distracted [e.g., television (TV) watching, phone use] is believed to increase food intake. A previous small meta-analysis of experimental studies (published in *American Journal of Clinical Nutrition*) supported this. Many studies have since been published, but there has been no updated analysis.

**Objectives:**

This study aimed to conduct an updated systematic review and meta-analysis to examine the effect of distraction on concurrent and later energy intake.

**Methods:**

Eligible articles (searching up to December 2024) were identified from: a previously conducted meta-analysis which included studies up until 2012; database searches from 2012 to 2024 (PsycINFO, Medline, and PubMed); and both forward and backward citation searching. We followed PRISMA guidelines and conducted generic variance inverse meta-analyses with intake as the outcome variable for both concurrent and later energy intake.

**Results:**

A total of 50 eligible studies were included (40 measuring concurrent intake, 10 measuring later intake). Random effects meta-analyses revealed that the overall effect of distraction on concurrent energy intake was nonsignificant [standardized mean difference (SMD) = 0.123, 95% confidence interval (CI): <–0.01, 0.25; *P* = 0.051]. Moderator analyses revealed that type of distractor moderated the effect of distraction on eating, with passive distractor tasks (e.g., TV watching) resulting in greater energy intake when distracted [SMD = 0.272 (95% CI: 0.128, 0.417)], whereas physically demanding distractors [SMD = –0.139 (95% CI: –0.334, 0.057)] and cognitively demanding distractors [SMD = 0.202 (95% CI: –0.028, 0.432)] did not. The effect of distraction on later energy intake was statistically significant, such that eating while distracted led to greater intake at a subsequent eating episode [SMD = 0.419 (95% CI: 0.195, 0.642)].

**Conclusions:**

Distracted eating increases later energy intake; however, the effect of distracted eating on concurrent energy intake is less consistent, and only relatively passive distractors may increase energy intake. Collectively, these findings suggest that distraction is a potential contributor to overeating.

This systematic review and meta-analysis was registered at PROSPERO as CRD42024518245.

## Introduction

Eating in a distracted state, whereby attentional resources are placed away from the act of eating, is thought to increase energy intake in adults [1]. Previous research has examined the effect of eating in a distracted state on energy consumed while under distraction (concurrent energy intake) and the effect that eating in this state has on subsequent energy intake (later energy intake). Boon et al. [2] investigated the effect of distraction (consisting of listening to a radio conversation) on energy intake in a laboratory study. Since then, several studies have been conducted and in 2013, a meta-analysis of laboratory experiments found that eating under distraction (compared with control) was associated with increases to both concurrent [number of studies = 10; standardized mean difference (SMD) = 0.39] and later energy intake (number of studies = 4; SMD = 0.76) in adults [1]. Distracted eating may produce an increase in energy intake because under distraction, attention shifts away from: the taste properties of energy being consumed [3,4]; cognitive goals relating to diet [5], and feelings of satiety [6]. Avoiding distraction while eating now forms dietary guidance provided to the general public [7].

Since the 2013 meta-analysis [1], a relatively large number of relevant studies have been published. Therefore, an updated systematic review is necessary to improve the precision of the overall effect size of distraction on food intake [8]. Furthermore, recent studies have suggested that eating under distraction may not increase concurrent energy intake [9]. Reasons for the disparity in findings across studies remain unclear; however, this may be partly explained by the different types of distractor tasks used across studies, as these vary widely, specifically in their attentional demand. Previous researchers have suggested that activities which require attentional demand to the point whereby an individual is distracted away from the act of eating may not produce increased concurrent intake [6,9], particularly activity that requires motor function, such as a driving simulator [6]. Therefore, divergent findings may be explained by the level of attention required during distracting activities. A distraction that demands a level of active engagement (e.g., motor function) may have no effects on concurrent energy intake, whereas passive distractions [e.g., television (TV) viewing] may increase intake. Similarly, studies have also varied in terms of the attentional demand of control conditions. Studies which use a control condition that is not free of distraction (such as memorizing a number [9]) may show a smaller difference in energy intake between conditions, compared with studies that use a distraction-free control, due to a smaller between-condition difference in attentional demand. The role of attentional demand may also extend to the presentation of food under distraction, whereby food presentation which requires individuals to attend to it to eat (e.g., choosing between multiple foods, requiring the use of cutlery) may negate any effect of experimentally manipulated distraction, compared with food presented in a way that can be eaten relatively “mindlessly” (e.g., eating out of a bowl of popcorn).

Individual differences may also moderate the effect of distraction on concurrent energy intake. Because eating under distraction has been hypothesized to divert attention away from cognitive goals. An individual’s level of dietary restraint may moderate the effect of distraction on energy intake, as distracted eating may temporarily suspend self-regulatory processes relating to dieting intentions [5,10]. Of note, the way in which dietary restraint is measured may affect this moderation, as some measures of dietary restraint (such as the original restraint scale) are believed to capture patterns of “unsuccessful” dieting—reflecting weight fluctuation and elements of disinhibited eating [[11], [12], [13]], whereas other measures [such as the three-factor eating questionnaire (TFEQ) and the Dutch Eating Behavior Questionnaire (DEBQ)] characterize dietary restraint as being associated with a lower likelihood of displaying disinhibited eating patterns [14].

The effect of distraction on later energy intake has also been previously reviewed with findings demonstrating that consuming a fixed meal under distraction leads to greater energy intake at a subsequent eating episode. This is thought to occur because eating under distraction may disrupt meal memory and lead to greater subsequent energy intake [15]. Extending these findings, the intermeal interval length between a fixed meal (consumed under distraction) and a later eating episode may influence the effect of distraction on later energy intake. This is because models of appetite regulation argue that the volume of food in the stomach partly determines short-term satiety through distention receptors within the stomach wall [16]. However, it is believed that this signal only occurs in the short term and is limited to meal cessation [17]. Furthermore, psychological factors (e.g., episodic memory of a meal) are believed to determine energy intake and appetite after a delayed period after consuming a meal [18]. In the context of eating while distracted, this theoretical position suggests that the effect of distraction on energy intake may be smaller when subsequent energy intake is measured shortly after distracted consumption, compared with a longer interval.

The current review and meta-analysis aimed to pool all available current evidence to better understand the effect of distraction on concurrent and later intake. In addition, we explored whether theoretically informed moderators may help to explain mixed findings to date. We predicted that distraction would lead to greater later intake and that longer intermeal intervals may be associated with larger effects. We did not make an overall prediction as to whether distraction would affect concurrent intake (due to inconsistent findings), but predicted that study factors (type of distractor, attentional load of control condition, and food presentation) and the individual-level factor of dietary restraint may act as moderators of the effect.

## Methods

**FIGURE 1:**
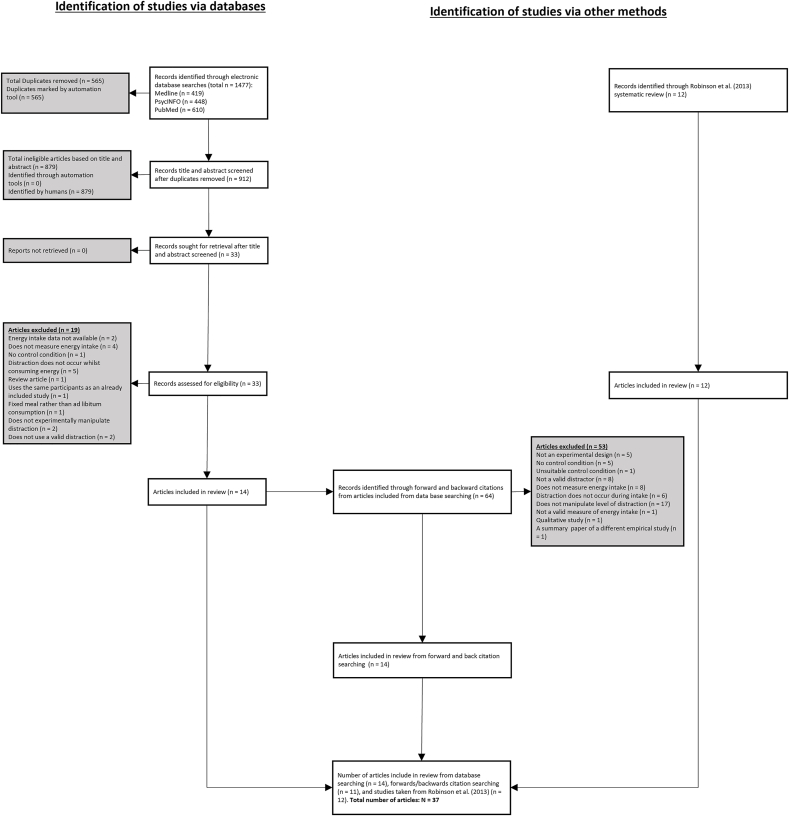
PRISMA flow diagram for study selection.

PRISMA guidelines were followed for this systematic review and meta-analysis [19]; see Figure 1 for PRISMA flowchart. The review was registered on PROSPERO (CRD42024518245) and a detailed analysis protocol was preregistered on the Open Science Framework https://osf.io/trp7x/.

### Eligibility criteria

#### Participants and intervention

Studies with neurologically intact human adults (≥18 y of age) were included. Studies examining the effect of distraction on either concurrent intake (distractor task presented while consuming food ad libitum) or later intake (food consumed ad libitum, after consuming a fixed meal which was consumed under distraction) were eligible for inclusion. Examples of eligible distractors were: TV viewing, playing computer games, listening to music/radio, reading, completing a computerized task which manipulates cognitive and/or attentional load (e.g., N-back task [20], Rapid Visual Information Processing task [21]). For concurrent intake studies only, we did not include the presence of or interaction with other people as a distractor, due to the confounding effect of eating with another person which is known to affect intake [22]. For concurrent intake studies, distractors that were specifically designed to contain food-related content were ineligible (e.g., TV viewing with food advertisements) due to the possibility that this type of food exposure could affect intake independent to distraction.

#### Comparator

Control conditions were selected on the basis of the absence of distracting stimuli. We anticipated that in some cases, a “control” condition may consist of a task that was not entirely free from distraction (e.g., requiring participants to memorize a number while eating, as in [9]), but was less distracting than the intervention condition. In each eligible study, we identified control conditions as being the condition that was deemed the least distracting and had the lowest level of cognitive load. For example, in a comparison between watching TV and listening to music, the latter would be considered the control condition because it consists of auditory stimuli only, whereas the former consists of both visual and auditory stimuli. Two researchers independently identified the control condition for each study. In cases where a study measured distraction across an additional experimental factor (e.g., manipulating both energy density of a fixed meal as well as level of distraction [23]), effect sizes were taken separately for each level of the additional factor.

#### Outcomes

Studies were included if they measured concurrent or later intake as energy intake (in kcal or kJ) or as grams (g). Eligible studies measuring concurrent intake had to measure ad libitum intake during distraction (e.g., ad libitum consumption of snack foods while watching TV [24] or completing a computerized task [9]). Eligible studies measuring later intake were required to measure ad libitum intake at a later consumption episode and have not allowed ad libitum consumption during the distraction phase. This is because ad libitum intake under distraction could lead to differences in concurrent energy intake, which may then affect later energy intake. For both concurrent and later energy designs, eligible studies could have umeasured food or drink intake separately, or the combined intake of food and drink as the outcome measure.

#### Study design

Only studies that used an experimental design were included—both between and within-subject designs (and mixed designs) were eligible. Studies that measured intake in controlled laboratory settings or in real-world settings were eligible. Studies that had multiple conditions (e.g., multiple distractors across conditions) were eligible. For such studies, each comparison was extracted as a separate effect (e.g., distractor 1 compared with control, distractor 2 compared with control).

### Search process

A systematic review and meta-analysis that investigated the effect of distraction on concurrent and later intake [1] performed a detailed search of multiple databases up until 2012. We therefore searched PsycINFO, Medline, and PubMed, limiting searches to the years 2012–2024, and included pre-2012 studies from the previous systematic review and meta-analysis. Searches were conducted on 25 March, 2024 and were repeated on 6 December, 2024. Only studies published in English were eligible. See Supplementary Materials for full search strategies of these databases.

### Searching strategy

We used the following combination of search terms: (Food intake OR food consumption OR energy intake OR eating OR energy consumption) AND (distract∗ OR cognitive load OR attentional load). Additional relevant published literature was identified using a snowballing approach through searching reference lists of all eligible studies and conducting forward citation searches on Google Scholar. We asked authors of multiple eligible papers whether they had published any further studies that may be eligible. We also attempted to identify unpublished manuscripts by conducting additional searches of a pre-print server (PsyArXiv).

One author searched and screened articles from the review conducted by Robinson et al. [1] and all potentially eligible studies were screened by a second author. Electronic searches were conducted by 1 author who screened titles and abstracts, 20% were checked by a second author for consistency. Two authors conducted full-text screening. One author identified potentially eligible articles using the snowballing and grey literature approach—all articles were verified for inclusion/exclusion after being checked by a second independent author.

### Data extraction

Two authors independently extracted study information, including identification of the comparator for each study. Full extraction details are reported in Supplementary Materials; key extraction details are given in Tables 1 and 2 for the main study information extracted. These key extraction details were:

**TABLE 1:** Summary of concurrent intake studies

Authors (year of publication)	Country	Sample characteristics	Study design	Number of participants	Distraction manipulation ∗denotes the control condition identified ∗∗denotes condition not included in analyses	Category of distraction	Crockery/cutlery presentation	Control type	Intake findings
Bellisle and Dalix (2001) [25]	France	Adult females. Mean age = 35.1, SD = 9 Mean BMI = 21.3, SD = 1.9	Within-subjects	*n =* 41	Control∗: eating without any instructions. Distraction: eating while listening to a tape-recorded detective story. Attention∗∗: eating while paying attention to sensory qualities of the food. Group∗∗: eating within a group of 4 people.	Passive	Complex	Without additional task	Units: kJ Control condition: mean = 1998, SD = 589 Distraction condition: mean = 2299, SD = 589
Bellisle et al. (2004) [26]	France	Adult females recruited through posted advertisements in a hospital and nearby department stores. Mean age: = 29.9, SD = 1.4 Mean BMI = 22.3, SD = 0.2	Within-subjects	*n =* 48	Control∗: eating while left undisturbed in a quiet room. Distraction (television): eating while watching television. Distraction (audio recording): eating while listening to a detective story.	Television = passive Audio recording = passive	Complex	Without additional task	Units: kcal Control condition: mean = 419, SD = 138.56 Television: mean = 484, SD = 138.56 Audio recording: mean = 489, SD = 138.56
Bellisle et al. (2009) [27]	France	Adult females advertised in a hospital and medical school building. Mean age (low-restraint group) = 26.4, SD = 6.71 Mean age (high-restraint group) = 25.9, SD = 4.02 Mean age (total sample) = 26.15 Mean BMI (low-restraint group) = 21.5, SD = 1.79 Mean BMI (high-restraint group) = 22.4, SD = 2.24 Mean BMI (total sample) = 21.95	Mixed-design (distraction manipulation is within-subjects)	*n =* 40 Low restraint = 20 High restraint = 20	Control∗: eating alone in a quiet room, undisturbed. Television: eating alone while the television was on. Radio: eating alone while listening to a radio recording of a detective story. Group∗∗: eating in groups of 3. Television (with food commercials)∗∗: eating while viewing television advertisements for various foods.	Passive	Complex	Without additional task	Units: kJ Control (low restraint): mean = 2635, SD = 818.4 Control (high restraint): mean = 2636, SD = 711.07 Television (low restraint): mean = 2749, SD = 849.71 Television (high restraint): mean = 2564, SD = 661.88 Radio (low restraint): mean = 2744, SD = 912.32 Radio (high restraint): mean = 2500, SD = 688.71
Blass et al. (2006) [24]	United States	Undergraduate students. Mean age: not reported Mean BMI (macaroni and cheese condition): = 22.71, SD = 4.02 Mean BMI (pizza condition) = 26.35, SD = 6.66 Mean BMI (total sample) = 24.53	Mixed-design (distraction manipulation is within-subjects)	*n =* 20 Pizza = 10 Macaroni and cheese = 10	Control∗: eating while listening to classical music. Television condition: consumed food while watching a TV show.	Passive	Pizza = simple Macaroni cheese = complex	With additional task	Units: kcal Control (macaroni and cheese): mean = 342.8, SD = 197.99 Television (macaroni and cheese): mean = 586.21, SD = 366.92 Control (pizza): mean = 715.7, SD = 133.99 Television (pizza): mean = 959.14, SD = 232.96
Boon et al. (1997) [2]—study 1	The Netherlands	Female undergraduates. Mean age (restrained group) = 21.1, SD = 1.5 Mean age (unrestrained group) = 21.6, SD = 2.1 Mean BMI (unrestrained group) = 23.1, SD = 2.0 Mean BMI (unrestrained group) = 21.6, SD = 2.1	Between-subjects	*n =* 55 No distraction (restrained) = 11 No distraction (unrestrained) = 16. Distraction (restrained) = 13 Distraction (unrestrained) = 15	Control∗: eating alone with no radio conversation. Distraction: eating alone while listening to a radio conversation. Participants were asked to pay full attention to the conversation and to count the number of animal words figuring in the conversation.	Cognitively demanding	Complex	Without additional task	Units: g Control (restrained eaters): mean = 218.3, SD = 111.3 Control (unrestrained eaters): mean = 188.3, SD = 98.5 Distraction (restrained eaters): mean = 249.3, SD = 82.5 Distraction (unrestrained eaters): mean = 244.6, SD = 126.3
Boon et al. (1997) [2]—study 2	The Netherlands	Female undergraduates Mean age: not reported Mean BMI: not reported	Between-subjects	*n =* 49 total No distraction (unrestrained) = 10 No distraction (restrained) = 13). Distraction (restrained) = 11 Distraction (unrestrained) = 15	Control∗: eating alone with no radio conversation. Distraction: eating alone while listening to a radio conversation. Participants were asked to pay full attention to the conversation and to count the number of animal words figuring in the conversation.	Cognitively demanding	Complex	Without additional task	Units: g Control (restrained eaters): mean = 143.0, SD = 81.7 Control (unrestrained eaters): mean = 138.9, SD = 86.9 Distraction (restrained eaters): mean = 166.8, SD = 86.2 Distraction (unrestrained eaters): mean = 158.7, SD = 93.1
Boon et al. (2002) [28]	The Netherlands	Female undergraduates Mean age (restrained group) = 21.1, SD = 2.4 Mean age (unrestrained group) = 21.2, SD = 2.3 Mean BMI (restrained group) = 23.3, SD = 2.5 Mean BMI (unrestrained group) = 20.6, SD = 1.8.	Between-subjects The study also manipulated perceived calorie content of the test food (low calorie, high calorie).	*n =* 115 total No distraction (high calorie restrained) = 14 No distraction (high calorie unrestrained) = 14 No distraction (low calorie restrained) = 15 No distraction (low calorie unrestrained) = 15 Distraction (high calorie restrained) = 14 Distraction (high calorie unrestrained) = 14 Distraction (low calorie restrained) = 13 Distraction (low calorie unrestrained) = 16	Control∗: eating alone with no radio conversation. Distraction: eating alone while listening to a radio conversation. Participants were asked to pay full attention to the conversation and to count the number of animal words figuring in the conversation.	Cognitively demanding	Complex	Without additional task	Units: g Control (high calorie + restrained eaters) mean = 154.6, SD = 98.9 Control (high calorie + unrestrained eaters): mean = 176.0, SD = 77.5 Control (low calorie + restrained eaters): mean = 186.0, SD = 75.3 Control (low calorie + unrestrained eaters): mean = 175.1, SD = 97.0 Distraction (high calorie + restrained eaters): mean = 274.6, SD = 96.9 Distraction (high calorie + unrestrained eaters): mean = 192.5, SD = 76.5 Distraction (low calorie + restrained eaters): mean = 234.9, SD = 95.2 Distraction (low calorie + unrestrained eaters): mean = 261.0, SD = 78.2
Hetherington et al. (2006) [29]	United Kingdom	University students Mean age = 28.3, SEM =1.7 Mean BMI = 23.87, SEM = 0.8	Within-subjects	*n =* 37	Control∗: eating alone. Television: eating alone while watching a television program. Strangers∗∗: eating in the presence of strangers Friends∗∗: eating in the presence of friends.	Passive	Complex	Without additional task	Units: kJ Control: mean = 3861, SD = 1216.55 Television: mean = 4350, SD = 1532.86
Long et al. (2011) [30]	United Kingdom	Female university students Mean age = 21.2, SE = 0.7 Mean BMI = 23.8, SE = 0.64	Within-subjects	*n =* 27	Control∗: eating alone without any distractions. Distraction: eating while listening to an extract from Jane Austin’s Pride and Prejudice Focused attention∗∗: eating while attending to the sensory characteristics of the food being consumed.	Passive	Complex	Without additional task	Units: g Control: mean = 425.8, SD = 177.71 Distraction: mean grams = 513.4, SD = 195.38
Martin et al. (2009) [31]	United States	Males and females Mean age = 31.9 Mean BMI = 25.8	Within-subjects	*n =* 48	Control∗: eating alone without distraction. Reading: eating while reading provided material. Television—no adverts: participants viewed a TV program without ads while eating. Participants were informed that they would be asked several questions about the material they had read or viewed in the reading/or TV condition. Television – adverts∗∗: participants viewed a TV program interspersed with food and nonfood adverts.	Reading: physically demanding. Television: cognitively demanding	Complex	Without additional task	Units: kcal Control: mean =1053, SD = 911.2 Reading: mean kcal = 995, SD = 1055.1 Television (no adverts): mean = 1028, SD = 1007.2
Kononova et al. (2018) [32]	United States	University students Mean age = 21.8, SD = 1.64 Mean BMI = 23.29, SD = 5.05	Between-subjects	*n =* 140 TV only = 35 TV + texting = 34 TV + texting + online reading = 37 TV + texting + online shopping = 34	TV only∗: eating while watching a TV show. TV + texting: eating while watching a TV show and texting. TV + texting + online reading: eating while watching a TV show, texting, reading a Wikipedia article, and filled out a quiz). TV + texting + online shopping: eating while watching a TV show, texting, and shopping on amazon website.	All conditions (other than control) = physically demanding	Simple	With additional task	Units: g Control (TV only): mean = 3.85, SD = 0.59 TV + texting: mean = 3.54, SD = 1.05 TV + texting + online reading: mean = 3.31, SD = 1.28 TV + texting + online shopping: mean = 3.58, SD = 0.94
Ding et al. (2019) [33]	New Zealand	Adults who responded to a poster advert Mean age = 25.79, SD = 4.87 Mean BMI = 21.75, SD = 2.75	Within-subjects	*n =* 43	Control∗: eating while in isolation. Distraction condition: eating while performing a computer-based task.	Physically demanding	Simple	Without additional task	Units: kJ Control: mean = 3105.17, SD = 1101.32 Distraction: mean = 3083.87, SD = 1162.03
Arch et al. study 3 (2016) [34]	United States	Adults (no specific group) Mean age = 20.78, SD = 3.87 Mean BMI = not reported	Between-subjects	*n =* 102 Mindfulness condition = 33 Distraction condition = 33 No-instruction control = 36	Control∗: eating while listening to an excerpt from a cognitive psychology textbook, paying attention to the recording. Distraction: eating while completing word puzzles. Mindfulness condition∗∗: eating while engaging in a mindfulness exercise.	Physically demanding	Complex	With additional task	Units: kcal Control: mean = 259.65, SD = 159.23 Distraction: mean = 251.20, SD = 142.28
Lyons et al. (2012) [35]	United States	Adults (no specific group) Mean age = 24.07, SD = 4.43 Mean BMI = 24.41, SD = 4.14	Between-subjects	*n =* 120 Television = 40 Video games = 40 Motion-controlled video games = 40	Television∗: eating while watching commercial-free TV shows. Video gaming: eating while playing a video game using a standard controller. Motion-controlled video game condition: eating while playing a video game using a motion controller.	Video games = physically demanding. Motion-controlled video games = physically demanding	Complex	With additional task	Units: kcal Television: mean = 716, SD = 407. Video games: mean = 747, SD = 540. Motion-controlled video games: mean = 553, SD = 498
Stämpfli and Brunner (2016) [36]	Switzerland	Members of a sensory consumer panel. Mean age = 46.35, SD =14.20. Mean BMI = not reported.	Between-subjects Environmental cue (type of screensaver) was also manipulated.	*n =* 128 Low-load white screensaver = 33 Low-load Giacometti screensaver = 31 High-load white screensaver = 33 High-load Giacometti screensaver = 31	Low cognitive load∗: eating while memorizing a 2-digit number. High cognitive load: eating while memorizing a 10-digit number.	Cognitively demanding	Simple	With additional task	Units: g Low cognitive load (White screensaver): mean = 13.88, SD = 9.94 High cognitive load (White screensaver): mean = 15.24, SD = 8.5 Low cognitive load (Giacometti screensaver): mean = 12.01, SD = 6.85 High cognitive load (Giacometti screensaver): mean = 10.96, SD = 5.12
Brunner study 4 [37] (2013)	Switzerland	University students Mean age = 28.0, SD = 7.96 Mean BMI = 21.8, SD = 2.82	Between-subjects Effort was also manipulated (using either sugar tongs or their fingers).	*n =* 97 Fingers and low load = 23 Fingers and high load = 26 Tongs and low load = 25 Tongs and high load = 23	Low cognitive load∗: eating while memorizing a 2-digit number. High cognitive load: eating while memorizing a 10-digit number.	Cognitively demanding	Fingers = simple Tongs = complex	With additional task	Units: g Low cognitive load (fingers): mean = 9.22, SD = 5.17 High load (fingers): mean = 8.27, SD = 5.39 Low cognitive load (tongs): mean = 6.96, SD = 5.10 High load (tongs): mean = 5.74, SD = 3.97
da Mata Gonçalves et al. (2019) [38]	Brazil	Young adults Mean age (BMI < 25 group) = 20.2, SD = 2.0. Mean age (BMI ≥ 25 group) = 21.9, SD = 3.0. Mean BMI (BMI < 25 group) = 20.7, SD = 1.9. Mean BMI (BMI ≥ 25) = 27.7, SD = 2.2.	Within-subjects	*n =* 62	Control∗: eating without using smartphones or any other distractors. Smartphone: eating while using smartphones. Reading: eating while having access to printed text of a magazine during the meal.	Smartphone = physically demanding. Reading = physically demanding	Complex	Without additional task	Units: kcal Control: mean = 535, SD = 164 Smartphone: mean = 591, SD = 203 Reading: mean = 622, SD = 226
Ogden et al. (2013) [6]	United Kingdom	Females Mean age (driving) = 22.38, SD = 4.93. Mean age (television) = 22.9, SD = 5.45. Mean age (alone) = 21.9, SD = 5.14. Mean BMI (driving) = 21.26, SD = 2.39. Mean BMI (television) = 22.69, SD = 5.16. Mean BMI (alone) = 22.56, SD = 4.25.	Between-subjects	*n =* 81 Driving = 21 Television = 20 Alone = 21 Social = 19	Alone (control)∗: eating while sitting alone. Driving simulator: eating while using a driving simulator. Television: eating while watching television. Social interaction∗∗: eating while talking with a researcher.	Driving simulator = physically demanding Television = passive	Simple	Without additional task	Units: g Control: mean = 18.21, SD = 20.9 Driving: mean = 14.02, SD = 10.34 Television: mean = 28.61, SD = 24.44
Van der Wal and van Dillen (2013) [3] study 3	The Netherlands	University students Mean age = 21, SD = 2.44. BMI = not reported	Within-subjects Type of butter used was also manipulated (salt-free or salty).	*n =* 17	Low load∗: eating while memorizing a 1-digit number. High load: eating while memorizing a 7-digit number.	Cognitively demanding	Simple	With additional task	Units: percentage of food consumed Low load (salt-free butter): mean = 44.95, SD = 14.64. High load (salt-free butter): mean = 46.95, SD = 16.62. Low load (salty butter): mean = 42.91, SD = 12.45. High load (salty butter): mean = 58.93, SD = 22.59
Liguori et al. (2020) [21]	United States	University students. Mean age = 20.2, SD = 1.4 Mean BMI = 23.0, SD = 3.8	Within-subjects	*n =* 119	Control∗: eating with no distraction in a private booth. Distraction: eating while completing a computerized rapid visual information processing task.	Physically demanding	Simple	Without additional task	Units: g Control: mean = 128, SD = 49 Distraction: mean = 115, SD = 60
Volz et al. study 1 [9] (2021)	United States	Female undergraduates. Mean age = not reported Mean BMI = not reported	Between-subjects	*n =* 187 total Control = 38 0-back = 32 1-back = 39 2-back = 39 3-back = 39	Control∗: eating while holding a 1-digit number in memory. Experimental tasks presented participants with food while completing the N-back task. All responding was done using a foot pedal. 0-back: responded “yes” when a particular letter (indicated ahead of time) was mentioned. 1-back: responded “yes” whenever the current letter was the same as the letter mentioned just before it. 2-back: responded “yes” when the letter was the same as the letter 2 before it. 3-back: responded “yes” when the letter was the same as the letter 3 before it.	Cognitively demanding	Complex	With additional task	Units: g Control: mean = 25.28, SD = 17.00 0-back: mean = 34.69, SD = 17.90 1-back: mean = 28.15, SD = 16.74 2-back: mean = 30.46, SD = 24.75 3-back: mean = 28.67, SD = 17.28
Volz et al. study 2 [9] (2021)	United States	Female undergraduate students Mean age = not reported Mean BMI = not reported	Between-subjects	*n =* 84 Control = 23 1-back = 21 2-back = 20 3-back = 20	Control∗: eating while holding a 1-digit number in memory. Experimental tasks presented participants with food while completing the N-back task. All responding was done using a foot pedal: 1-back: responded “yes” whenever the current letter was the same as the letter mentioned just before it. 2-back: responded “yes” when the letter was the same as the letter 2 before it. 3-back: responded “yes” when the letter was the same as the letter 3 before it.	Cognitively demanding	Simple	With additional task	Units: g Control: mean = 36.96, SD = 18.31 1-back: mean = 60.00, SD = 29.08 2-back: mean = 37.05, SD = 23.65 3-back: mean = 50.90, SD = 31.83
Volz et al. study 3 [9] (2021)	United States	Male and female undergraduates. Mean age = 22.29, SD = 7.2 Mean BMI = not reported	Between-subjects	*n =* 114 Control = 24 1-back = 32 2-back = 29 3-back = 29	Control∗: eating while holding a 1-digit number in memory. Experimental tasks presented participants with food while completing the N-back task. All responding was done using a foot pedal: 1-back: responded “yes” whenever the current letter was the same as the letter mentioned just before it. 2-back: responded “yes” when the letter was the same as the letter 2 before it. 3-back: responded “yes” when the letter was the same as the letter 3 before it.	Cognitively demanding	Simple	With additional task	Units: g Control: mean = 22.67, SD = 15.17 1-back: mean = 36.41, SD = 15.90 2-back: mean = 40.38, SD = 30.03 3-back: mean = 46.14, SD = 45.49
Volz et al. study 4 [9] (2021)	United States	Male and female undergraduates. Mean age = 22.84, SD = 7.14. Mean BMI = not reported.	Within-subjects	*n =* 57	Control∗: eating while holding a 1-digit number in memory. Experimental tasks presented participants with food while completing the N-back task. All responding was made verbally: 1-back: responded “yes” whenever the current letter was the same as the letter mentioned just before it. 2-back: responded “yes” when the letter was the same as the letter 2 before it. 3-back: responded “yes” when the letter was the same as the letter 3 before it.	Physically demanding	Simple	With additional task	Units: g Control: mean = 7.89, SD = 6.59 1-back: mean = 7.75, SD = 7.16 2-back: mean = 6.54, SD = 5.04 3-back: mean = 5.32, SD = 4.50
Volz et al. study 5 [9] (2021)	United States	Undergraduate students Mean age = 19.79, SD = 2.56 Mean BMI: not reported	Within-subjects	*n =* 115	Control∗: eating while holding a 1-digit number in memory. Experimental tasks presented participants with food while completing the N-back task. All responding was made verbally: 1-back: responded “yes” whenever the current letter was the same as the letter mentioned just before it. 2-back: responded “yes” when the letter was the same as the letter 2 before it. 3-back: responded “yes” when the letter was the same as the letter 3 before it.	Physically demanding	Simple	With additional task	Units: g Control: mean = 7.33, SD = 6.42 1-back: mean = 7.05, SD = 6.21 2-back: mean = 7.17, SD = 7.05 3-back: mean = 5.45, SD = 5.97
Volz et al. study 6 [9] (2021)	United States	Undergraduate students Mean age = 23.24, SD = 8.00 Mean BMI = not reported	Within-subjects	*n =* 74	Control∗: eating while holding a 1-digit number in memory. Experimental tasks presented participants with food while completing the N-back task. All responding was made verbally: 1-back: responded “yes” whenever the current letter was the same as the letter mentioned just before it. 2-back: responded “yes” when the letter was the same as the letter 2 before it. 3-back: responded “yes” when the letter was the same as the letter 3 before it. 4-back: responded “yes” when the letter was the same as the letter 4 before it.	Physically demanding	Simple	With additional task	Units: g Control: mean = 10.35, SD = 7.16 1-back: mean = 7.73, SD = 6.79 2-back: mean = 6.70, SD = 5.35 3-back: mean = 6.22, SD = 5.55 4-back: mean = 5.90, SD = 4.61
Volz et al. study 7 [9] (2021)	United States	Undergraduate students Mean age = 19.58, SD = 1.98 Mean BMI = not reported	Within-subjects	*n =* 64	Control∗: eating while holding a 1-digit number in memory. Experimental tasks presented participants with food while completing the N-back task. All responding was made using a foot pedal: 1-back: responded “yes” whenever the current letter was the same as the letter mentioned just before it. 2-back: responded “yes” when the letter was the same as the letter 2 before it. 3-back: responded “yes” when the letter was the same as the letter 3 before it.	Cognitively demanding	Simple	With additional task	Units: g Control: mean = 17.19, SD = 9.37 1-back: mean = 14.08, SD = 9.66 2-back: mean = 12.89, SD = 10.03 3-back: mean = 10.58, SD = 9.14
Mathiesen et al. (2022) [39]	Finland	Adults (no specific group) Mean age = 39, SD = 12.5 Mean BMI = not reported	Between-subjects	*n =* 248 Silent = 61 Cafeteria = 62 Slow = 63 Fast = 62	Control (silent)∗: eating while no sound played while the participant was in the restaurant. Cafeteria: eating while listening to sound recordings from restaurant/cafeteria environments. Slow: eating while listening to prerecorded jazz instruments (at 65 bpm). Fast: eating while listening to prerecorded jazz instruments (at 160 bpm).	Passive	Complex	Without additional task	Units: g Control: mean = 586.02, SD = 101.92 Cafeteria: mean = 598.45, SD = 102.68 Slow: mean = 604.04, SD = 108.5 Fast: mean = 604.67, SD = 92.91
Francis et al. (2017) [40]	Australia	First-year psychology students Mean age = 19.7, SD = 2.9 Mean BMI = 22.4, SD = 3.1	Between-subjects	*n =* 153 No-TV condition = 74 TV condition = 79	Control (no TV)∗: eating quietly without watching a TV program. TV: eating while watching a TV program.	Passive	Complex	Without additional task	Units: kJ Control (female): mean = 1778.93, SD = 1233.5 Control (male): mean = 3017.80, SD = 1280.42 TV (female): mean = 2016.32, SD = 1118.84 TV (male): mean = 2172.11, SD = 1079.34
Braude and Stevenson (2014) [41]	Australia	Female undergraduates Mean age (total sample) = 19.6, SD = 2.2 Mean BMI (single food group) = 21.9, SD = 2.0 Mean BMI (variety food group) = 22.3, SD = 2.7	Mixed-design (distraction manipulation was within-subjects) Presentation of food (single or variety of foods) was also manipulated.	*n =* 62 Single food group = 29 Variety food group = 33	Control∗: eating quietly without watching a TV program. TV: eating while watching a TV program.	Passive	Single food = Simple Variety food = Complex	Without additional task	Units: kJ Control (single food): mean = 514.8, SD = 424.2 Control (variety food): mean = 646.2, SD = 462.8 TV (single food): mean = 687.2, SD = 466.4 TV (variety food): mean = 727.3, SD = 413.2
Çetin et al. (2023) [42]	Turkey	Young female adults Mean age = 21.6, SD = 1.50 Mean BMI = 21.7, SD = 2.04	Within-subjects	*n =* 35	Control∗: eating while no music was playing. Classical music 60 dB: eating while classical music was played at 60 dB. Classical music 80 dB: eating while classical music was played at 80 dB. Rock music 60 dB: eating while rock music was played at 60 dB. Rock music 80 dB: eating while rock music was played at 80 dB.	Passive	Complex	Without additional task	Units: kcal Control: mean = 966.23, SD = 295.03 Classical music 60 dB: mean = 1047.27, SD = 313.49 Classical music 80 dB: mean = 1072.21, SD = 331.89 Rock music 60 dB: mean = 1069.09, SD = 313.49 Rock music 80 dB: mean = 1016.1, SD = 313.49
Kaiser et al. (2016) [43]	Germany	University students Mean age (total sample) = 23.03, SD = 2.53 Mean BMI (control) = 22.37, SD = 3.37 Mean BMI (background loudspeaker) = 23.27, SD = 3.01 Mean BMI (background headphones) = 22.71, SD = 3.20 Mean BMI (English vocal music) = 23.96, SD = 2.81 Mean BMI (German vocal music) = 22.87, SD = 4.89	Between-subjects	*n =* 147 Control = 29 Background loudspeakers = 29 Background headphones = 31 English vocal music = 29 German vocal music = 29	Control∗: eating in silence. Background loudspeaker: eating with instrumental background music via loudspeakers. Background headphones: eating with instrumental background music via headphones. English vocal music: eating while listening to English pop songs. German vocal music: eating while listening to German pop songs.	Passive	Complex	Without additional task	Units: g Control: mean = 642.31, SD = 181.03 Background loudspeaker: mean = 698.59, SD = 216.35 Background headphones: mean = 727.32, SD = 260.14 English music: mean = 700.52, SD = 222.76 German music: mean = 613.41, SD = 166.17
Mamalaki et al. (2017) [44]	Greece	Male participants Median age = 21 Median BMI = 23.7	Within-subjects	*n =* 26	Control∗: eating with another participant, but with no music. 60 dB: eating with another participant, while listening to music at 60 dB. 90 dB: eating with another participant, while listening to music at 90 dB.	Passive	Complex	Without additional task	Units: kcal Control: mean = 1079, SD = 330. 60 dB music: mean = 1064, SD = 324 90 dB music: mean = 1136, SD = 311
Rosenthal and Raynor (2017) [45]	United States	Adults (no specific group) Mean age = 22.3, SD = 3.7 Mean BMI = 21.6, SD = 2.3	Within-subjects Portion size was also manipulated (small compared with large).	*n =* 20	Control∗: eating while sitting quietly and engaging in no other activities. TV: eating while watching a TV show.	Passive	Complex	Without additional task	Units: kcal Control (small portion size): mean = 713.21, SD = 169.81 Control (large portion size): mean = 901.89, SD = 264.15 TV (small portion size): mean = 766.04, SD = 203.77 TV (large portion size): mean = 916.98, SD = 279.25
Hussain et al. (2021) [46]	United Kingdom	Adults (no specific group) Mean age = 26.18, SD = 13.02 Mean BMI = 21.72, SD = 10.77	Between-subjects	*n =* 100 No music = 34 Classical music = 33 Popular music = 33	Control (no music)∗: eating without listening to any music. Classical music: eating while listening to classical music. Popular music: eating while listening to popular music.	Passive	Complex	Without additional task	Units: kcal Control: mean = 218.86, SD = 143.23 Classical music: mean = 183.41, SD = 97.54 Popular music: mean = 173.68, SD = 125.72
Ward and Mann [5] study 1 (2000)	United States	Female undergraduate students Mean age = not reported Mean BMI = not reported	Between-subjects	*n =* 60 Low load = 30 High load = 30	Low load∗: eating while responding to a reaction time task using a foot button. High load: eating while watching a series of art slides which they were asked to memorize and respond to a reaction time task during the slide show, using a foot button.	Cognitively demanding	Complex	With additional task	Units: g Low load (unrestrained eaters): mean = 59.67, SD = 26.50 High load (unrestrained eaters): mean = 43.60, SD = 24.04 Low load (restrained eaters): mean = 37.98, SD = 19.53 High load (restrained eaters): mean = 52.53, SD = 25.46
Ward and Mann [5] study 2 (2000)	United States	Female adults Mean age = not reported. Mean BMI = not reported.	Between-subjects	*n =* 29 Low load = 15 High load = 14	Low load∗: eating while responding to a reaction time task using a foot button. High load: eating while watching a series of art slides which they were asked to memorize and respond to a reaction time task during the slide show, using a foot button.	Cognitively demanding	Complex	With additional task	Units: g Low load: mean = 46.70, SD = 29.00 High load: mean = 71.60, SD = 35.30
Lattimore and Maxwell (2004) [47]	United Kingdom	Female undergraduate students. Mean age = 23.6, SD = 7.7. Mean BMI = 23.4, SD = 3.6.	Between-subjects	*n =* 119 Ego-threat Stroop = 30 Color naming Stroop = 30 Ego-threat Stroop memorization = 30 Color naming Stroop memorization = 29	Low load∗: eating while completing either an ego-threat Stroop or a color-name Stroop. High load: eating while completing either an ego-threat Stroop (which required memorization) or a color-name Stroop (which required memorization).	Physically demanding	Simple	With additional task	Units: gEgo threat Stroop (restrained eaters): mean = 83.1, SD = 36.29 Ego-threat Stroop (unrestrained eaters): mean = 92.1, SD = 24 Color Stroop (restrained eaters): mean = 91.8, SD = 30.12 Color Stroop (unrestrained eaters): mean = 89.0, SD = 21.13 Ego-threat Stroop memorization (restrained eaters): mean = 114.6, SD = 51.2 Ego-threat Stroop memorization (unrestrained eaters): mean = 73.8, SD = 34.63 Color Stroop memorization (restrained eaters): mean = 75.3, SD = 20.14 Color Stroop memorization (unrestrained eaters): mean = 77.8, SD = 28.81
Shin (2024) [48]	South Korea	Adults (no specific group). Mean age = 24.96, SD = 1.36. Mean BMI = 23.08, SD = 0.94.	Within-subjects	*n =* 23	Control∗: eating without distraction. Radio: eating while listening to a radio at 45 dB. TV: eating while watching television. Smartphone: eating while watching television and while having use of smartphone.	Radio and TV = passive Smartphone = physically demanding	Complex	Without additional task	Units: g Control: mean = 536.65, SD = 217.87 Radio: mean = 558.13, SD = 196.87 TV: mean = 560.35, SD = 223.49 Smartphone: mean = 642.52, SD = 186.85
Mann and Ward (2004) [49]	United States	Female undergraduate students. Mean age = not reported Mean BMI = not reported.	Between-subjects	*n =* 101 Milkshake-salient (high load) = 27 Milkshake-salient (low load) = 31 Diet-salient (high load) = 22 Diet-salient (low load) = 21	Low load∗: eating while memorizing a 1-digit number. High load: eating while memorizing a 9-digit number.	Cognitively demanding	Simple	With additional task	Units: g Low load (diet-salient): mean = 164.43, SD = 89.63 Low load (Milkshake-salient): mean = 171.51, SD = 89.64 High load (diet-salient): mean = 109.08, SD = 90.48 High load (Milkshake-salient): mean = 198.05, SD = 90.15

**TABLE 2:** Summary of later intake studies

Authors (year of publication)	Country	Sample characteristics	Study design	Number of participants	Distraction manipulation ∗denotes the control condition identified ∗∗denotes condition not included in analyses.	Length of interval between eating episodes	Intake findings
Higgs (2015) [50] study 1	United Kingdom	Female university students Mean age = 20, SD = 1.7 Mean BMI = 22, SD = 2.4	Between-subjects	*n =* 39 Control = 13 Low distraction = 13 High distraction = 13	Control∗: consumed lunch without playing any game. Low distraction: consumed lunch while playing a computer game without an incentive. High distraction: consumed lunch while playing a computer game with a monetary incentive.	90 min	Units: g Control: mean = 21.4, SD = 17.29 Low distraction: mean = 29.8, SD = 17.38 High distraction: mean = 36.2, SD = 17.29
Higgs (2015) [50] study 2	United Kingdom	Female university students Mean age = 19.7, SD = 3.5 Mean = 22.1, SD = 3.4	Between-subjects	*n =* 63 Control = 21 Food-related TV = 21 Nonfood-related TV = 21	Control∗: consumed lunch while watching no TV. Food-related TV: consumed lunch while watching a food-related TV clip. TV: consumed lunch while watching a nonfood-related TV clip.	150 min	Units: g Control condition: mean = 67.4, SD = 39.98. Food-related TV: mean = 74.7, SD = 39.86 TV: mean = 82.8, SD = 39.74.
Morris et al. (2020) [23]	United Kingdom	Female sample Mean age = 20.58, SD = 2.53 Mean BMI = 22.91, SD = 3.87	Between-subjects Energy of fixed meal was manipulated (low energy or high energy)	*n =* 120 Low load (low energy) = 30 Low load (high energy) = 30 High load (low energy) = 30 High load (high energy) = 30	Low load∗: preload delivered while completing a low perceptual load task. High load: preload delivered while completing a high perceptual load task.	32.5 min	Units: kcal Low load (low energy drink): mean = 164.81, SD = 71.26. High load (low energy drink): mean = 129.22, SD = 70.84. Low load (high energy drink): mean = 96.54, SD = 49.22. High load (high energy drink): mean = 138.98, SD = 62.30
Whitelock et al. (2018) [51] study 1	United Kingdom	Adults (no specific group) Mean age (normal control) = 27.57, SD = 11.99 Mean age (headphone control) = 29.16, SD = 10.63 Mean age (focused attention) = 29.91 SD = 10.50 Mean BMI (normal control) = 26.02, SD = 3.21 Mean BMI (headphone control) = 25.27, SD = 2.94 Mean BMI (focused attention) = 25.97, SD = 3.98	Between-subjects	*n =* 108 Normal control = 37 Headphone control = 37 Focused attention = 34.	Control∗: participants consumed a fixed lunch meal without headphones. Headphone condition: participants listened to a description of the migration and breeding pattern of cuckoo birds while eating a fixed lunchtime meal. Focused attention∗∗: participants listened to instructions to attend to their food while eating a fixed lunchtime meal.	180 min	Units: kcal Control (males): mean = 470.43, SD = 243.71. Control (females): mean = 286.05, SD = 147.67. Headphone (males): mean = 412.69, SD = 214.56. Headphone (females): mean = 300.96, SD = 153.55.
van Meer et al. (2023) [52]	The Netherlands	University students Mean age = 22.30, SD = 4.98 Mean BMI = not measured	Between-subjects	*n =* 116 Control = 58 Driving = 58	Control∗: consumed snack food while acting as passenger in a driving simulator. Driving: consumed snack food while acting as a driver in a driving simulator.	5 min	Units: kcal Control: mean = 72.9, SD = 24.7. Driving: mean = 84.3, SD = 31.1
Duif et al. (2020) [4]	The Netherlands	Adults (no specific group) Mean age = 22.5, SD = 3.5 Mean BMI = 21.9, SD = 1.89	Within-subjects	*n =* 41	Low load∗: participants completed a low-load categorical visual detection task while receiving a fixed amount of chocolate milk. High load: participants completed a high-load categorical visual detection task while receiving a fixed amount of chocolate milk.	45 min	Units: g Low load: mean = 65.6, SD = 37.78. High load: mean = 68.1, SD = 43.54
Higgs and Woodward (2009) [15]	United Kingdom	Female university students Mean age = 19, SD = 1 Mean BMI = 21.7, SD = 1.75	Within-subjects	*n =* 16	Control∗: consumed fixed laboratory lunch without watching television. Television: consumed fixed laboratory lunch while watching television.	150 min	Units: g Control: mean = 56.54, SD = 18.96. TV: mean = 68.83, SD = 21.33.
Mittal et al. (2011) [53] study 1	Australia	Female university students Mean age (experimental group) = 20.8, SD = 3.8 Mean age (control group) = 20.3, SD = 3.9 Mean BMI (experimental group) = 21.5, SD = 1.6 Mean BMI (control group) = 21.8, SD = 2.1	Between-subjects	*n =* 32 Control = 16 Television = 16	Control∗: consumed fixed laboratory snack without watching television. Television condition: consumed fixed laboratory snack while watching television.	45 min	Units: kJ Control: mean = 1354.9, SD = 335.6. Television: mean = 1584.6, SD = 516.4.
Mittal et al. (2011) [53] study 2	Australia	Female university students Mean age (boring condition) = 22.8, SD = 4.1 Mean age (sad condition) = 20.6, SD = 3.2 Mean age (funny condition) = 21.3, SD = 2.7 Mean age (control condition) = 20.6, SD = 2.7 Mean BMI (boring condition) = 21.4, SD = 2.1 Mean BMI (sad condition) = 21.2, SD = 1.5 Mean BMI (funny condition) = 21.7, SD = 1.9 Mean BMI (control condition) = 21.0, SD = 1.8	Between-subjects	*n =* 84 total Control condition = 21 Boring condition = 21 Sad condition = 21 Funny condition = 21	Control∗: consumed fixed laboratory snack in the absence of television. Boring condition: consumed fixed laboratory snack while watching boring television program. Funny condition: consumed fixed laboratory snack while watching funny television program. Sad condition: consumed fixed laboratory snack while watching sad television program.	45 min	Units: kJ Control: mean = 2147.9, SD = 527.2. Boring: mean = 2507.0, SD = 438.2. Sad: mean = 2842.0, SD = 452.4. Funny: mean = 2637.6, SD = 540.3.
Oldham-Cooper et al. (2010) [54]	United Kingdom	Male and female participants Mean age (distraction condition) = 28.1, SD = 17.2 Mean age (no-distraction condition) = 26.3, SD = 15.0 Mean BMI (distraction condition) = 23.1, SD = 3.0 Mean BMI (no distraction condition) = 23.6, SD = 3.07	Between-subjects	*n =* 44 Control condition = 22 Distraction condition = 22	Control condition∗: consumed a fixed lunch, attending to sensory characteristics of the foods. Distraction condition: consumed a fixed lunch while playing solitaire.	50 min	Units: g Control: mean = 27.1, SD = 26.4. Distraction: mean = 52.1, SD = 45.1.

#### Bibliographic information and study characteristics

Authors; year of publication; country where the study was conducted; sample characteristics (e.g., age, BMI); study design (e.g., within-subjects, between-subjects, mixed design); number of participants (total and per condition); distraction manipulation (detailing the control and distractor tasks used); category of distraction task (*concurrent energy intake studies only*): passive (e.g., TV viewing), cognitively demanding (e.g., completing the N-back task), physically demanding (e.g., participating in a driving simulator); crockery/cutlery presentation (*concurrent energy intake studies only*): complex (food presented across multiple pieces of crockery or with cutlery) or simple (food presented on 1 piece of crockery, without cutlery); control type (*concurrent energy intake studies only*): with additional task to eating (e.g., being instructed to eat while memorizing a number) or without additional task to eating (e.g., being instructed to eat without any other task); length of interval between eating episodes (*later energy intake studies only*).

#### Outcome data

Intake: amount (in kilocalories, kilojoules, or grams) consumed in the experimental condition(s) and control condition either during the experimental manipulation of distraction (for concurrent intake studies) or at the subsequent eating episode (for later intake studies).

### Missing information

Any missing data on outcome variables were extracted from articles using WebplotDigitizer [55]. If this was not possible, data were requested by authors of the article.

### Risk of bias

We assessed risk of bias using a modified tool, informed by other risk of bias tools [56,57]. This was adapted to include methodological considerations for laboratory eating experiments (see [58] as an example which uses this approach). Studies were considered as higher in risk of bias if they: *1)* examined self-reported intake (as opposed to objective measurement), *2)* did not include key eligibility criteria, *3)* had inadequate information regarding study methodology, *4)* did not use random allocation to experimental conditions, *5)* did not address the influence of participant awareness of study aims, *6)* had a small number of participants per condition, *7)* did not include a preregistered protocol, *8)* did not require participants to abstain from eating before the test session. Greater scores were indicative of a greater number of bias indicators. See Supplementary Materials for further details of the bias indicators used, along with a breakdown of scores for each study.

### Analyses

#### Primary analyses

Analyses were conducted using the “metaphor” [59], “dmetar” [60], “tidyverse” [61] and “janitor” [62] packages in R. We computed relevant effect sizes using the “escalc” function. We conducted generic variance inverse meta-analyses (random effects due to expected heterogeneity) with intake as the outcome variable. *I*^2^ was reported to characterize heterogeneity (>50% indicative of moderate heterogeneity, >75% indicative of substantial heterogeneity). The outcome effect was reported as a standardized effect size measure (SMD). For interpretation, positive SMDs indicate greater intake in the distraction group, compared with the control group. For studies using a within-subjects design, due to a lack of reporting of intraclass correlations between conditions, we imputed a correlation of *r* = 0.7 based on previous studies [63] and conducted sensitivity analyses across a range of correlations (*r* = 0.1 to *r* = 0.9). Because studies could contribute multiple effect sizes (e.g., multiple distraction groups compared with control [35]), multilevel meta-analysis was used.

#### Secondary analyses

##### Type of distractor task—concurrent energy intake studies only

We performed a subgroup analysis to investigate whether the type of distractor task moderated the effect of distraction on concurrent energy intake. Studies measuring the effect of distraction on concurrent energy intake were categorized into one of 3 groups; subgroup analyses were conducted. See below for details of each group: *Passive*—a distractor was present, but the participant was not required to actively engage with it (e.g., watching TV, listening to a radio program).

*Cognitively demanding*—a distractor was present, and instructions required the participant to engage with a task cognitively (e.g., completing a computerized cognitive load task but the task *does not* require the use of hand or mouth). For example, in [28], participants listened to a radio conversation and were asked to pay full attention to the conversation and count the number of animal words mentioned within the conversation, therefore making the task cognitively demanding. *Physically demanding*—a distractor was present, and the participant was required to engage with it physically (specifically, requiring the use of hands or mouth), which may interfere with the physical process of eating (e.g., completing a computerized cognitive load task which *does* require the use of hands). This analysis was performed by coding studies as using either a passive, cognitively demanding, or physically demanding task. The primary analysis, which tested the effect of distraction on concurrent intake, was performed again but with this coded factor as a moderator (the passive distractor category was used as the reference group). A significant moderation effect was broken down by comparing the effect of distraction on concurrent intake between each distractor type (passive compared with cognitively demanding; passive compared with physically demanding; cognitively demanding compared with physically demanding).

##### Type of control condition—concurrent energy intake studies only

We also compared the effect between studies that used a control condition which did not require participants to engage in an additional task while eating (e.g., consuming food while in isolation, as in [33]) with studies which implemented a control condition which required participants to engage in an additional task while eating [e.g., consuming food while memorizing a 2-digit number (as opposed to a 10-digit number), as in [36]]. This analysis was performed by coding studies as using either a distracting or non-distracting control task. The primary analysis which tested the effect of distraction on concurrent intake was repeated with this factor as a moderator.

##### Use of crockery and cutlery—concurrent energy intake studies only

We compared the effect between studies that presented food across multiple pieces of crockery *or* involved the use of cutlery (complex crockery/cutlery presentation), compared with studies that did not (simple crockery/cutlery presentation), as some studies have presented food across multiple pieces of crockery or with the use of cutlery [34], whereas others have not [6] and the former may increase attention paid to their food during eating. Moderation analysis of the primary outcomes was performed by coding studies as using a complex or simple presentation.

##### Dietary restraint—concurrent energy intake studies only

We compared the effect of distracted eating on concurrent intake by comparing participants higher vs. lower in dietary restraint (or restrained compared with unrestrained), measured using either the original restraint scale, the DEBQ or TFEQ. We tested whether there was a moderating effect by restraint level and, additionally, conducted a restraint level by measure type interaction to test whether the effect of restraint was dependent on the way in which restraint was measured. To break down this significant interaction, the effect of distraction on concurrent intake was observed separately in the following cases: high-restraint participants measured using the TFEQ; high-restraint participants measured using the DEBQ; low-restraint participants measured using the TFEQ; low-restraint participants measured using the DEBQ. We did not perform this analysis for later intake studies due to the small number of studies of this type measuring restraint.

##### Comparison of older and newer effect sizes—concurrent and later energy intake studies

We compared the effect between studies identified in the original systematic review and studies published after [1] by including time point as a moderator in the primary analysis. We further analyzed this by performing a metaregression, investigating year of publication as a continuous variable.

##### Intermeal interval—later energy intake studies only

We performed a metaregression to examine whether length of the intermeal interval between consumption of the fixed meal and ad libitum consumption predicted the effect of distraction on later intake.

### Influential cases and publication bias

We examined any influential cases using difference in beta values (DFBETAs) >1, which is indicative of a >1 change in the SD of the estimated coefficient after removal of the effect. Boxplots were created for effect sizes to identify outliers. Visual inspection of funnel plots identified potential publication bias and this was formally tested using Egger’s test [64] and Trim and Fill procedure [65].

*P*-curve analysis was also conducted to test for evidential value [66]. This was conducted only for significant effects from the primary analyses. See Supplementary Materials for these results.

We also conducted sensitivity analyses on primary outcomes by removing studies with high bias scores. Bias scores ranged from 2 to 5 for concurrent intake studies and 2 to 6 for later intake studies (both scored out of 7). See Supplementary Online Materials for bias scores of each individual study. We had originally planned to conduct an analysis whereby only studies free of bias were retained in the analysis, but this was not possible. Instead, we reran analyses by removing studies which scored highest across bias indicators (scores of 5 for concurrent intake studies and scores of 6 for later intake studies). All extracted data used for analyses is available here https://osf.io/trp7x/.

## Results

A total of 50 eligible studies from 37 papers were included in the review and meta-analysis, producing a total of 111 effect sizes. Forty studies (95 effects) were included for analysis of concurrent intake [compared with 10 studies (14 effects) from the previous meta-analysis [1]], and 10 studies (16 effects) were included for analysis of later intake [compared with 4 studies (6 effects) from [1]] (Tables 1 and 2). The majority of studies were conducted in Europe (*n =* 26), with other studies being conducted in the United States (*n =* 17), Australasia (*n =* 5), South America (*n =* 1), and Asia (*n =* 1). Mean sample BMIs ranged from 21to 28 in the concurrent intake studies and between 21 and 26 for later intake studies.

### Primary analysis—concurrent energy intake

**FIGURE 2:**
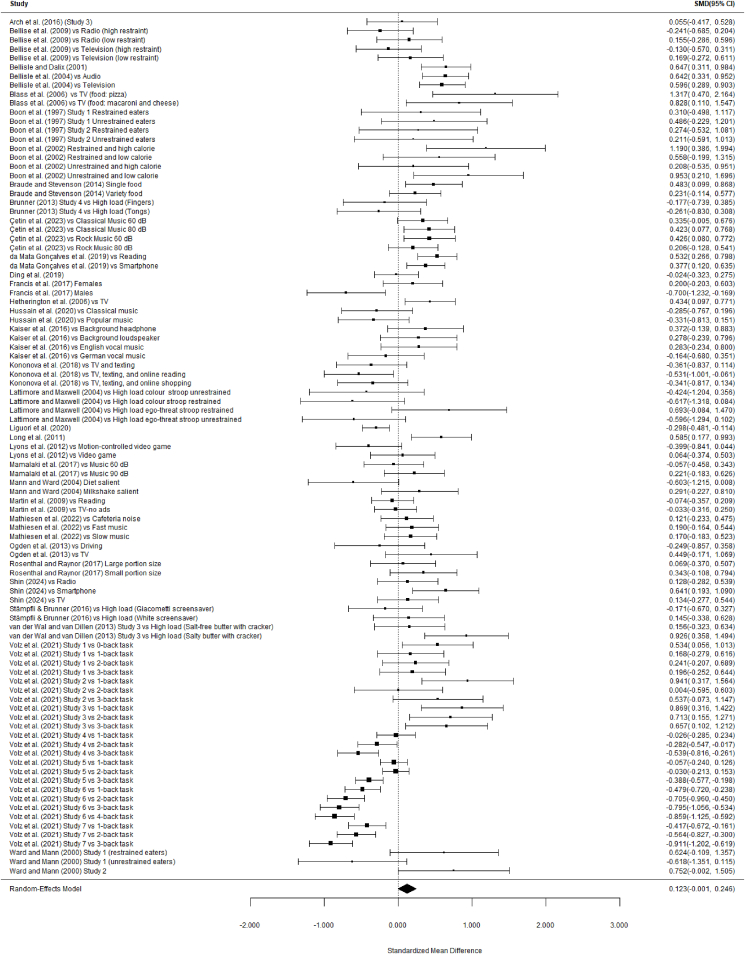
Forest plot of all effect sizes examining distraction on concurrent energy intake. CI, confidence interval; SMD, standardized mean difference.

**FIGURE 3:**
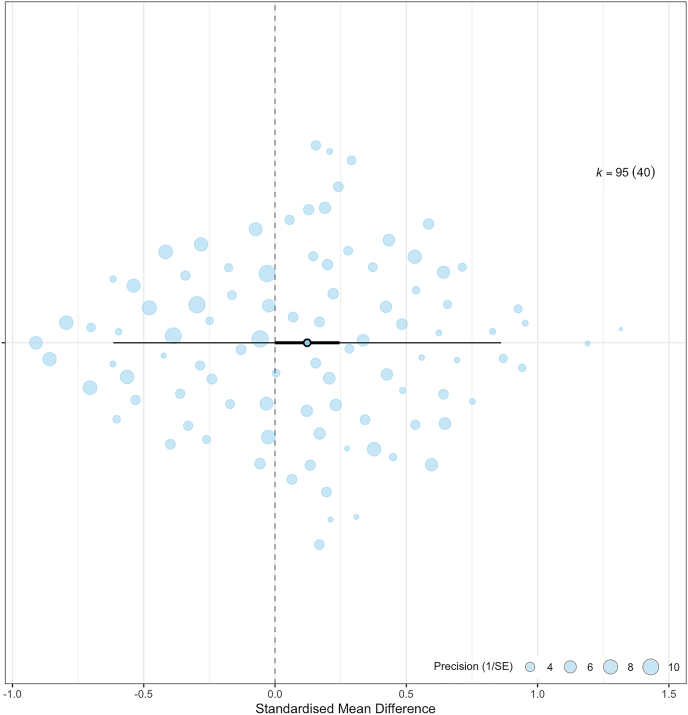
Orchard plot of all effect sizes examining distraction on concurrent energy intake. *K* = 95(40) refers to the number of effects and studies included. The plot presents 95% the confidence interval (bold line) and 95% prediction interval for the overall effect. The prediction interval estimates where the true effect of 95% of similar studies conducted in the future is expected to be.

Using loglikelihood ratio test, it was determined that a multilevel model (95 effect sizes within 40 studies) was a better fit of the data than a single-level model [*X*^2^(1) = 38.89, *P* < 0.001]. Although participants allocated to distraction during eating had a directionally higher concurrent intake, there was no statistically significant effect of distraction on concurrent intake {SMD = 0.123 [95% confidence interval (CI): –0.0007, 0.2457], *Z* = 1.95, *P* = 0.051, *I*^2^ = 78.7%}, see Figures 2 and 3 for forest plot and orchard plot of individual effects. Sensitivity analyses with different correlation coefficients for comparisons using a within-subject design, ranging from *r* = 0.1 to *r* = 0.9, did not substantially influence the effect size or statistical significance (ranges: SMD = 0.088 to 0.169, *P*s ≥ 0.051).

**FIGURE 4:**
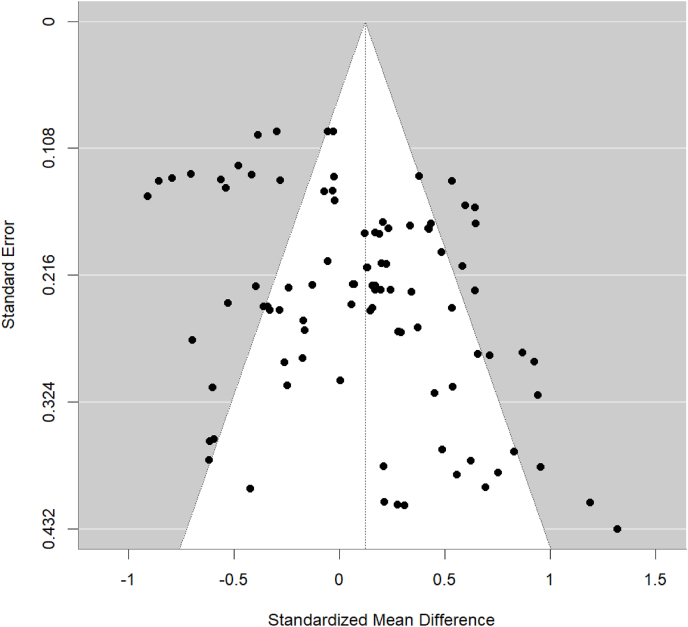
Funnel plot of the effect sizes against their precision for concurrent energy intake.

There were no influential cases identified; however, visual inspection of the funnel plot suggested asymmetry (Figure 4), indicative of potential publication bias. Egger’s test (*Z* = 3.65, *P* < 0.001) and Trim and Fill confirmed this {*n =* 15 imputed effects [adjusted SMD = –0.024 (95% CI: –0.122, 0.074)]}.

### Primary analysis—later energy intake

**FIGURE 5:**
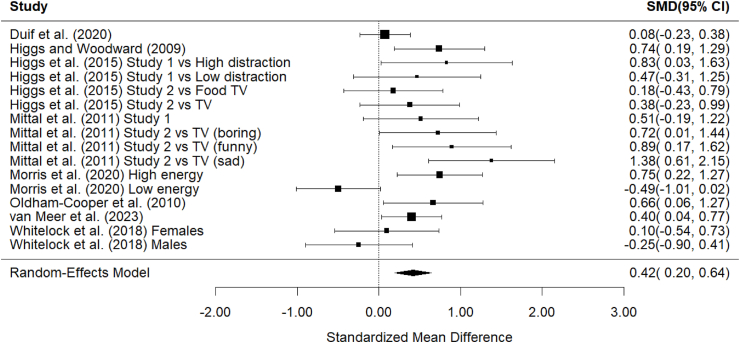
Forest plot of later energy intake following distraction compared with control. SMD, standardized mean difference; TV, television.

**FIGURE 6:**
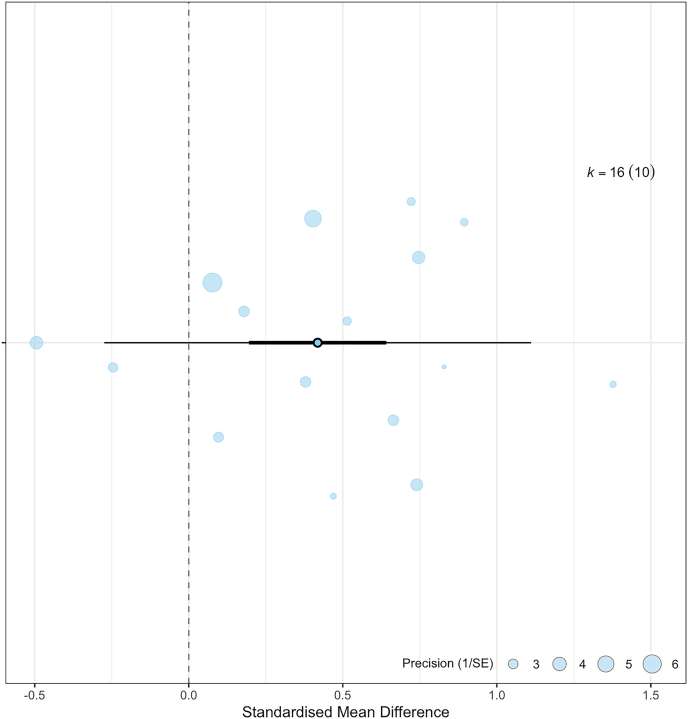
Orchard plot of later energy intake following distraction compared with control. *K* = 16(10) refers to the number of effects and studies included. The plot presents 95% the confidence interval (bold line) and 95% prediction interval for the overall effect. The prediction interval estimates where the true effect of 95% of similar studies conducted in the future is expected to be.

**FIGURE 7:**
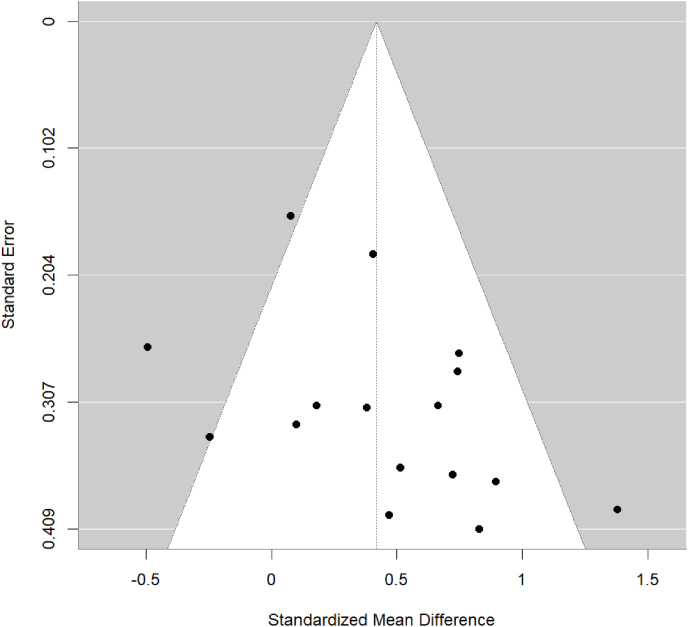
Funnel plot of the effect sizes against their precision for later energy intake.

Using loglikelihood ratio test, there was no evidence that a multilevel model was a better fit of the data [*X*^*2*^(1) = 0.40, *P* = 0.529], and studies did not tend to contribute multiple effect sizes; therefore, a single-level model was retained. There was a significant effect on later intake [SMD = 0.419 (95% CI: 0.195, 0.642), *P* < 0.001, *I*^2^ = 57.65%], see Figures 5 and 6 for forest and orchard plots of individual effects. There were no influential cases or outliers. There was some evidence of funnel plot asymmetry identified by Egger’s test (*Z* = 1.75, *P* = 0.008; Figure 7), indicative of potential publication bias. Trim and fill imputed 3 effect sizes to the left side of the funnel plot which reduced the pooled effect [SMD = 0.313 (95% CI: 0.084, 0.542), *P* = 0.007], but not the statistical significance of the model.

### Risk of bias

Of the 50 studies included, 47 did not include key eligibility criteria, 1 provided inadequate information of the study methodology, 7 did not state that random allocation had occurred, 43 did not adequately deal with demand characteristics, 6 were deemed to have a small sample size, 49 studies did not include an adequate preregistration, and 25 did not require participants to abstain from eating before study sessions. Bias scores ranged from 2 to 5 for concurrent intake studies and 2 to 6 for later intake studies (scored out of 7). See Supplementary Online Materials for bias scores of each individual study.

#### Concurrent energy intake

Removing studies with a bias score of 5 (4 studies, 8 effect sizes) produced a similarly sized and significant effect of distraction on concurrent food intake [SMD = 0.120 (95% CI: 0.0006, 0.2396), *Z* = 1.97, *P* = 0.049, *I*^2^ = 75.6%]. However, sensitivity analyses revealed that the statistical significance of this effect varied by the within-subjects correlation inputted between 0.1 and 0.9 (*P* = 0.069, 0.039).

#### Later intake studies

Removing the 1 study (1 effect size) with a bias score of 6 did not affect the statistical significance of the effect of distraction on later intake [SMD = 0.415 (95% CI: 0.179, 0.652), *Z* = 3.45, *P* = 0.001, *I*^2^ = 60.62%]. Sensitivity analyses did not change the statistical significance of this effect.

### Moderator analyses

#### Type of distractor task—concurrent energy intake studies

There was a significant moderator effect of distractor task type [*X*^2^(2) = 7.33, *P* = 0.026]. For effects involving the use of a passive distractor, distraction led to significantly greater intake (compared with control) [*n =* 35: SMD = 0.272 (95% CI: 0.128, 0.417)]. However, the effect of distraction on intake was nonsignificant for effects using a cognitively demanding distractor [*n =* 33: SMD = 0.202 (95% CI: –0.028, 0.432)] and effects using a physically demanding distractor [*n =* 27: SMD = –0.139 (95% CI: –0.334, 0.057)]. The size of the distraction effect significantly differed between passive and physically demanding distractors (*P* = 0.011), and between cognitively demanding and physically demanding distractors (*P* = 0.043). There was no significant difference between passive and cognitively demanding distractors (*P* = 0.705).

#### Type of control condition—concurrent energy intake studies

There was a nonsignificant moderator effect for type of control condition [*X*^2^(1) = 3.38, *P* = 0.066]. For effects where the control condition used an additional task to eating, distraction did not lead to significantly greater intake [*n =* 46: SMD = 0.011 (95% CI: –0.209, 0.231)]. For effects where the control condition did not use an additional task, distraction led to significantly greater intake [*n =* 49: SMD = 0.216 (95% CI: 0.092, 0.339)].

#### Crockery/cutlery presentation—concurrent energy intake studies

There was a significant moderation effect of crockery/cutlery presentation [*X*^2^(1) = 3.96, *P* = 0.047]. For effects which used a simple crockery/cutlery presentation, distraction did not lead to significantly greater intake [*n =* 39: SMD = –0.020 (95% CI: –0.245, 0.206)]. For effects which used a complex crockery/cutlery presentation, distraction led to significantly greater intake [*n =* 56: SMD = 0.235 (95% CI: 0.125, 0.345)].

#### Dietary restraint—concurrent energy intake studies

There was a nonsignificant moderation effect by dietary restraint [*X*^2^(1) = 1.51, *P* = 0.219]. However, there was a significant interaction between dietary restraint and the type of restraint scale used (*Z* = 2.37, *P* = 0.018). In studies using the TFEQ (*n =* 42), there was a significant moderation by restraint level [*X*^2^(1) = 4.16, *P* = 0.041]. Specifically, for participants high in restraint, the effect size was SMD = –0.232 (95% CI: –0.554, 0.091), and for participants low in restraint, the effect size was SMD = –0.002 (95% CI: –0.433, 0.430). However, for studies using the restraint scale (*n =* 14), there was no significant moderation by restraint level [*X*^2^(1) = 2.66, *P* = 0.132]. For participants high in restraint, the effect size was SMD = 0.419 (95% CI: –0.007, 0.844), and for in low restraint the effect size was SMD = 0.027 (95% CI: –0.483, 0.537).

#### Old compared with new studies—concurrent energy intake studies

There was a significant moderation effect of older compared with newer studies [*X*^2^(1) = 8.95, *P* = 0.003]. The effect size in studies conducted before 2012 (*n =* 21) was SMD = 0.433 (95% CI: 0.217, 0.650), whereas the effect was SMD = 0.024 (95% CI: –0.108, 0.157) for studies conducted in 2012 or later (*n =* 74). Additionally, there was a significant negative association between year of study and effect size [*B* = –0.018 (95% CI: –0.034, –0.003), *P* = 0.022].

#### Intermeal interval—later energy intake studies

There was no association between intermeal interval (shortest = 5 min, longest = 180 min) and later intake [*B* = –0.002 (95% CI: –0.006, 0.003), *P* = 0.486].

#### Old compared with new studies—later energy intake studies

There was a significant difference in the effect sizes in old and new studies [*X*^2^(1) = 8.60, *P* = 0.003]. In old studies (*n =* 4), the effect size was SMD = 0.790 (95% CI: 0.518, 1.061). In newer studies (*n =* 6), the effect size was SMD = 0.219 (95% CI: –0.027, 0.466).

#### Exploratory analyses

Exploratory analyses were conducted to better understand whether the moderating effect of older compared with newer studies on the relationship between distraction and concurrent intake was explained by confounding methodological factors. For example, 32% of newer studies examined a passive distractor, compared with 52% of older studies. Therefore, we included the year of publication (continuous) and the type of distractor in the same model. For this analysis, we grouped passive and cognitively demanding distractor tasks together compared with physically demanding to maximize power, resulting in 2 levels. Findings revealed that with both variables entered into this model, the effect of distractor type remained significant, whereas the effect of publication year was no longer significant (Table 3). This suggests that the difference in effect size between older and newer studies may be partly explained by the difference in frequency of distractor task types used between older and newer studies. In a further model which included the type of distractor, year of publication, and crockery/cutlery presentation, effect estimates for the latter two factors were reduced and were not statistically significant predictors. Distractor type effect estimate was similar as in other analyses (SMD = –0.22) but was no longer statistically significant at *P* < 0.05.

**TABLE 3:** Exploratory analyses investigating the effect of distraction on concurrent intake with type of distraction and year of publication entered into the model

	*b* (SE)	95% CI	*Z*-value	*P* value
Intercept	28.64 (15.16)	(–1.07, 58.35)	1.89	0.059
Type of distractor task (reference category: physically demanding)	–0.24 (0.11)	(–0.46, –0.03)	–2.26	0.024
Year of publication (continuous)	–0.01 (0.01)	(–0.03, 0.00)	–1.88	0.061

Abbreviation: CI, confidence interval.

## Discussion

This systematic review and meta-analysis investigated the effect of distraction during eating on concurrent and later energy intake. A total of 50 studies were included—40 investigated the effect on concurrent energy intake, and 10 investigated the effect on later energy intake. Although it is widely believed that distraction during eating increases energy intake, in primary analyses we did not find convincing evidence that distraction increased concurrent energy intake. Studies examining the effects of distraction on later energy intake showed more convincing evidence of greater subsequent energy consumption after eating while distracted, although intermeal interval did not moderate this effect. Additional analyses revealed that, in the case of concurrent intake studies, the effect of distraction on intake was moderated by the type of distraction used—passive distractors resulted in increases to concurrent energy intake, whereas physically and cognitively demanding distractors did not increase intake. Other analyses failed to show that the effect of distraction during eating was reliably moderated by tother study methodological features and participant dietary restraint levels.

Previous research has shown that distracted eating can shift attention away from aspects of eating that can influence feelings of satiety [6], produce a reduction in sensory-specific satiety [67,68], and disrupt cognitive goals relating to eating [5]. We proposed that the level of distraction likely moderates the effect of distracted eating on food intake. Specifically, as the level of distraction increases, so should food intake. However, when distraction reaches an attentional demand that distracts away from the act of eating, the effect of distracted eating on food intake is reduced [6]. Findings from the present meta-analysis showed that the type of distraction moderated the effect of distraction on concurrent intake. Distraction tasks which did not require any active cognitive or physical effort (passive distractors—i.e., TV viewing, listening to music) did significantly increase food intake. However, cognitively demanding distractors did not produce greater food intake (although this was directionally similar to the finding of passive distractors). The effect was also nonsignificant in the case of physically demanding distractors; however, this was directionally in the opposite direction to the other 2 distractor types, with intake being greater in the control conditions. This latter finding suggests that when a distractor interferes with the motor actions required to perform the act of eating (as is the case for physically demanding distractors), distraction may no longer increase concurrent energy intake [6,9]. However, distractor tasks that occupy attentional resources, but which do not interfere with this motor action, may still increase energy intake. Thus, the effect of distraction on concurrent intake may depend on the degree to which a distractor hinders the act of eating through a competing motor task.

We also investigated whether studies using control conditions that presented a task additional to the act of eating (e.g., memorizing a number) compared with control conditions that did not present an additional task differed in the effect of distraction on concurrent intake. We reasoned that, when studies used a control with an additional task, the difference in attentional demand and distraction from eating between conditions within the same study would be less than using a control without an additional task. Findings revealed that type of control did not significantly moderate the effect of distraction on concurrent intake at p<0.05. However, distraction was associated with greater intake in studies that did not use an additional task, whereas the effect was nonsignificant for effects which did implement a control condition with an additional task. Our interpretation is that this directional moderation finding is in line with an account of distracted eating which argues that the level of attentional demand may be important in determining food intake [9].

The present study also investigated whether the effect of distraction on concurrent intake was moderated by dietary restraint. Researchers have previously suggested that distraction may increase energy intake because eating under distraction may result in attention being placed away from self-regulatory processes relating to weight control [9,15], although a previous meta-analysis failed to show that restraint status moderated this effect [1]. The current analysis was consistent with Robinson et al. [1], showing that restraint level did not reliably moderate the effect of distraction on energy intake. However, there was directional evidence that restrained eaters (when measured using the restraint scale) consume more food when distracted, whereas higher restraint measured using the TFEQ showed an opposite directional effect, but neither of these contrasts was statistically significant. Dietary restraint, when measured using the restraint scale, is associated with disinhibited eating [[11], [12], [13]], whereas the TFEQ may more accurately measure successful restraint [14]. Therefore, the effect of distraction may be greater in individuals who are susceptible to disinhibited eating in cases where cognitive goals are not attended to (i.e., when under distraction). However, this analysis was based on a small number of studies and lacked statistical power, meaning that further research is needed to ascertain the role of dietary restraint in distracted eating.

The effect of distraction during a meal on later intake is believed to be caused, in part, through impairments of episodic memory encoding and retrieval during eating, resulting in greater subsequent intake [69,70]. Findings in the present study are consistent with this theory, and are in line with a previous meta-analysis which revealed that distraction while eating leads to greater intake at a later eating episode [1]. Secondary analyses tested whether the interval between the eating episode under distraction and the subsequent eating episode affected the magnitude of this effect. Findings were not supportive of this and did not suggest that there was a relationship between intermeal interval and energy intake. However, the number of later energy intake studies was relatively small; therefore, future research may still wish to formally test the effect of manipulating intermeal interval on subsequent later energy intake.

We also found evidence to suggest that the effects of distraction on both concurrent and later intake observed in studies have become smaller over time. To investigate this further, we conducted an exploratory analysis with both distractor task type and year of publication as moderators in the same model for concurrent energy intake, as a greater proportion of older studies used a passive distractor task, compared with newer studies. Findings revealed that with task type included as a moderator, year of publication was no longer a significant predictor, suggesting that the larger effects observed in older studies may partly be explained by methodological differences. Despite year of publication not significantly predicting the effect of distraction on concurrent intake after accounting for this, the directional reduction of an effect in newer studies may be indicative of a decline effect [71]—the phenomenon that many effects published in the literature reduce over time, which may reflect differences in methodologies adopted, populations sampled or publication bias. We did however find evidence that effects of distraction on later energy intake remained significant after accounting for potential publication bias.

This review has limitations. First, we were not able to account for all potential confounders due to the studies available for meta-analysis. For example, mood can affect energy intake, and positive mood induction has been shown to lead to greater energy intake [72,73]. Some of passive distractors included in this review could elicit changes in mood (such as TV viewing [41] and music [74]), but studies did not routinely examine if this was the case. One possibility, therefore, could be that confounding variables (such as mood) may explain some of the moderating findings. Although studies did tend to select neutral distraction tasks, so we presume any effects on mood would be relatively subtle. Second, the study is limited to articles published in English, therefore limiting generalizability of findings. Third, although our inclusion criteria allowed for real-world studies, all studies included were in a laboratory environment—meaning that the effect in real-world settings is not known, and could be larger or smaller compared with being measured in a laboratory [75]. Lastly, this paper is also limited in understanding how distraction can affect energy intake because included studies consisted only of adult samples and tended to be from Western countries [76]. The extent to which findings would generalize to other populations is unclear. For example, the effect of TV viewing on energy intake was recently examined among children and adolescents and there was no evidence that distraction caused greater concurrent energy intake [77].

The present findings may have implications for public health advice. Presently, public health guidance recommends to minimize distractions while eating due to risks of overeating [7]. The current findings offer mixed support for this recommendation, highlighting important nuances. Specifically, although conventional passive distractors such as TV viewing appear to increase concurrent energy intake, other forms of distraction that require more focused engagement may not.

In conclusion, findings indicate that eating under distraction results in greater energy intake at a later eating episode. Distraction did not affect concurrent energy intake in pooled analyses, but specific forms of distraction (i.e., passive distractors, such as watching TV) were associated with increased concurrent energy intake.

## Author contributions

The authors’ responsibilities were as follows – TG, AJ, TM, KA, KT, ER: designed the research; TG: performed literature searches; TG, AJ, AF: conducted data extraction; TG, AJ: performed statistical analyses; TG, ER: wrote the paper; TG: primary responsible for final content; and all authors: read and approved the final manuscript.

## Declaration of generative AI and AI-assisted technologies in the writing process

No AI tool was used.

## Data availability

Data described in the manuscript will be publicly and free available without restriction at https://osf.io/trp7x/.

## Funding

ER is funded by the National Institute for Health and Care Research Oxford Health Biomedical Research Centre and a Medical Research Council (MRC) Programme grant. The views expressed are those of the author(s) and not necessarily those of the funder.

## Conflict of interest

ER reports that during 2014–2016, he was a named investigator on a project funded by Unilever and a project funded by the American Beverage Association. ER does not receive any financial awards or fees from the food industry. AJ has received funding (as a coinvestigator) on a grant funded by CAMARUS pharmaceuticals for work unrelated to this.The other authors report no conflicts of interest.
