# Supplementary material for: Eating while distracted: a systematic review and meta-analysis of the effect of distraction on concurrent and later energy intake in adults
Source: Am J Clin Nutr. 2026 Apr 16;123(6):101315. doi: 10.1016/j.ajcnut.2026.101315 (PMC13269349; doi:10.1016/j.ajcnut.2026.101315)
Supplement: multimedia component 1 [file mmc1.docx]

**Eating whilst distracted: A systematic review and meta-analysis of the effect of distraction on concurrent and later intake in adults.**

Thomas Gough

**Supplementary materials**

[Reporting of search strategies for databases 3](#_Toc224631089)

[Medline 3](#_Toc224631090)

[APA PsycINFO 3](#_Toc224631091)

[PubMed 3](#_Toc224631092)

[Additional Analyses 5](#_Toc224631093)

[Overview of planned secondary analyses 5](#_Toc224631094)

[P-curve analysis 5](#_Toc224631095)

[Exploratory analysis 6](#_Toc224631096)

[Extracted data 8](#_Toc224631097)

[Concurrent intake studies 8](#_Toc224631098)

[Later intake studies 69](#_Toc224631099)

[Risk of Bias indicators 84](#_Toc224631100)

[Bias score breakdown – concurrent intake studies 85](#_Toc224631101)

[Bias scores breakdown – later intake studies 88](#_Toc224631102)

[List of excluded studies with reasons for exclusions 91](#_Toc224631103)

[References 94](#_Toc224631104)

# Reporting of search strategies for databases

## Medline

Ovid MEDLINE(R) ALL <1946 to March 25, 2024>

1 ((Food intake or food consumption or energy intake or eating or energy consumption) and (distract* or cognitive load or attentional load)).mp. [mp=title, book title, abstract, original title, name of substance word, subject heading word, floating sub-heading word, keyword heading word, organism supplementary concept word, protocol supplementary concept word, rare disease supplementary concept word, unique identifier, synonyms, population supplementary concept word, anatomy supplementary concept word]

2 limit 1 to yr="2012 - 2024"

## APA PsycINFO

1.

(Food intake OR food consumption OR energy intake OR eating OR energy consumption) AND (distract* OR cognitive load OR attentional load)

https://liverpool.idm.oclc.org/login?url=https://search.ebscohost.com/login.aspx?direct=true&db=psyh&bquery=(Food+intake+OR+food+consumption+OR+energy+intake+OR+eating+OR+energy+consumption)+AND+(distract*+OR+cognitive+load+OR+attentional+load)&cli0=DT1&clv0=201201-202412&type=1&searchMode=And&site=ehost-live&scope=site&ssl=y

| **Database** | **Limiters Applied** |
| --- | --- |
| APA PsycInfo | Published: 20120101-20241231 |

## PubMed

| Query | Filters | Search Details |
| --- | --- | --- |
| (Food intake OR food consumption OR energy intake OR eating OR energy consumption) AND (distract* OR cognitive load OR attentional load) | from 2012 - 2024 | (("eating"[MeSH Terms] OR "eating"[All Fields] OR ("food"[All Fields] AND "intake"[All Fields]) OR "food intake"[All Fields] OR (("food"[MeSH Terms] OR "food"[All Fields]) AND ("consumptions"[All Fields] OR "economics"[MeSH Terms] OR "economics"[All Fields] OR "consumption"[All Fields])) OR ("energy intake"[MeSH Terms] OR ("energy"[All Fields] AND "intake"[All Fields]) OR "energy intake"[All Fields]) OR ("eating"[MeSH Terms] OR "eating"[All Fields]) OR (("energie"[All Fields] OR "energies"[All Fields] OR "energy"[All Fields]) AND ("consumptions"[All Fields] OR "economics"[MeSH Terms] OR "economics"[All Fields] OR "consumption"[All Fields]))) AND ("distract*"[All Fields] OR (("cognition"[MeSH Terms] OR "cognition"[All Fields] OR "cognitions"[All Fields] OR "cognitive"[All Fields] OR "cognitively"[All Fields] OR "cognitives"[All Fields]) AND "load"[All Fields]) OR (("attention"[MeSH Terms] OR "attention"[All Fields] OR "attentions"[All Fields] OR "attention s"[All Fields] OR "attentional"[All Fields] OR "attentive"[All Fields] OR "attentively"[All Fields] OR "attentiveness"[All Fields]) AND "load"[All Fields]))) AND (2012:2024[pdat]) |
| (Food intake OR food consumption OR energy intake OR eating OR energy consumption) AND (distract* OR cognitive load OR attentional load) | | ("eating"[MeSH Terms] OR "eating"[All Fields] OR ("food"[All Fields] AND "intake"[All Fields]) OR "food intake"[All Fields] OR (("food"[MeSH Terms] OR "food"[All Fields]) AND ("consumptions"[All Fields] OR "economics"[MeSH Terms] OR "economics"[All Fields] OR "consumption"[All Fields])) OR ("energy intake"[MeSH Terms] OR ("energy"[All Fields] AND "intake"[All Fields]) OR "energy intake"[All Fields]) OR ("eating"[MeSH Terms] OR "eating"[All Fields]) OR (("energie"[All Fields] OR "energies"[All Fields] OR "energy"[All Fields]) AND ("consumptions"[All Fields] OR "economics"[MeSH Terms] OR "economics"[All Fields] OR "consumption"[All Fields]))) AND ("distract*"[All Fields] OR (("cognition"[MeSH Terms] OR "cognition"[All Fields] OR "cognitions"[All Fields] OR "cognitive"[All Fields] OR "cognitively"[All Fields] OR "cognitives"[All Fields]) AND "load"[All Fields]) OR (("attention"[MeSH Terms] OR "attention"[All Fields] OR "attentions"[All Fields] OR "attention s"[All Fields] OR "attentional"[All Fields] OR "attentive"[All Fields] OR "attentively"[All Fields] OR "attentiveness"[All Fields]) AND "load"[All Fields])) |

Additional Analyses

## Overview of planned secondary analyses

*Secondary analyses (type of control condition – concurrent energy intake studies only).* We performed a sub-group analysis comparing studies which use a ‘no distraction’ control (control conditions which require participants to eat or drink alone with no other distractions) with studies which used a ‘distraction’ control condition (control conditions which require participants to participate in any activity other than eating or drinking).

Secondary analyses (*crockery/cutlery presentation – concurrent energy intake studies only*). We performed a sub-group analysis comparing studies using a ‘low crockery/cutlery presentation’ and studies using a ‘high crockery/cutlery presentation’. The former group refers to studies which served food/drink using only one piece of crockery (e.g., bowl, plate, glass) and which do not use cutlery. The latter group refers to studies which served food/drink using more than one piece of crockery *or* with the use of cutlery.

*Secondary analyses (dietary restraint – concurrent energy intake studies only)*. We performed a sub-group analysis comparing the effect of distraction on concurrent energy intake between low restraint/unrestrained participants and high restraint/restrained participants. Furthermore, additional analyses were conducted relating to the type of dietary restraint used. We performed an additional analysis comparing the effect of restraint status across different measures of dietary restraint – dietary restraint measured using the restraint scale vs dietary restraint measured using either the DEBQ or TFEQ.

Secondary analyses *(comparison of older vs newer effect sizes – concurrent and later energy intake studies*). We performed a sub-group analysis comparing the effect found in studies included in the meta-analysis by Robinson, Aveyard (1) (‘older’ effect sizes) and effect sizes published after the completion of the article searching conducted by Robinson, Aveyard (1) (‘newer’ effect sizes). This was done separately for concurrent energy intake and later energy intake studies. We further analysed this by performing a meta-regression, investigating year of publication as a continuous variable.

*Secondary analyses (inter-meal interval – later energy intake studies only).* For the effect of distraction on later energy intake only, we conducted a meta-regression to examine whether length of the inter-meal interval between consumption of the fixed meal and ad libitum consumption predicts the effect of distraction on later energy intake.

## P-curve analysis

*P-curve analysis – later intake studies*

As the pooled model for later intake studies was significant, we conducted a p-curve analysis to examine the evidential value (see figure S1). From the 7 significant effects, there was no evidence of right skew (p = .130), suggesting no evidential value from these studies. This means that the significant effect of distraction on later energy intake, may in part be due to selective reporting of studies or analyses. However, evidence from trim and fill procedures indicated that when adjusting for publication bias, the effect of distraction on later intake was still statistically significant. It should be noted that the likelihood of demonstrating evidential value is dependent on the number of p-values used in a p-curve analysis, with fewer p-values resulting in a lower probability of concluding there to be evidential value (2). Therefore, as the current analysis was based on only 7 p-values, this finding should be interpreted with caution. Additionally, the use of p-curve tests has been called into question, with recent analyses of these tests recommending against their use in their current form (3).

**Figure S1: P-curve from later energy intake studies.**


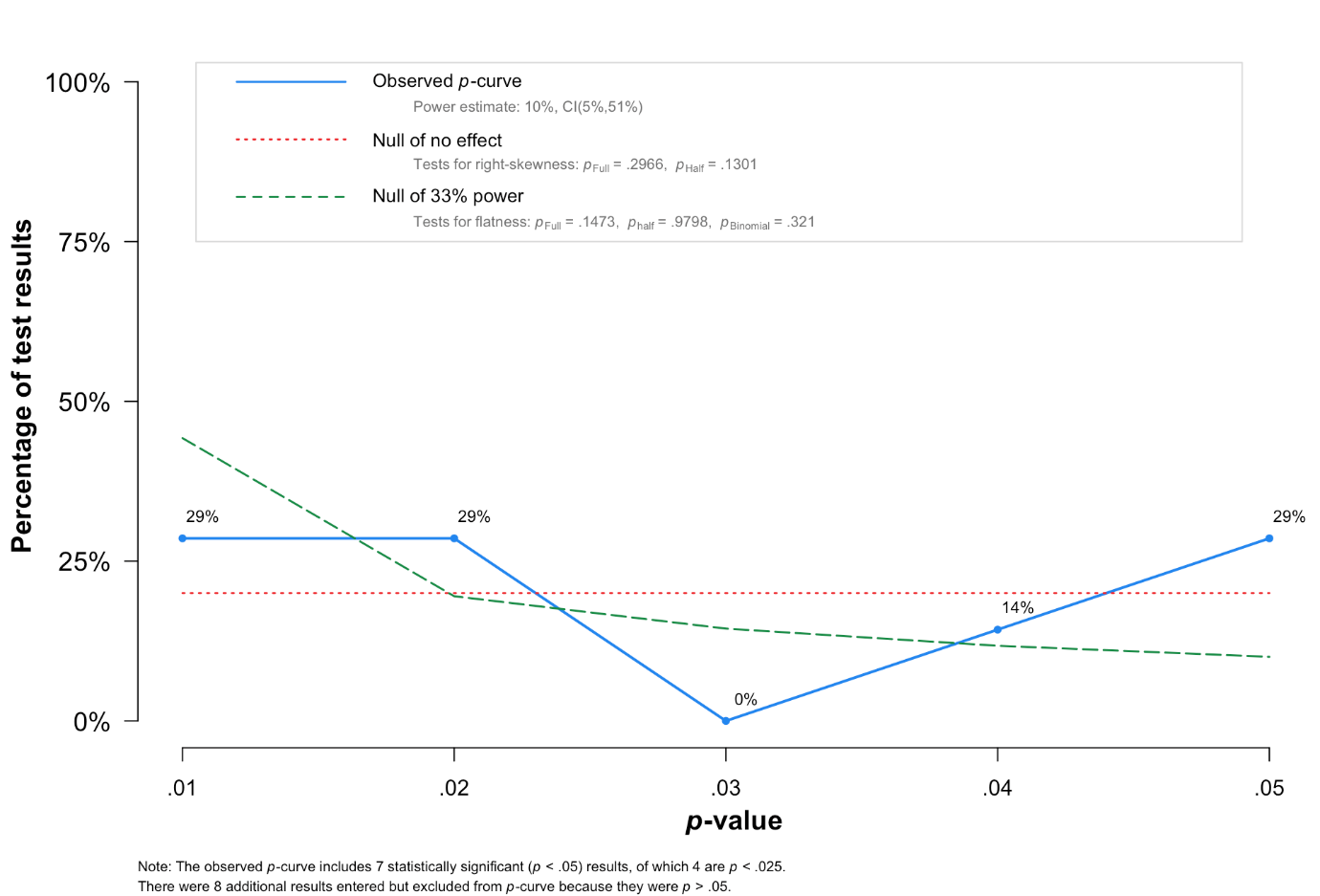


## Exploratory analysis

In addition to the exploratory analysis conducted in the manuscript, we, for completeness, present the statistical model with the inclusion of the significant moderator of crockery/cutlery presentation type, reported in table S1.

| Table S1. Exploratory analysis investigating the effect of distraction on concurrent intake with type of distraction, crockery/cutlery presentation level, and year of publication entered into the model. | | | | |
| --- | --- | --- | --- | --- |
|  | b(SE) | 95% CI | Z-value | p-value |
| Intercept | 25.16 (15.15) | (-4.54, 54.85) | 1.66 | .097 |
| Type of distractor task (reference category: physically demanding) | -0.22 (0.11) | (-0.43, 0.00) | -1.95 | .051 |
| Crockery/cutlery presentation (reference category: low) | -0.12 (0.11) | (-0.34, 0.10) | -1.10 | .271 |
| Year of publication (continuous) | -0.01 (0.01) | (-0.03, 0.00) | -1.64 | .100 |

# Extracted data

## Concurrent intake studies

Table S2. Extracted data for concurrent intake studies included in meta-analysis

| **Author and Year** | **Country** | **Study setting (laboratory or real-world)** | **Details of the sample group (e.g., university students)** | **Sample size** | **N per condition** | **Manipulation type (concurrent energy intake or later energy intake)** | **Study design (within-subjects, between-subjects, mixed design)** | **Exclusion criteria** |
| --- | --- | --- | --- | --- | --- | --- | --- | --- |
| Bellisle and Dalix (2001) (4) | France | Laboratory | Adult females recruited through posted advertisements in a hospital and nearby department stores. | 41 | 41 | Concurrent | Within-subjects | Participants had to be 18-60, have no declared pathology, and not presently under any medical treatment |
| Bellisle, Dalix, and Slama (2004) (5) | France | Laboratory | Adult females recruited through posted advertisements in a hospital and nearby department stores. | 48 | 48 | Concurrent | Within-subjects | Participants had to be female, aged between 18-50, normal weight (BMI between 18.5 and 24.9 kg/m^2^), no declared pathology, not presently under any medical treatment except for oral contraception. |
| Bellisle et al. (2009) (6) | France | Laboratory | Adult females recruited through posted advertisements in a hospital and medical school building | 40 | Low restraint = 20  High restraint = 20 | Concurrent | Mixed-Design | Participants had to be female, normal weight, no declared pathology, not pregnant. |
| Blass et al. (2006) (7) | USA | Laboratory | Undergraduate students from the University of Massachusetts-Amherst | 20 | Pizza = 10  Macaroni and cheese = 10 | Concurrent | Mixed-Design | Not stated |
| Boon et al. (1997) (8) (Study 1) | Netherlands | Laboratory | Female University students | 55 | No distraction (restrained) = 11  No distraction (unrestrained)= 16  Distraction (restrained) = 13  Distraction (unrestrained) = 15 | Concurrent | Between-subjects design | Not stated |
| Boon et al. (1997) (8) (Study 2) | Netherlands | Laboratory | Female University students | 49 | No distraction (unrestrained) = 10  No distraction (restrained) = 13)  Distraction (restrained) = 11  Distraction (unrestrained) = 15 | Concurrent | Between-subjects design | Not stated |
| Boon et al. (2002) (9) | Netherlands | Laboratory | Female University students | 115 | No distraction (high calorie restrained) = 14  No distraction (high calorie unrestrained) = 14  No distraction (low calorie restrained) = 15  No distraction (low calorie unrestrained) = 15  Distraction (high calorie restrained) = 14  Distraction (high calorie unrestrained) = 14  Distraction (low calorie restrained) = 13  Distraction (low calorie unrestrained) = 16 | Concurrent | Between-subjects design | Not stated |
| Hetherington et al. (2006) (10) | UK | Laboratory | University staff and students | 37 | 37 | Concurrent | Within-subjects | Participants had to have good health, have no food allergies, not be taking medications with the potential to affect appetite. |
| Long et al. (2011) (11) | UK | Laboratory | Female University students | 27 | 27 | Concurrent | Within-subjects | Participants had to have no history of clinically diagnosed eating disorders, not be taking any medication with the exception of oral contraception. |
| Martin et al. (2009) (12) | USA | Laboratory | Males and females | 48 | 48 | Concurrent | Within-subjects | Exclusion criteria: 1) Use of medications that affect eating behaviour or body weight; 2) diagnosis of a chronic disease such as diabetes, cardiovascular disease or cancer; 3) tobacco use; 4) refusal to eat the foods provided during the study; 5) irregular menstrual cycles or pregnancy |
| Kononova et al. (2018) (13) | USA | Laboratory | University students | 140 | TV only = 35  TV + texting = 34  TV + texting + online reading = 37  TV + texting + online shopping = 34 | Concurrent | Between-subjects | Exclusion criteria: Suffer from food allergies |
| Ding et al. (2019) (14) | New Zealand | Laboratory | Those who responded to poster advertisement | 43 | 43 | Concurrent | Within-subjects | Exclusion criteria: food allergies, vegan, vegetarian or kosher diet, medical condition or taking medicine which can affect appetite. Also excluded if they did not meet the following criteria: aged between 18 and 65, being in good health, consuming food regularly while working. |
| Arch et al. (2016) (15) (Study 3) | USA | Laboratory | University students | 102 | Mindfulness condition = 33  Distraction condition = 33  No-instruction control = 36 | Concurrent | Between-subjects | Not stated |
| Lyons et al. (2012) (16) | USA | Laboratory | Adults (no specific group) | 120 | Television = 40  Video games = 40  Motion-controlled video games = 40 | Concurrent | Between-subjects | Participants had to weigh < 300 pounds, have played video games ≥ 3 times over the previous year, have transportation to the study location, be willing to fast 2 hours before their appointment, be willing to be videotaped during their appointment |
| Stämpfli & Brunner (2016) (17) | Switzerland | Laboratory | Members of a sensory consumer panel | 128 | Low load white screensaver = 33  Low load Giacometti screensaver = 31  High load white screensaver = 33  High load Giacometti screensaver = 31 | Concurrent | Between-subjects | Not reported |
| Brunner (2013) (18) (Study 4) | Switzerland | Laboratory | University students | 97 | Fingers and low load = 23  Fingers and high load = 26  Tongs and low load = 25  Tongs and high load = 23 | Concurrent | Between-subjects | Not reported |
| da Mata Gonçalves et al. (2019) (19) | Brazil | Laboratory | Young adults | 62 | 62 in each | Concurrent | Within-subjects | Participants had to be regular user of smartphones, and not partake in any kind of food restriction |
| Ogden et al. (2013) (20) | UK | Laboratory | Females | 81 | Driving = 21  Television = 20  Social = 19  Alone = 21 | Concurrent | Between-subjects | Participants had to be female, aged over 18, and in possession of a full manual driving licence. |
| van der Wal et al. (2013) (21) (Study 3) | Netherlands | Laboratory | University students | 17 | 17 | Concurrent | Within-subjects | Not stated |
| Liguori et al. (2020) (22) | USA | Laboratory | University students | 119 | Control = 64  Distracted = 55 | Concurrent | Within-subjects | Exclusion criteria: Adhering to any dietary restrictions or diet, having any food allergies, and/or having any chronic or metabolic diseases |
| Volz et al. (2021) (23) (Study 1) | USA | Laboratory | Female undergraduate students | 187 | Control = 38  0-back = 32  1-back = 39  2-back = 39  3-back = 39 | Concurrent | Between-subjects | Participants had to have a score above (or below) the sample median on both the restraint and disinhibition TFEQ subscales |
| Volz et al. (2021) (23) (Study 2) | USA | Laboratory | Female undergraduate students | 84 | Control = 23  1-back = 21  2-back = 20  3-back = 20 | Concurrent | Between-subjects | Participants had to have a score above the sample median on both the restraint and disinhibition TFEQ subscales |
| Volz et al. (2021) (23) (Study 3) | USA | Laboratory | Male and female undergraduates | 114 | Control = 24  1-back = 32  2-back = 29  3-back = 29 | Concurrent | Between-subjects | None |
| Volz et al. (2021) (23) (Study 4) | USA | Laboratory | Male and female undergraduates | 57 | 57 | Concurrent | Within-subjects | None |
| Volz et al. (2021) (23) (Study 5) | USA | Laboratory | Undergraduate students | 115 | 115 | Concurrent | Within-subjects | None |
| Volz et al. (2021) (23) (Study 6) | USA | Laboratory | Undergraduate students | 74 | 74 | Concurrent | Within-subjects | None |
| Volz et al. (2021) (23) (Study 7) | USA | Laboratory | Undergraduate students | 64 | 64 | Concurrent | Within-subjects | None |
| Mathiesen et al. (2022) (24) | Finland | Research Restaurant | Adults (no specific group) | 248 | Silent = 61  Cafeteria = 62  Slow = 63  Fast = 62 | Concurrent | Between-subjects | Participants had to be aged 18-65, have self-reported normal senses of vision, smell, taste, and hearing, have no allergies to food served. |
| Francis et al. (2017) (25) | Australia | Laboratory | First year psychology students | 153 | TV condition = 79  No TV condition = 74 | Concurrent | Between-subjects | Participants had to have no eating disorders, not be taking medications or illnesses likely to affect appetite or cognition, be aged 17-30, have a self-reported normal BMI, have competence in English |
| Braude and Stevenson (2014) (26) | Australia | Laboratory | Female university students | 62 | 62 in each distraction condition  Single food group = 29  Variety food group = 33 | Concurrent | Mixed design - distraction manipulation is within-subjects | None |
| Çetin et al. (2023) (27) | Turkey | Laboratory | Young female adults | 35 | 35 | Concurrent | Within-subjects | Participants had to be: healthy females aged between 18 and 30, non-smokers, not dieting, not diagnosed with any metabolic disease, and no hearing loss, regular meal consumers.  Participants were excluded if they had a BMI outside of 18 to 25, were professional athletes, possessed food allergies, had extreme dislikes for specific foods or be pregnant or lactating, extreme disliking for any specific genre of music, scored higher than 9 on Beck's Depression Inventory. |
| Kaiser et al. (2016) (28) | Germany | Laboratory | University students | 147 | Control = 29  Background loudspeakers = 29  Background headphones = 31  English vocal music = 29  German vocal music = 29 | Concurrent | Between-subjects | Participants had to be aged 18-30, be enrolled in an agricultural science of economics university programme, or study biology, be non-vegetarian.  Exclusion criteria: smell, taste, or hearing impairments and a highly restrained eating score. |
| Mamalaki et al. (2017) (29) | Greece | Laboratory | Male participants | 26 | 60 dB = 26  90 dB = 26  Control = 24 (one pair of participants did not complete the control condition) | Concurrent | Within-subjects | Exclusion criteria: restrained eaters were excluded |
| Rosenthal and Raynor (2017) (30) | USA | Laboratory | Adults (no specific group) | 20 | 20 | Concurrent | Within-subjects | Participants had to: be aged 18-35, have a BMI between 18.5 and 24.9, be unrestrained eaters, report favourable preference for the test foods, eat before 10am on most days of the week, be able to complete all sessions within eight weeks, were non-smokers, were not taking medications than affected appetite or food intake, were not pregnant or breastfeeding, were not on a dietary plan or had dietary restrictions which prevented consumption of certain types and/or amounts of food.  Exclusion criteria: self-reported binge eating, being an athlete in training. |
| Hussain et al. (2021) (31) | UK | Laboratory | Adults (no specific group) | 100 | No music = 34  Classical music = 33  Popular music = 33 | Concurrent | Between-subjects | Exclusion criteria: Have been diagnosed with an eating disorder or have any food allergies/intolerances. |
| Ward and Mann (2000) (32) (Study 1) | USA | Laboratory | Female undergraduate students | 60 | Low load = 30  High load = 30 | Concurrent | Between-subjects | Not stated |
| Ward and Mann (2000) (32) (Study 2) | USA | Laboratory | Female adults | 29 | Low load = 15  High load = 14 | Concurrent | Between-subjects | Not stated |
| Lattimore and Maxwell (2004) (33) | UK | Laboratory | Female Undergraduate students | 119 | Ego threat Stroop = 30  Colour naming Stroop = 30  Ego threat Stroop memorisation = 30  Colour naming Stroop memorisation = 29 | Concurrent | Between-subjects | Participants had to have no food allergies, mental health problems, physical illness, and eating disorders |
| Shin (2024) (34) | South Korea | Laboratory | Adults (no specific group) | 23 | 23 | Concurrent | Within-subjects | Participants had to be aged 20-50, have a BMI between 18-25. Exclusion criteria: not in good health, on a weight-loss diet or trying to gain weight, using medication known to affect dietary intake or appetite, food allergies or food restrictions that could affect dietary intake, do not regularly eat 3 meals per day, scored higher than 29 on the EAT-40 and higher than 39 on the Zung Self-Rating Depression scale. |
| Mann and Ward (2004) (35) | USA | Laboratory | Female Undergraduate students | 101 | Milkshake-salient (high load) =  27  Milkshake-salient (low load) = 31  Diet-salient (high load) = 22  Diet-salient (low load) = 21 | Concurrent | Between-subjects | Participants had to score 16 or above on the restraint scale. Participants with a BMI (also assessed during pre-screening) below 18 or above 28 were deemed ineligible. |

| **Author and Year** | **Distraction manipulation**  ***denotes the control condition identified** | **Category of distraction task (concurrent energy intake studies only): passive, cognitively demanding, physically demanding).** | **Test foods. Reporting the foods used during distraction for concurrent energy intake studies, and test foods used for the fixed meal and subsequent eating episode for later energy intake studies.** | **Participants required to consume any amount of food? (concurrent energy intake studies only) Yes/No** | **Whether the amount of food consumed is visibly apparent (e.g., food wrappers remain in participants view) (concurrent energy intake study only) (Coded as Yes/No).** | **Whether the food/drink is presented as part of the study aims (concurrent energy intake study only) (coded as Yes/No).** | **Number of bowls, plates, or glasses used (concurrently energy intake studies only)** | **Whether food was consumed using cutlery -Yes/No (concurrent energy intake studies only)** |
| --- | --- | --- | --- | --- | --- | --- | --- | --- |
| Bellisle and Dalix (2001) (4) | Control*: eating without any instructions.  Distraction: eating whilst listening to a tape-recorded detective story. | Passive | 1kg Hachis Parmentier (casserole of ground beef and potatoes), 150 g fruit sherbet | No | No | Yes | Not stated (likely 2) | Yes (not explicitly stated, but likely due to the test food used) |
| Bellisle, Dalix, and Slama (2004) (5) | Control*: eating while left undisturbed in a quiet room.  Distraction (television): eating whilst watching television.  Distraction (audio recording): eating whilst listening to a detective story. | Passive | 1kg Hachis Parmentier (casserole of ground beef and potatoes), 150 g fruit sherbet | No | No | Yes | Not stated (likely 2) | Yes (not explicitly stated, but likely due to the test food used) |
| Bellisle et al. (2009) (6) | Control*: eating alone in a quiet room, undisturbed.    Television: eating alone while the television was on.  Radio: eating alone while listening to a radio recording of a detective story. | Passive | 1kg Hachis Parmentier (casserole of ground beef and potatoes), 400 g fruit sherbet | No | No | No aims mentioned to participants | Not stated (likely 2) | Yes (not explicitly stated, but likely due to the test food used) |
| Blass et al. (2006) (7) | Control*: eating while listening to classical music.  Television condition: consumed food while watching a TV show | Passive | Pizza condition: 12 inch DiGiornos, 4 cheese pizza cut into 8 slices.  Macaroni and cheese condition: Family Size, Stouffers, presented in a large bowl | No | No | No | Not stated (likely 1) | Yes, for macaroni and cheese |
| Boon et al. (1997) (8) (Study 1) | Control*: eating alone with no radio conversation.  Distraction: eating alone while listening to a radio conversation. Participants were asked to pay full attention to the conversation and to count the number of animal words figuring in the conversation. | Cognitively Demanding | Three bowls of ice cream, each containing 600 grams. Each bowl had one of three flavours: vanilla, strawberry, chocolate. | Yes | No | Yes | 3 | Yes |
| Boon et al. (1997) (8) (Study 2) | Control*: eating alone with no radio conversation.  Distraction: eating alone while listening to a radio conversation. Participants were asked to pay full attention to the conversation and to count the number of animal words figuring in the conversation. | Cognitively Demanding | Three bowls of ice cream, each containing 600 grams. Each bowl had one of three flavours: vanilla, strawberry, chocolate. | Yes | No | Yes | 3 | Yes |
| Boon et al. (2002) (9) | Control*: eating alone with no radio conversation.  Distraction: eating alone while listening to a radio conversation. Participants were asked to pay full attention to the conversation and to count the number of animal words figuring in the conversation. | Cognitively Demanding | Three bowls of ice cream, each containing 600 grams. Each bowl had one of three flavours: vanilla, strawberry, chocolate. | Yes | No | Yes | 3 | Yes |
| Hetherington et al. (2006) (10) | Control*: eating alone.  Television: eating alone while watching a television programme. | Passive | 10 slices of cheese (173 g), 5 bread rolls (161 g), spread (100g), 220 g coleslaw, 80 g potato crisps, 3 chocolate chip cakes (92 g), 3 almond slices (108 g), 3 chocolate cake bars (74 g), 80 g salad | No | Unclear | Yes | Not stated (likely more than 1) | Not stated |
| Long et al. (2011) (11) | Control*: eating alone without any distractions.  Distraction: eating while listening to an extract from Jane Austin's Pride and Prejudice | Passive | 750 g of cooked white wheat pasta and 500 g of Dolmio sauce | No | No | Yes | Not stated (likely 1) | Yes (not explicitly stated, but likely due to the test food used) |
| Martin et al. (2009) (12) | Control*: eating alone without distraction.  Reading: eating while reading provided material.  Television - no adverts: participants viewed a TV program without ads while eating. Participants were informed that they would be asked several questions about the material they had read or viewed in the reading/ or TV condition. | Reading: Physically Demanding.  Television: Cognitively Demanding | Baked potato chips, salsa, baby carrots, pretzel twists, fat-free ranch dressing, grilled chicken bites, corn chips, cheese dip, butter popcorn, salted mixed nuts, candy-coated chocolate bites, chocolate cake rolls with icing, breaded chicken bites, cheddar cheese, Swiss cheese, raisins, fruit punch, BBQ sauce, sweet and sour sauce | Yes | No | Yes | 16 | Yes (not explicitly stated, but likely due to the test food used) |
| Kononova et al. (2018) (13) | TV only*: eating while watching a TV show.  TV + texting: eating while watching a TV show and texting.  TV + texting + online reading: eating while watching a TV show, texting, reading a Wikipedia article, and filled out a quiz.  TV + texting + online shopping: eating while watching a TV show, texting, and shopping on amazon website. | All conditions (other than control) = Physically Demanding | Carrots, tomatoes, raw almonds, pringles, almond M&Ms, sugar candy | No | No | No | 1 | No |
| Ding et al. (2019) (14) | Control*: eating while in isolation.  Distraction condition: eating while performing a computer-based task. | Active and physically demanding | Ham and cheese Domino’s pizza | Yes | No | Not stated | 1 | No |
| Arch et al. (2016) (15) (Study 3) | Control condition*: eating while listening to an excerpt from a cognitive psychology textbook, paying attention to the recording.  Distraction: eating while completing word puzzles. | Active and physically demanding | M&Ms, Reese's Pieces, Lay's Potato Chips, Rold Gold Pretzels, Unsalted Almonds, Carrots Sticks | Yes | No | Yes | 6 | No |
| Lyons et al. (2012) (16) | Television*: eating while watching commercial-free TV shows.  Video gaming: eating while playing a video game using a standard controller.  Motion-controlled video game condition: eating while playing a video game using a motion-controller. | Television = passive  Video games = active and physically demanding.  Motion-controlled video games = active and physically demanding | Chips, baked chips, trail mix, chocolate candy, Coca-Cola, diet coke, mountain dew, bottled water | Not stated | No | Not stated | 8 | No |
| Stämpfli & Brunner (2016) (17) | Low cognitive load*: eating while memorising a 2-digit number.  High cognitive load: eating while memorising a 10-digit number. | Cognitively Demanding | Pringles | Yes | No | Yes | 1 | No |
| Brunner (2013) (18) (Study 4) | Low cognitive load*: eating while memorising a 2-digit number.  High cognitive load: eating while memorising a 10-digit number. | Cognitively Demanding | Dried apricots | Yes | No | Yes | 1 | This was experimentally manipulated |
| da Mata Gonçalves et al. (2019) (19) | Control*: eating without using smartphones or any other distractors.  Smartphone: eating while using smartphones.  Reading: eating while having access to printed text of a magazine during the meal. | Smartphone = Physically Demanding.  Reading = Physically Demanding | Soda, cookies, chocolate, unsweetened natural yogurt, water, toast, banana, apple | Yes | Some food yes (banana, apple) | Yes | Not stated (likely more than 1) | Yoghurt yes, otherwise no |
| Ogden et al. (2013) (20) | Control*: eating while sitting alone.  Driving simulator: eating while using a driving simulator.  Television: eating while watching television. | Driving simulator = Physically Demanding  Television = Passive | Hula Hoops (crisps) | Yes | No | Yes | 1 | No |
| van der Wal et al. (2013) (21) (Study 3) | Low load*: eating while memorising a one-digit number.    High load: eating while memorising a seven-digit number. | Cognitively Demanding | Crackers (saltiness was experimentally manipulated) | Yes | No | Yes | 1 | No |
| Liguori et al. (2020) (22) | Control*: Eating with no distraction in a private booth.  Distraction: Eating whilst completing a computerised rapid visual information processing task. | Physically Demanding | Miniature spinach and cheese quiches | No | No | No | 1 | No |
| Volz et al. (2021) (23) (Study 1) | Control*: eating while holding a one-digit number in memory.  Experimental tasks presented participants with food while completing the N-back task. All responding was done using a foot pedal.  0-back: responded 'yes' when a particular letter (indicated ahead of time) was mentioned.  1-back: responded 'yes' whenever the current letter was the same as the letter mentioned just before it.  2-back: responded 'yes' when the letter was the same as the letter two before it.  3-back: responded 'yes' when the letter was the same as the letter three before it. | Cognitively Demanding | Potato chips and French Onion dip | Yes | No | Yes | 2 | No |
| Volz et al. (2021) (23) (Study 2) | Control*: eating while holding a one-digit number in memory.  Experimental tasks presented participants with food while completing the N-back task. All responding was done using a foot pedal.  1-back: responded 'yes' whenever the current letter was the same as the letter mentioned just before it.  2-back: responded 'yes' when the letter was the same as the letter two before it.  3-back: responded 'yes' when the letter was the same as the letter three before it. | Cognitively Demanding | M&Ms | Yes | No | Yes | 1 | No |
| Volz et al. (2021) (23) (Study 3) | Control*: eating while holding a one-digit number in memory.  Experimental tasks presented participants with food while completing the N-back task. All responding was done using a foot pedal.  1-back: responded 'yes' whenever the current letter was the same as the letter mentioned just before it.  2-back: responded 'yes' when the letter was the same as the letter two before it.  3-back: responded 'yes' when the letter was the same as the letter three before it. | Cognitively Demanding | M&Ms | Yes | No | Yes | 1 | No |
| Volz et al. (2021) (23) (Study 4) | Control*: eating while holding a one-digit number in memory.  Experimental tasks presented participants with food while completing the N-back task. All responding was made verbally.  1-back: responded 'yes' whenever the current letter was the same as the letter mentioned just before it.  2-back: responded 'yes' when the letter was the same as the letter two before it.  3-back: responses 'yes' when the letter was the same as the letter three before it. | Physically Demanding | M&Ms | Yes | No | Yes | 1 | No |
| Volz et al. (2021) (23) (Study 5) | Control*: eating while holding a one-digit number in memory.  Experimental tasks presented participants with food while completing the N-back task. All responding was made verbally.  1-back: responded 'yes' whenever the current letter was the same as the letter mentioned just before it.  2-back: responded 'yes' when the letter was the same as the letter two before it.    3-back: responded 'yes' when the letter was the same as the letter three before it. | Physically Demanding | M&Ms | Yes | No | Yes | 1 | No |
| Volz et al. (2021) (23) (Study 6) | Control*: eating while holding a one-digit number in memory.  Experimental tasks presented participants with food while completing the N-back task. All responding was made verbally.  1-back: responded 'yes' whenever the current letter was the same as the letter mentioned just before it.  2-back: responded 'yes' when the letter was the same as the letter two before it.  3-back: responded 'yes' when the letter was the same as the letter three before it.  4-back: responded 'yes' when the letter was the same as the letter four before it. | Physically Demanding | M&Ms | Yes | No | Yes | 1 | No |
| Volz et al. (2021) (23) (Study 7) | Control*: eating while holding a one-digit number in memory.  Experimental tasks presented participants with food while completing the N-back task. All responding was made using a foot pedal.  1-back: responded 'yes' whenever the current letter was the same as the letter mentioned just before it.  2-back: responded 'yes' when the letter was the same as the letter two before it.  3-back: responded 'yes' when the letter was the same as the letter three before it. | Cognitively Demanding | M&Ms | Yes | No | Yes | 1 | No |
| Mathiesen et al. (2022) (24) | Control*: eating while no sound played whilst the participant was in the restaurant.  Cafeteria: eating while listening to sound recordings from restaurant/cafeteria environments.  Slow: eating while listening to pre-recorded jazz instruments (at 65 bpm).  Fast: eating while listening to pre-recorded jazz instruments (at 160 bpm). | Passive | Iceberg lettuce, arugula, chicken breast, bread croutons, cherry tomatoes, slice of cucumber, dressing, slice of bread, 10g packet of margarine spread. | Yes | No | Yes | 2 | Yes |
| Francis et al. (2017) (25) | Control*: eating quietly without watching a TV program.  TV: eating while watching a TV program. | Passive | Pringles, mars pods, cheese bites, grapes, M&Ms, roasted almonds | No | No | Yes | 6 | No |
| Braude and Stevenson (2014) (26) | Control*: eating quietly without watching a TV program.  TV: eating while watching a TV program. | Passive | Maltesers, skittles, almonds, salted potato chips | No | No | Yes | 4 bowls in one condition, 1 bowl in another condition | No |
| Çetin et al. (2023) (27) | Control*: eating while no music was playing.  Classical music 60 dB: eating while classical music was played at 60 dB.  Classical music 80 dB: eating while classical music was played at 80 dB.  Rock music 60 dB: eating while rock music was played at 60 dB.  Rock music 80 dB: eating while rock music was played at 80 dB. | Passive | Pasta with Napolitano sauce mixed together | No | No | Yes | 1 | Yes |
| Kaiser et al. (2016) (28) | Control*: eating in silence.  Background loudspeaker: eating with instrumental background music via loudspeakers.    Background headphones: eating with instrumental background music via headphones.  English vocal music: eating while listening to English pop songs.  German vocal music: eating while listening to German pop songs. | Passive | Pork schnitzel, Swabian pasta, broccoli, carrots, cauliflower and cream sauce | Unclear | No | Yes | 1 plate | Yes |
| Mamalaki et al. (2017) (29) | Control*: eating with another participant, but with no music.  60 dB: eating with another participant, whilst listening to music at 60 dB.  90 dB: eating with another participant, whilst listening to music at 90 dB | Passive | Pasta with butter, tomato sauce and grated cheese | No | No | Unclear (no cover story given) | 1 | Yes |
| Rosenthal and Raynor (2016) (30) | Control*: eating while sat quietly and engaging in no other activities.  TV: eating while watching a TV show. | Passive | Macaroni and cheese, salad, salad dressing | No | No | Unclear (no cover story given) | 2 | Yes |
| Hussain et al. (2021) (31) | Control*: eating without listening to any music.  Classical music: eating while listening to classical music.  Popular music: eating while listening to popular music. | Passive | Walkers salted crisps, galaxy chocolate minstrels, Oreo cookies | Yes | No | Yes | 3 | No |
| Ward and Mann (32) (Study 1) | Low load*: eating while responding to a reaction time task using a foot button.  High load: eating while watching a series of art slides which they were asked to memorise and respond to a reaction time task during the slide show, using a foot button. | Cognitively Demanding | Doritos, M&Ms, chocolate chip cookies | Yes | No | Yes | 3 | No |
| Ward and Mann (32) (Study 2) | Low load*: eating while responding to a reaction time task using a foot button.  High load*: eating while watching a series of art slides which they were asked to memorise and respond to a reaction time task during the slide show, using a foot button. | Cognitively Demanding | Doritos, M&Ms, chocolate chip cookies | Yes | No | Yes | 3 | No |
| Lattimore and Maxwell (2004) (33) | Low load*: eating while completing either an ego-threat Stroop or a colour-name Stroop.  High load: eating while completing either an ego-threat Stroop (which required memorisation) or a colour-name Stroop (which required memorisation). | Physically Demanding. | Pringles, chocolate biscuits, buttons, flake, dried fruit dates, apricots, Florida mix | Yes | No | Yes | 1 | No |
| Shin (2024) (34) | Control*: eating without distraction.  Radio: eating while listening to a radio at 45 dB.  TV: eating while watching television.  Smartphone: eating while watching television and while having use of smartphone | Radio and TV = Passive.  Smartphone = Physically Demanding | Curry rice | Yes | No | Unclear (no cover story given) | 1 | Yes |
| Mann and Ward (2004) (35) | Low load*: eating while memorising a one-digit number.  High load: eating while memorising a nine-digit number. | Cognitively Demanding | Milkshake | Yes | No | Yes | 1 | No |

| Table S3. Continuation of concurrent intake studies. | | | | | | | | |
| --- | --- | --- | --- | --- | --- | --- | --- | --- |
| **Author and Year** | **Participant age** | **Participant gender/sex** | **Summarised information on body weight** | **Summarised information on disinhibition scores** | **Summarised information on restrained scores** | **Current dieting status (% currently dieting)** | **Liking scores of test foods** | **Energy intake** |
| Bellisle and Dalix (2001) (4) | Mean = 35.1, SD = 9 | 100% Female | Mean BMI = 21.3, SD = 1.9 | Measure: Three Factor Eating Questionnaire and Dutch Eating Behaviour Questionnaire.  Overall sample mean = 7.0, SD = 3.8 (TFEQ).  No split between high and low disinhibition scores used | Measure: Three Factor Eating Questionnaire and Dutch Eating Behaviour Questionnaire.  Overall sample mean = 8.2, SD = 4.5 (TFEQ).  No split between high and low restraint scores used | Not reported | Measured but not reported | Units: kilojoules  Control condition: mean = 1998, SD = 92.  Distraction condition: mean = 2299, SD = 92 |
| Bellisle, Dalix, and Slama (2004) (5) | Mean = 29.9, SD = 1.4 | 100% Female | Mean BMI = 22.3, SD = 0.2 | Measure: Three Factor Eating Questionnaire and Dutch Eating Behaviour Questionnaire.  Overall sample mean = 7.5, SD = 0.5 (TFEQ).  No split between high and low disinhibition scores used | Measure: Three Factor Eating Questionnaire and Dutch Eating Behaviour Questionnaire.  Overall sample mean = 7.6, SD = 0.6 (TFEQ).  No split between high and low restraint scores used | Not reported | Measured but not reported | Units: kilocalories  Control condition: mean = 419, SEM = 20.  Television: mean = 484, SEM = 20.  Audio recording: mean = 489, SEM = 20. |
| Bellisle et al. (2009) (6) | Low restraint group: mean = 26.4, SD = 6.71.  High restraint group: mean = 25.9, SD = 4.02.  Total sample mean = 26.15 | 100% Female | Low restraint group: mean BMI = 21.5, SD = 1.79.  High restraint group: mean BMI = 22.4, SD = 2.24.  Total sample mean = 21.95 | Measure: Three Factor Eating Questionnaire.  Type of split: None.  Low restraint: mean = 5.6, SD = 2.68  High restraint: mean = 8.4, SD = 4.02.  Total sample mean = 7 | Measure: Three Factor Eating Questionnaire.  Type of split: Low restraint group had a restraint score of 5 or lower, high restraint group had a restraint score of over 10.  Low restraint: mean = 4.00, SD = 2.24.  High restraint: mean = 11.50, SD = 3.58.  Total sample mean = 7.75 | Not reported | 100 VAS scale - Mean palatability across whole sample = 65.3 | Units: kilojoules  Control low restraint: mean = 2635, SD = 818.4  Control high restraint: mean = 2636, SD = 711.07  Television low restraint: mean = 2749, SD = 849.71  Television high restraint: mean = 2564, SD = 661.88  Auditory recording low restraint: mean = 2744, SD = 912.32    Auditory recording high restraint: mean = 2500, SD = 688.71 |
| Blass et al. (2006) (7) | Not reported | Male = 5, Female = 15 | Macaroni and cheese condition: mean BMI = 22.71, SD = 4.02.  Pizza condition: mean BMI = 26.35, SD = 6.66.  Mean across both conditions = 24.53 | Measure: Three Factor Eating Questionnaire and Dutch Eating Behaviour Questionnaire.  No data reported - only total TFEQ score | Measure: Three Factor Eating Questionnaire and Dutch Eating Behaviour Questionnaire.  No data reported - only total TFEQ score | Not reported | Not reported | Units: kilocalories  Control (macaroni and cheese): mean = 342.8, SD = 197.99  Television (macaroni and cheese): mean = 586.21, SD = 366.92  Control (pizza): mean = 715.7, SD = 133.99  Television (pizza): mean = 959.14, SD = 232.96 |
| Boon et al. (1997) (8) (Study 1) | Restrained group: mean age = 21.1, SD = 1.5. Unrestrained group: mean age = 21.6, SD = 2.1 | 100% Female | Restrained group: mean BMI = 23.1, SD = 2.0.  Unrestrained group: mean age = 21.6, SD = 2.1 | Not measured | Measure: Restraint scale.  Type of split: Median Split.  Restrained participants, mean = 14.9, SD = 3.4,  Unrestrained participants, mean = 7.8, SD = 1.8 | Not reported | Not reported | Units: grams  Control (restrained eaters): mean = 218.3, SD = 111.3  Control (unrestrained eaters): mean = 188.3, SD = 98.5  Distraction (restrained eaters): mean = 249.3, SD = 82.5  Distraction (unrestrained eaters): mean = 244.6, SD = 126.3 |
| Boon et al. (1997) (8) (Study 2) | Not reported | Not reported | Not reported | Not measured | Measure: Restraint scale.  Type of split: Not stated.  Restrained participants, mean = 17.7, SD = 3.9,  Unrestrained participants, mean = 6.9, SD = 2.4 | Not reported | Not reported | Units: grams  Control (restrained eaters): mean = 143.0, SD = 81.7  Control (unrestrained eaters): mean = 138.9, SD = 86.9  Distraction (restrained eaters): mean = 166.8, SD = 86.2  Distraction (unrestrained eaters): mean = 158.7, SD = 93.1 |
| Boon et al. (2002) (9) | Restrained group: mean age = 21.1, SD = 2.4.  Unrestrained group: mean age = 21.2, SD = 2.3 | 100% Female | Restrained group: mean BMI = 23.3, SD = 2.5.  Unrestrained group: mean age = 20.6, SD = 1.8 | Not measured | Measure: Restraint scale.  Type of split: median split.  Restrained participants, mean = 15.4, SD = 3.0,  Unrestrained participants, mean = 7.3, SD = 2.4 | Not reported | Not reported | Units: grams  Control (High calorie + restrained eaters) mean = 154.6, SD = 98.9  Control (High calorie + unrestrained eaters): mean = 176.0, SD = 77.5  Control (Low calorie + restrained eaters): mean = 186.0, SD = 75.3  Control (Low calorie + unrestrained eaters): mean = 175.1, SD = 97.0  Distraction (High calorie + restrained eaters): mean = 274.6, SD = 96.9  Distraction (High calorie + unrestrained eaters): mean = 192.5, SD = 76.5  Distraction (Low calorie + restrained eaters): mean = 234.9, SD = 95.2  Distraction (Low calorie + unrestrained eaters): mean = 261.0, SD = 78.2 |
| Hetherington et al. (2006) (10) | Mean = 28.3, SEM = 1.7 | 21 males, 16 females | Mean = 23.87, SEM = 0.8 | Measure: Three Factor Eating Questionnaire and Dutch Eating Behaviour Questionnaire. No data reported | Measure: Three Factor Eating Questionnaire and Dutch Eating Behaviour Questionnaire.  Overall sample mean (DEBQ restraint) = 2.24, SEM = 0.13.  Overall sample mean (TFEQ restraint) mean = 9.06, SEM = 0.95. | Not reported | Not reported | Units: kilojoules  Control: mean = 3861, SD = 1216.55    Television: mean = 4350, SD = 1532.86 |
| Long et al. (2011) (11) | Mean = 21.2, SE = 0.7 | 100% Female | Mean = 23.8, SE = 0.64 | Not measured | Not measured | Not reported | Not reported | Units: grams  Control: mean = 425.8, SD = 177.71  Distraction: mean grams = 513.4, SD = 195.38 |
| Martin et al. (2009) (12) | Mean = 31.9 | 22 males, 26 females | Mean = 25.8 | Measure: Three Factor Eating Questionnaire.  Mean score = 4.4, SEM = 0.5 | Measure: Three Factor Eating Questionnaire.  Mean score = 7.3, SEM = 0.7 | Not reported | Not reported | Units: kilocalories  Control: mean = 1053, SD = 911.2  Reading: mean = 995, SD = 1055.1  Television (no adverts): mean = 1028, SD = 1007.2 |
| Kononova et al. (2018) (13) | Mean = 21.8, SD = 1.64 | Females 76% | Mean = 23.29, SD = 5.05 | Not measured | Not measured | Not reported | Not reported (but measured) | Units: grams  Control (TV only): mean = 3.85, SD = 0.59  TV+texting: mean = 3.54, SD = 1.05  TV+texting+online reading: mean = 3.31, SD = 1.28  TV+texting+online shopping: mean = 3.58, SD = 0.94 |
| Ding et al. (2019) (14) | Mean = 25.79, SD = 4.87 | Male = 14, female = 29 | Mean BMI = 21.75, SD = 2.75 | Not measured | DEBQ: mean = 2.46, SD = 0.77 | Not measured | Measured food pleasantness. Control: mean = 4.47, SD = 1.29.  Distraction: mean = 3.69, SD = 1.27 | Units: kilojoules  Control: mean = 3105.17, SD = 1101.32  Distraction: mean = 3083.87, SD = 1162.03 |
| Arch et al. (15) (Study 3) | Mean = 20.78, SD = 3.87 | Males = 59, Females = 43 | Not reported | Not reported | Not reported | Not reported | Not reported | Units: kilocalories  Calories. Mindfulness: mean = 196.68, SD = 135.24,  Distraction: mean = 251.20, SD = 142.28.  Control: mean = 259.65, SD = 159.23 |
| Lyons et al. (2012) (16) | Mean: 24.07, SD = 4.43 | Males = 60, Females = 60 | Mean: 24.41, SD = 4.14 | Not measured | Not measured | Not reported | Not reported | Units: kilocalories  Television: mean = 716, SD = 407.  Video games: mean = 747, SD = 540.  Motion-controlled video games: mean = 553, SD = 498 |
| Stämpfli & Brunner (2016) (17) | Mean = 46.35, SD = 14.20 | Females = 73.44% | Not reported | Not measured | Not measured | Not reported | Measured but not reported | Units: grams  Low cognitive load (White screensaver): mean = 13.88, SD = 9.94  High cognitive load (White screensaver): mean = 15.24, SD = 8.5  Low cognitive load (Giacometti screensaver): mean = 12.01, SD = 6.85  High cognitive load (Giacometti screensaver): mean = 10.96, SD = 5.12 |
| Brunner (2013) (18) (Study 4) | Mean = 28.0, SD = 7.96 | Female = 56, male = 41 | Mean = 21.8, SD = 2.82 | Not measured | Not measured | Not reported | Not measured | Units: grams  Low cognitive load (Fingers): mean = 9.22, SD = 5.17  High load (Fingers): mean = 8.27, SD = 5.39  Low cognitive load (Tongs): mean = 6.96, SD = 5.10  High load (Tongs): mean = 5.74, SD = 3.97 |
| da Mata Gonçalves et al. (2019) (19) | Mean (BMI < 25) = 20.2, SD = 2.0.  Mean (BMI ≥ 25) = 21.9, SD = 3.0. | Females = 36, Males = 26 | BMI < 25: mean = 20.7, SD = 1.9. BMI ≥ 25: mean = 27.7, SD = 2.2 | Not measured | Not measured | Not reported (but likely 0% due to exclusion criteria) | Not measured | Units: kilocalories  Control: mean = 535, SD = 164  Smartphone: mean = 591, SD = 203  Reading: mean = 622, SD = 226 |
| Ogden et al. (2013) (20) | Driving: mean = 22.38, SD = 4.93,  Television: mean = 22.9, SD = 5.45.  Social: mean = 21.95, SD = 5.57.  Alone: mean = 21.9, SD = 5.14 | All females | Driving: mean = 21.26, SD = 2.39.  Television: mean = 22.69, SD = 5.16.  Social: mean = 22.24, SD = 5.19.  Alone: mean = 22.56, SD = 4.25 | Not reported | Measured using the DEBQ.  Driving: mean = 2.67, SD = 1.0,  Television: mean = 2.43 SD = 0.94.  Social: mean = 2.45, SD = 0.99.  Alone: mean = 2.45, SD = 0.78 | Not reported | Not reported | Units: grams  Driving: mean = 14.02, SD = 10.34  Television: mean = 28.61, SD = 24.44  Social: mean = 14.16, SD = 12.33  Alone: mean = 18.21, SD = 20.9 |
| van der Wal et al. (2013) (21) (Study 3) | Mean = 20.2, SD = 1.4 | Female = 69, Male = 50 | Mean = 23.0, SD = 3.8 | Not measured | Not measured | Not reported, (but likely 0% due to exclusion criteria) | Not measured | Units: Percentage of food consumed  Low load salt-free butter: mean = 44.95, SD = 14.64.  High load salt-free butter: mean = 46.95, SD = 16.62.  Low load salty butter: mean = 42.91, SD = 12.45.  High load salty butter: mean = 58.93, SD = 22.59 |
| Liguori et al. (2020) (22) | Mean = 20.2, SD = 1.4 | Female = 69, Male = 50 | Mean = 23.0, SD = 3.8 | Not measured | Not measured | Not reported, (but likely 0% due to exclusion criteria) | Not measured | Units: grams  Control: mean = 128, SD = 49  Distraction: mean = 115, SD = 60 |
| Volz et al. (2021) (23) (Study 1) | Not reported | Female = 187 | Not reported | Not reported | Not reported | Not measured | Not reported | Units: grams  Control: mean = 25.28, SD = 17.00  0-back: mean = 34.69, SD = 17.90  1-back: mean = 28.15, SD = 16.74  2-back: mean = 30.46, SD = 24.75  3-back: mean = 28.67, SD = 17.28 |
| Volz et al. (2021) (23) (Study 2) | Not reported | Female = 84 | Not measured | Not reported | Not reported | Not measured | Not reported | Units: grams  Control: mean = 36.96, SD = 18.31  1-back: mean = 60.00, SD = 29.08  2-back: mean = 37.05, SD = 23.65  3-back: mean = 50.90, SD = 31.83 |
| Volz et al. (2021) (23) (Study 3) | Mean = 22.29, SD = 7.2 | Female = 76, Male = 37, Other Gender Identity = 1 | Not measured | Not reported | Not reported | Not measured | Not reported | Units: grams  Control: mean = 22.67, SD = 15.17  1-back: mean = 36.41, SD = 15.90  2-back: mean = 40.38, SD = 30.03  3-back: mean = 46.14, SD = 45.49 |
| Volz et al. (2021) (23) (Study 4) | Mean = 22.84, SD = 7.14 | Female = 29, Male = 28 | Not measured | Not reported | Not reported | Not measured | Not reported | Units: grams  Control: mean = 7.89, SD = 6.59  1-back: mean = 7.75, SD = 7.16  2-back: mean = 6.54, SD = 5.04  3-back: mean = 5.32, SD = 4.50 |
| Volz et al. (2021) (23) (Study 5) | Mean = 19.79, SD = 2.56 | Female = 74, Male = 40, Other Gender Identity = 1 | Not measured | Not reported | Not reported | Not measured | Not reported | Units: grams  Control: mean = 7.33, SD = 6.42  1-back: mean = 7.05, SD = 6.21  2-back: mean = 7.17, SD = 7.05  3-back: mean = 5.45, SD = 5.97 |
| Volz et al. (2021) (23) (Study 6) | Mean = 23.24, SD = 8.00 | Female = 52, Male = 22 | Not measured | Not reported | Not reported | Not measured | Not reported | Units: grams  Control: mean = 10.35, SD = 7.16  1-back: mean = 7.73, SD = 6.79  2-back: mean = 6.70, SD = 5.35  3-back: mean = 6.22, SD = 5.55  4-back: mean = 5.90, SD = 4.61. |
| Volz et al. (2021) (23) (Study 7) | Mean = 19.58, SD = 1.98 | Female = 41, Male = 23 | Not measured | Not reported | Not reported | Not measured | Not reported | Units: grams  Control: mean = 17.19, SD = 9.37  1-back: mean = 14.08, SD = 9.66  2-back: mean = 12.89, SD = 10.03  3-back: mean = 10.58, SD = 9.14 |
| Mathiesen et al. (2022) (24) | Mean = 39, SD = 12.5 | Female = 180, Male = 65, Not disclosed = 3 | Not measured | Not measured | Not measured | Not measured | Not reported | Units: grams  Control: mean = 586.02, SD = 101.92  Cafeteria: mean = 598.45, SD = 102.68  Slow: mean = 604.04, SD = 108.5  Fast: mean = 604.67, SD = 92.91 |
| Francis et al. (2017) (25) | Mean = 19.7, SD = 2.9. | Female = 95, male = 58 | Mean = 22.4, SD = 3.1 | Measured using TFEQ  TV (Female): mean = 7.4, SD = 3.3.  TV (Male): mean = 5.6, SD = 2.8.  Control (Female): mean = 7.1, SD = 2.7.  Control (Male): mean = 6.5, SD = 3.5 | Measured using TFEQ  TV (Female): mean = 8.1, SD = 4.8.  TV (Male): mean = 7.7, SD = 5.0.  Control (Female): mean = 7.9, SD = 5.9.  Control (Male): mean = 7.3, SD = 5.4 | Not measured | Not reported | Units: kilojoules  Control (Female): mean = 1778.93, SD = 1233.5  Control (Male): mean = 3017.80, SD = 1280.42  TV (Female): mean = 2016.32, SD = 1118.84  TV (Male): mean = 2172.11, SD = 1079.34 |
| Braude and Stevenson (2014) (26) | Mean = 19.6, SD = 2.2 | Female = 62 | Single food group: mean = 21.9, SD = 2.0.  Variety food group: mean = 22.3, SD = 2.7 | Measured using TFEQ  Single food group: mean = 7.8, SD = 2.8.  Variety food group: mean = 7.0, SD = 3.2 | Measured using TFEQ  Single food group: mean = 8.8, SD = 5.9.  Variety food group: mean = 8.9, SD = 5.4 | Not measured | Measured but not reported | Units: kilojoules  Control (Single food): mean = 514.8, SD = 424.2  Control (Variety food): mean = 646.2, SD = 462.8  TV (Single food): mean = 687.2, SD = 466.4  TV (Variety food): mean = 727.3, SD = 413.2 |
| Çetin et al. (2023) (27) | Mean = 21.6, SD = 1.50 | Female = 35 | Mean = 21.7, SD = 2.04 | Not measured | Not measured | Dieters were excluded from the study | Not measured | Units: kilocalories  Control: mean = 966.23, SD = 295.03  Classical music 60 dB: mean = 1047.27, SD = 313.49  Classical music 80 dB: mean = 1072.21, SD = 331.89  Rock music 60 dB: mean = 1069.09, SD = 313.49  Rock music 80 dB: mean = 1016.1, SD = 313.49 |
| Kaiser et al. (2016) (28) | Overall sample: mean = 23.03, SD = 2.53.  Control: mean = 22.90, SD = 2.78.  Background loudspeaker: mean = 23.38, SD = 2.18.  Background headphones: mean = 22.19, SD = 2.12.  English vocal music: mean = 23.24, SD = 2.90.  German vocal music: mean = 23.52, SD = 2.53. | Male = 73 Female = 74 | Control: mean = 22.37, SD = 3.37.  Background loudspeaker: mean = 23.27, SD = 3.01.  Background headphones: mean = 22.71, SD = 3.20.  English vocal music: mean = 23.96, SD = 2.81.  German vocal music: mean = 22.87, SD = 4.89 | Not measured | Measured but not reported | Not measured | Meal palatability rating (visual analogue scale out of 40).  Control: mean = 24.79, SD = 5.30.  Background loudspeaker: mean = 24.51, SD = 5.25.  Background headphones: mean = 24.74, SD = 5.65.  English vocal music: mean = 25.41, SD = 6.21.  German vocal music: mean = 24.05, SD = 6.21. | Units: grams  Control: mean = 642.31, SD = 181.03  Background loudspeaker: mean = 698.59, SD = 216.35  Background headphones: mean = 727.32, SD = 260.14  English music: mean = 700.52, SD = 222.76  German music: mean = 613.41, SD = 166.17 |
| Mamalaki et al. (2017) (29) | Median = 21 | Male = 26, no female participants | Median = 23.7 | Not measured | Measured but not reported | Not measured | Not measured | Units: kilocalories  Control: mean = 1079, SD = 330.  60 dB music: mean = 1064, SD = 324  90 dB music: mean = 1136, SD = 311 |
| Rosenthal and Raynor (2017) (30) | Mean = 22.3, SD = 3.7 | Female = 17, Male = 3 | BMI: mean = 21.6, SD = 2.3 | Not measured | Measured using TFEQ  Note: order refers to the order in which participants completed all conditions.  Order 1: mean = 8.6, SD = 2.1.  Order 2: mean = 6.0, SD = 3.9.  Order 3: mean = 6.8, SD = 4.3.  Order 4: mean = 4.8, SD = 1.6 | None (part of the exclusion criteria) | Initial liking of foods (scored out of 100)  Macaroni and cheese (order 1): Mean = 78.6, SD = 10.9.  Macaroni and cheese (order 2): mean = 73.0, SD = 15.8.  Macaroni and cheese (order 3): mean = 75.2, SD = 16.5.  Macaroni and cheese (Order 4): mean = 83.0, SD = 14.7.  Salad with dressing (order 1): mean = 73.6, SD = 16.9.  Salad with dressing (order 2): mean = 84.0, SD = 8.1.  Salad with dressing (order 3): mean = 78.6, SD = 19.0.  Salad with dressing (order 4): mean = 78.2, SD = 10.1 | Units: kilocalories  Control (Small portion size): mean = 713.21, SD = 169.81  Control (Large portion size): mean = 901.89, SD = 264.15  TV (Small portion size): mean = 766.04, SD = 203.77  TV (Large portion size): mean = 916.98, SD = 279.25 |
| Hussain et al. (2021) (31) | Mean = 26.18, SD = 13.02 | Female = 76, male = 21, Not specified = 3 | Mean = 21.72, SD = 10.77 | Measured using TFEQ.  Classical music: mean = 21.45, SD = 6.77.  Popular music: mean = 20.73, SD = 6.58.  No music: mean = 22.12, SD = 4.96 | Measured using TFEQ.  Classical music: mean = 13.06, SD = 4.86.  Popular music: mean = 12.70, SD = 4.23.  No music: mean = 12.44, SD = 3.70 | Not measured | Not measured | Units: kilocalories  Classical music: mean = 183.41, SD = 97.54  Popular music: mean = 173.68, SD = 125.72  Control (no music): mean = 218.86, SD = 143.23 |
| Ward and Mann (2000) (32) (Study 1) | Not reported | 60 females | Not reported | Not measured | Measured using the revised restraint scale.  Restrained sample: mean = 21.27, SD = 3.73.  Unrestrained sample: mean = 9.17, SD = 3.53. | Not measured | Not measured | Units: grams  Low load (Unrestrained eaters): mean = 59.67, SD = 26.50  High load (Unrestrained eaters): mean = 43.60, SD = 24.04  Low load (Restrained eaters): mean = 37.98, SD = 19.53  High load (Restrained eaters): mean = 52.53, SD = 25.46 |
| Ward and Mann (2000) (32) (Study 2) | Not reported | 29 females | Not reported | Not measured | Measured using the revised restraint scale.  Mean = 21.67, SD = 3.64 | Not measured | Not measured | Units: grams  Low load: mean = 46.70, SD = 29.00  High load: mean = 71.60, SD = 35.30 |
| Lattimore and Maxwell (2004) (33) | Mean = 23.6, SD = 7.7 | Female = 119 | BMI: mean = 23.4, SD = 3.6 | Not measured | Measured but not reported | Not measured | Not measured | Units: grams  Ego threat Stroop (Restrained eaters): mean = 83.1, SD = 36.29  Ego threat Stroop (Unrestrained eaters): mean = 92.1, SD = 24  Colour Stroop (Restrained eaters): mean = 91.8, SD = 30.12  Colour Stroop (Unrestrained eaters): mean = 89.0, SD = 21.13  Ego threat Stroop memorisation (Restrained eaters): mean = 114.6, SD = 51.2  Ego threat Stroop memorisation (Unrestrained eaters): mean = 73.8, SD = 34.63  Colour Stroop memorisation (Restrained eaters): mean = 75.3, SD = 20.14  Colour Stroop memorisation (Unrestrained eaters): mean = 77.8, SD = 28.81 |
| Shin (2024) (34) | Mean = 24.96, SD = 1.36 | Females = 13, males = 10 | Mean = 23.08, SD = 0.94 | Not measured | Not measured | Not measured | Not measured | Units: grams  Control: mean = 536.65, SD = 217.87  Radio: mean = 558.13, SD = 196.87  TV: mean = 560.35, SD = 223.49  Smartphone: mean = 642.52, SD = 186.85 |
| Mann and Ward (2004) (35) | Not reported | Female = 101 | Not reported | Measured but not reported | Measured using the revised restraint scale.  Total sample of 102 (which includes an excluded participant): mean = 20.2, SD = 3.6 | Not measured | Not measured | Units: grams  Low load (Diet-salient): mean = 164.43, SD = 89.63  Low load (Milkshake-salient): mean = 171.51, SD = 89.64  High load (Diet-salient): mean = 109.08, SD = 90.48  High load (Milkshake-salient): mean = 198.05, SD = 90.15 |

## Later intake studies

| Table S4. Extraction of later intake studies | | | | | | | | | |
| --- | --- | --- | --- | --- | --- | --- | --- | --- | --- |
| **Author and Year** | **Country** | **Study setting (laboratory or real-world)** | **Details of the sample group (e.g., university students)** | **Sample size** | **N per condition** | **Study design (within-subjects, between-subjects, mixed design)** | **Exclusion criteria** | **Distraction manipulation (stating details of the distraction task for experimental condition(s) and details of the control condition).** | **Test foods. Reporting the test foods used for the fixed meal and subsequent eating episode for later energy intake studies.** |
| Higgs and Woodward (2009) (36) | UK | Laboratory | Female university students | 16 | 16 | Within-subjects | Not reported | Experimental condition: Consumed fixed laboratory lunch whilst watching television.  Control condition: Consumed fixed laboratory lunch without watching television. | Fixed meal: ham sandwiches and ready salted crisps.  Subsequent eating episode: Cadbury's milk chocolate fingers, Maryland chocolate chip cookies, McVitie’s digestives (40g of each). |
| Mittal et al. (2011) (37) (Study 1) | Australia | Laboratory | Female university students | 32 | 16 in each condition | Between-subjects | Participant had to: be female gendered, have English as their primary language, have no history of diabetes nor any eating related condition, not have dieted in the preceding 3 months and have a BMI between 18 and 25. | Experimental condition: Consumed fixed laboratory snack whilst watching television.  Control: Consumed fixed laboratory snack without watching television. | Fixed meal: Chocolate balls (Arnott’s), Potato crisps (Pringles), Coke/Orange Juice.  Subsequent eating episode: Sandwiches (Woolworths), biscuits (Woolworths), Crackers & Dip (Kraft). |
| Mittal et al. (2011) (37) (Study 2) | Australia | Laboratory | Female university students | 84 | 21 in each condition | Between-subjects | Not reported | Funny TV condition: consumed fixed laboratory snack whilst watching funny television program.  Sad TV condition: consumed fixed laboratory snack whilst watching sad television program  Boring TV condition: consumed fixed laboratory snack whilst watching boring television program.  Control condition: Consumed fixed laboratory snack in absence of television. | Fixed meal: Chocolate balls (Arnott’s), Potato crisps (Pringles), Coke/Orange Juice.  Subsequent eating episode: Sandwiches (Woolworths), biscuits (Woolworths), Crackers & Dip (Kraft). |
| Oldham-Cooper et al. (2010) (38) | UK | Laboratory | Male and female participants | 44 | 22 in each condition | Between-subjects | Exclusion criteria: Vegetarians, vegans, those with specific dietary requirements | Distraction condition: ate fixed lunch while playing solitaire.  Control condition: ate lunch, attending to sensory characteristics of the foods | Fixed meal: cheese twists, ham sandwich, carrot batons, mini-Cornish pasty, cheese sandwich, sausage rolls, cherry tomatoes, scotch egg, potato chip snack.  Subsequent eating episode: chocolate chip cookies, milk chocolate digestive biscuits, sweet oat-based biscuits |
| Higgs et al. (2015) (39) (Study 1) | UK | Laboratory | Female university students | 39 | 13 in each condition | Between-subjects | Recruited females only. Other eligibility criteria not stated | Control: participants consumed lunch with no game as a distraction.  Low distraction: participants played a computer game without an incentive.  High distraction: participants played a computer game with a monetary incentive. | Fixed meal: salt and vinegar crisps, cheese and tomato sandwich, mini sausage roll, cherry tomatoes, ham sandwich, ready salted crisps, mini Cornish pasty, carrot batons.  Subsequent eating episode: chocolate chip cookies, custard creams, nice biscuits. |
| Higgs et al. (2015) (39) (Study 2) | UK | Laboratory | Female university students | 63 | 21 in each condition | Between-subjects | Recruited females only. Other eligibility criteria not stated | Control: consumed lunch whilst watching no TV.  Food-related TV: consumed lunch whilst watching a food-related TV clip.  Non-food-related TV: Consumed lunch whilst watching a non-food-related TV clip | Fixed meal: 300g tin of Heinz cream of tomato soup, one slice of bread.  Subsequent eating episode: McVitie’s chocolate digestives, Cadbury's milk chocolate fingers, Maryland chocolate chip cookies |
| Morris et al. (2020) (40) | UK | Laboratory | Females | 120 | 30 in each group | Between-subjects | Participants had to be: female, have normal or corrected to normal vision, were native speakers (or fluent at speaking and reading English as a native speaker) | Low distraction: consumed fixed meal whilst completing a low perceptual load task.  High distraction: consumed fixed meal whilst completing a high perceptual load task | Fixed meal (experimentally manipulated): a fruit smoothie containing maltodextrin (high energy thick texture drink), or a fruit smoothie not containing maltodextrin (low energy thin texture drink).  Subsequent eating episode: ready salted crisps, cool tortilla chips, mini poppadums. |
| Whitelock et al. (2018) (41) (Study 1) | UK | Laboratory | Adults (no specific group) | 108 | Normal control = 37  Headphone control = 37  Focused attention = 34 | Between-subjects | Participants had to be: aged 18-60, have a self-reported BMI between 22.5 and 32.5, be fluent speakers of English, be regular breakfast eaters, not be taking mediation that affects appetite, have no food allergies or history of disordered eating. | Headphone control: participants listened to a description of the migration and breeding pattern of cuckoo birds whilst eating a fixed lunchtime meal.  Normal control: participants consumed a fixed lunch meal without headphones | Fixed meal: cooked pasta with tomato sauce.  Subsequent eating episode: Maryland cookies and Cadbury’s chocolate fingers. |
| van Meer et al. (2023) (42) | Netherlands | Laboratory | University students | 116 | Control = 58  Driving = 58 | Between-subjects | Exclusion criteria: Smoking or having allergies, being outside of the age range 18-30 | Control: acted as passenger in a driving simulator. Driving: acted as driving in a driving simulator. | Fixed meal: potato chips.  Subsequent eating episode: potato chips |
| Duif et al. (2020) (43) | Netherlands | Laboratory | Adults (no specific group) | 41 | 41 in each condition | Within-subjects | Eligibility criteria: BMI between 18.5 and 30.0, aged 18-35, right-handed.  Exclusion criteria: currently pregnant, MRI-incompatibility; diabetes mellitus, history of hepatic, cardiac, respiratory, renal, cerebrovascular, endocrine, metabolic or pulmonary diseases; uncontrolled hypertension; neurological, psychiatric, or eating disorders; current strict dieting; restrained eating score ≥ 4.00 for females and ≥ 3.60 for males on the Dutch Eating Behaviour Questionnaire | Low attention load: participants completed a low-load categorical visual detection task whilst receiving a fixed amount of chocolate milk.  High attention load: participants completed a high-load categorical visual detection task whilst receiving a fixed amount of chocolate milk. | Fixed meal: chocolate milk.  Subsequent eating episode: M&Ms |

| **Author and Year** | **The duration of time between the distraction task and the later energy intake eating episode.** | **Participant age** | **Participant gender/sex** | **Summarised information on body weight** | **Summarised information on disinhibition** | **Summarised information on restrained scores** | **Current dieting status (% currently dieting)** | **Liking scores of test foods** | **Energy intake** | **Meal enjoyment/satisfaction rating.** |
| --- | --- | --- | --- | --- | --- | --- | --- | --- | --- | --- |
| Higgs and Woodward (2009) (36) | 150 minutes | Mean = 19, SD = 1 | 100% female | Mean BMI = 21.7, SD = 1.75 | Not reported | Measured using the DEBQ  Mean = 2.6, SD = 0.7 | Not reported | Not reported | Units: grams  Control: mean = 56.54, SD = 18.96.  TV: mean = 68.83, SD = 21.33. | Not reported |
| Mittal et al. (2011) (37) (Study 1) | 45 minutes | Experimental group: mean = 20.8, SD = 3.8.  Control group: mean = 20.3, SD = 3.9 | 100% female | Experimental group: mean = 21.5, SD = 1.6  . Control group: mean = 21.8, SD = 2.1.  Total sample mean BMI = 21.65. | Measured using TFEQ.  Experimental group: mean = 6.1, SD = 2.6.  Control group: mean = 6.3, SD = 2.0 | Measured using TFEQ.  Experimental group: mean = 6.9, SD = 4.5.  Control group: mean = 7.1, SD = 4.8 | Current dieters excluded | Not reported | Units: kilojoules  Control condition: mean = 1354.9, SD = 335.6.  Television condition: mean = 1584.6, SD = 516.4. | Not reported |
| Mittal et al. (2011) (37) (Study 2) | 45 minutes | Boring: mean = 22.8, SD = 4.1  Sad: mean = 20.6, SD = 3.2.  Funny: mean = 21.3, SD = 2.7.  Control: mean = 20.6, SD = 2.7. | 100% female | Boring: mean = 21.4, SD = 2.1  Sad: mean = 21.2, SD = 1.5  Funny: mean = 21.7, SD = 1.9  Control: mean = 21.0, SD = 1.8. | Measured using TFEQ  Boring: mean = 6.3, SD = 2.6  Sad: mean = 7.4, SD = 2.8  Funny: mean = 7.7, SD = 3.9.  Control: mean = 5.4, SD = 2.3 | Measured using TFEQ  Boring: mean = 5.6, SD = 3.7  Sad: mean = 7.6, SD = 4.2  Funny: mean = 8.8, SD = 4.5.  Control: mean = 6.4, SD = 5.3 | Not reported | Not reported | Units: kilojoules  Control: mean = 2147.9, SD = 527.2.  Boring: mean = 2507.0, SD = 438.2.    Sad: mean = 2842.0, SD = 452.4.  Funny: mean = 2637.6, SD = 540.3. | Not reported |
| Oldham-Cooper et al. (2010) (38) | 50 minutes | Distraction: mean = 28.1, SD = 17.2.  Control: mean = 26.3, SD = 15.0 | 50% female  50% male | Distraction: mean = 23.1, SD = 3.0.  Control: mean = 23.6, SD = 3.07 | Not reported | DEBQ Distraction: mean = 2.45, SD = 0.94.  No distraction: mean = 2.31, SD = 0.86 | Not reported | Liking for biscuits (scored out of 100):  Distraction: mean = 59.5, SD = 12.3.  No distraction: mean = 58.4, SD = 11.3 | Units: grams  Control: mean = 27.1, SD = 26.4.  Distraction: mean = 52.1, SD = 45.1. | Not reported |
| Higgs et al. (2015) (39) (Study 1) | 90 minutes | Mean = 20, SD = 1.7 | All female | Mean = 22, SD = 2.4 | Not measured | Mean = 2.7, SD = 0.9 | Not reported | Not measured | Units: grams  Control: mean = 21.4, SD = 17.29  High distraction: mean = 36.2, SD = 17.29  Low distraction: mean = 29.8, SD = 17.38 | Not measured |
| Higgs et al. (2015) (39) (Study 2) | 150 minutes | Mean = 19.7, SD = 3.5 | All female | Mean = 22.1, SD = 3.4 | Measured using TFEQ  Control: mean = 9, SD = 2.5.  Food TV: mean = 7.9, SD = 2.  TV: mean = 9, SD = 2.1 | Measured using the DEBQ  Control: mean = 2.6, SD = 1.0.  Food TV: mean = 2.5, SD = 1.0.  TV: mean = 2.5, SD = 1.0 | Not reported | Measured for fixed meal but not reported | Units: grams  Control condition: mean = 67.4, SD = 39.98.  TV condition: mean = 82.8, SD = 39.74.  Food-related TV: mean = 74.7, SD = 39.86 | Measured for fixed meal but not reported |
| Morris et al. (2020) (40) | 32.5 minutes | Mean = 20.58, SD = 2.53. | All female | Mean = 22.91, SD = 3.87 | Measured using TFEQ  mean = 7.12, SD = 2.92 | Measured using TFEQ  mean = 8.93, SD = 3.86 | Not reported | Pleasantness rating of fixed meal: mean = 61.68, SD = 21.92 | Units: kilocalories  Low load (low energy drink): mean = 164.81, SD = 71.26.  High load (low energy drink): mean = 129.22, SD = 70.84.  Low load (high energy drink): mean = 96.54, SD = 49.22.  High load (high energy drink): mean = 138.98, SD = 62.30 | Not measured |
| Whitelock et al. (2018) (41)(Study 1) | 180 minutes | Normal control: mean = 27.57, SD = 11.99.  Headphone control: mean = 29.16, SD = 10.63.  Focused attention: mean = 29.91 SD = 10.50 | Males = 51, Females = 57 | Normal control: mean = 26.02, SD = 3.21.  Headphone control: mean = 25.27, SD = 2.94.  Focused attention: mean = 25.97, SD = 3.98. | Not measured | TFEQ. Normal control: mean = 2.23, SD = 0.42.  Headphone control: mean = 2.45, SD = 0.50.  Focused attention: mean = 2.34, SD = 0.49 | Not measured | Scores on 100-point scale. Normal control: mean = 64.05, SD = 28.48.  Headphone control: mean = 56.59; SD = 19.34.  Focused attention: mean = 48.62, SD = 26.57 | Units: kilocalories  Control (males): mean = 470.43, SD = 243.71  Control (females): mean = 286.05, SD = 147.67  Headphone (males): mean = 412.69, SD = 214.56.  Headphone (females): mean = 300.96, SD = 153.55. | 100-point scale.  Normal control: mean = 67.46, SD = 25.83.  Headphone control: mean = 61.24, SD = 19.87.  Focused attention: mean = 52.00, SD = 25.47. |
| van Meer et al. (2023) (42) | 5 minutes | Mean = 22.30, SD = 4.98 | Males = 30, Females = 86 | Not measured | Not measured | Measured but not reported | Not measured | Tasty ratings of subsequent consumption of potato chips.  Driving: mean = 5.17, SD = 1.35.  Control: mean = 4.98, SD = 1.40 | Units: kilocalories  Control: mean = 72.9, SD = 24.7.  Driving: mean = 84.3, SD = 31.1 | Not measured |
| Duif et al. (2020) (43) | 45 minutes | Mean = 22.5, SD = 3.5 | Male = 10, Female = 31 | Mean = 21.9, SD = 1.89 | Not measured | Not measured | Not measured | 10-point visual analogue scale.  Low sweet drink: mean = 5.4, SE = 0.3.  High sweet drink: mean = 6.1, SE = 0.3.  Neutral solution: mean = 4.5, SE = 0.5 | Units: grams  Low load: mean = 65.6, SD = 37.78.  High load: mean = 68.1, SD = 43.54 | Not measured |

Risk of Bias indicators

Self-reported energy intake (real-world studies only). Studies were coded as ‘Yes’ or ‘No’ regarding whether self-reported data was used to calculate energy intake. Studies which used self-reported data were considered higher in risk of bias, due to error and biases associated with this form of measurement.

Eligibility criteria. Studies which did not include key criteria for appetite and eating behaviour studies were considered as having a higher risk of bias. These criteria were: currently taking medication known to affect appetite, currently dieting.

Inadequate information of methodology. Studies which presented an insufficient level of detail and information regarding the study methodology were considered as having higher risk of bias (e.g., information on foods used, form of distraction, control condition, assessment of energy intake, length of washout period for within-subject designs).

Random allocation. Studies which did not report that random allocation to experimental conditions had occurred (order of conditions for within-subjects studies randomised, allocation to different conditions for between-subjects studies randomised) were considered higher in risk of bias.

Demand characteristics. Studies which did not address the influence of participant awareness of study aims of behaviour were considered higher in risk of bias. Studies were deemed to have a higher risk of bias relating to demand characteristics if a study did any one (or more) of the following:

- Did not report measuring participant awareness of study aims.
- Reported > 50% of participants having an awareness of the study aims (awareness of study aims was defined as how the study has defined it).
- Reported a difference in study findings when removing participants who had correctly guessed the study aims.
- Did not attempt to mask the true aims of the study (i.e., the researcher had not withheld this information and/or had not used a cover story).

Small number of participants per condition. Studies which used a sample size of N<12 for within-subject studies and N<20 per condition for between-subject designs were considered higher in risk of bias.

Pre-registration. Studies which did not include a pre-registered protocol (i.e., details of the methodology and analysis plan) were considered higher in risk of bias.

Abstaining from eating. Studies which did not ask participants to report whether or not they have abstained from eating prior to the start of the experiment (concurrent energy intake studies) or prior to the subsequent ad libitum eating/drink episode (later energy intake studies) were considered higher in risk of bias.

## Bias score breakdown – concurrent intake studies

| Table S5. Breakdown of bias scores for concurrent intake studies | | | | | | | | | |
| --- | --- | --- | --- | --- | --- | --- | --- | --- | --- |
| Author and Year | Self-reported energy intake (1 = Yes, 0 = No) | Eligibility criteria (1 = Missing key criteria, 0 = not missing key criteria) | Inadequate information of methodology (1 = Yes, 0 = No) | Random allocation - does the study report that random allocation has occurred? (1 = No, 0 = Yes) | Demand characteristics (1 = does not address influence of awareness, 0 = does address influence of awareness) | Small number of participants per condition (1 = Small number of participants, 0 = Sufficient number of participants) | Pre-registration (1 = Pre-registration absent, 0 = pre-registration present) | Abstaining from eating (1 = Not asked to abstain, 0 = asked to abstain) | Total Bias score |
| Bellisle and Dalix (2001) (4) | 0 | 1 | 0 | 0 | 1 | 0 | 1 | 1 | 4 |
| Bellisle, Dalix, and Slama (2004) (5) | 0 | 1 | 0 | 0 | 1 | 0 | 1 | 1 | 4 |
| Bellisle et al. (2009) (6) | 0 | 1 | 0 | 0 | 1 | 0 | 1 | 1 | 4 |
| Blass et al. (2006) (7) | 0 | 1 | 0 | 1 | 1 | 0 | 1 | 1 | 5 |
| Boon et al. (1997) (8) (Study 1) | 0 | 1 | 0 | 0 | 1 | 1 | 1 | 0 | 4 |
| Boon et al. (1997) (8) (Study 2) | 0 | 1 | 0 | 0 | 0 | 1 | 1 | 0 | 3 |
| Boon et al. (2002) (9) | 0 | 1 | 0 | 0 | 0 | 1 | 1 | 0 | 3 |
| Hetherington et al. (2006) (10) | 0 | 1 | 0 | 0 | 1 | 0 | 1 | 0 | 3 |
| Long et al. (2011) (11) | 0 | 1 | 0 | 0 | 1 | 0 | 1 | 0 | 3 |
| Martin et al. (2009) (12) | 0 | 1 | 0 | 0 | 1 | 0 | 1 | 0 | 3 |
| Kononova et al. (2018) (13) | 0 | 1 | 0 | 0 | 1 | 0 | 1 | 1 | 4 |
| Ding et al. (2019) (14) | 0 | 1 | 0 | 0 | 1 | 0 | 1 | 0 | 3 |
| Arch et al. (2016) (15) (Study 3) | 0 | 1 | 0 | 0 | 1 | 0 | 1 | 0 | 3 |
| Lyons et al. (2013) (16) | 0 | 1 | 0 | 0 | 1 | 0 | 1 | 0 | 3 |
| Stämpfli & Brunner (2016) (17) | 0 | 1 | 1 | 1 | 0 | 0 | 1 | 1 | 5 |
| Brunner (2013) (18) (Study 4) | 0 | 1 | 0 | 0 | 1 | 0 | 1 | 1 | 4 |
| da Mata Gonçalves et al. (2019) (19) | 0 | 1 | 0 | 0 | 1 | 0 | 1 | 0 | 3 |
| Ogden et al. (2013) (20) | 0 | 1 | 0 | 0 | 1 | 0 | 1 | 1 | 4 |
| van der Wal et al. (2013) (21) (Study 3) | 0 | 1 | 0 | 0 | 1 | 0 | 1 | 1 | 4 |
| Liguori et al. (2020) (22) | 0 | 1 | 0 | 0 | 1 | 0 | 1 | 0 | 3 |
| Volz et al. (2021) (23) (Study 1) | 0 | 1 | 0 | 0 | 1 | 0 | 1 | 1 | 4 |
| Volz et al. (2021) (23) (Study 2) | 0 | 1 | 0 | 0 | 1 | 0 | 1 | 1 | 4 |
| Volz et al. (2021) (23) (Study 3) | 0 | 1 | 0 | 0 | 1 | 0 | 1 | 1 | 4 |
| Volz et al. (2021) (23) (Study 4) | 0 | 1 | 0 | 0 | 1 | 0 | 1 | 1 | 4 |
| Volz et al. (2021) (23) (Study 5) | 0 | 1 | 0 | 0 | 1 | 0 | 1 | 1 | 4 |
| Volz et al. (2021) (23) (Study 6) | 0 | 1 | 0 | 0 | 1 | 0 | 1 | 1 | 4 |
| Volz et al. (2021) (23) (Study 7) | 0 | 1 | 0 | 1 | 1 | 0 | 1 | 1 | 5 |
| Mathiesen et al. (2022) (24) | 0 | 1 | 0 | 1 | 1 | 0 | 1 | 0 | 4 |
| Francis et al. (2017) (25) | 0 | 1 | 0 | 0 | 1 | 0 | 1 | 1 | 4 |
| Braude and Stevenson (2014) (26) | 0 | 1 | 0 | 0 | 1 | 0 | 1 | 0 | 3 |
| Çetin et al. (2023) (27) | 0 | 0 | 0 | 0 | 1 | 0 | 1 | 0 | 2 |
| Kaiser et al. (2016) (28) | 0 | 1 | 0 | 0 | 1 | 0 | 1 | 1 | 4 |
| Mamalaki et al. (2017) (29) | 0 | 1 | 0 | 0 | 1 | 0 | 1 | 0 | 3 |
| Rosenthal and Raynor (2017) (30) | 0 | 0 | 0 | 0 | 1 | 0 | 1 | 0 | 2 |
| Hussain et al. (2021) (31) | 0 | 1 | 0 | 0 | 1 | 0 | 1 | 1 | 4 |
| Ward and Mann (2000) (32) (Study 1) | 0 | 1 | 0 | 0 | 1 | 0 | 1 | 1 | 4 |
| Ward and Mann (2000) (32) (Study 2) | 0 | 1 | 0 | 0 | 1 | 1 | 1 | 1 | 5 |
| Lattimore and Maxwell (2004) (33) | 0 | 1 | 0 | 0 | 1 | 0 | 1 | 0 | 3 |
| Shin (2024) (34) | 0 | 1 | 0 | 0 | 1 | 0 | 1 | 1 | 4 |
| Mann and Ward (2004) (35) | 0 | 0 | 0 | 1 | 1 | 0 | 1 | 0 | 3 |

## Bias scores breakdown – later intake studies

| Table S6. Breakdown of bias scores for later intake studies | | | | | | | | | |
| --- | --- | --- | --- | --- | --- | --- | --- | --- | --- |
| Author and Year | Self-reported energy intake (1 = Yes, 0 = No) | Eligibility criteria (1 = Missing key criteria, 0 = not missing key criteria) | Inadequate information of methodology (1 = Yes, 0 = No) | Random allocation - does the study report that random allocation has occurred? (1 = No, 0 = Yes) | Demand characteristics (1 = does not address influence of awareness, 0 = does address influence of awareness) | Small number of participants per condition (1 = Small number of participants, 0 = Sufficient number of participants) | Pre-registration (1 = pre-registration absent, 0 = pre-registration present) | Abstaining from eating (1 = Not asked to abstain, 0 = asked to abstain) | Total Bias score |
| Higgs and Woodward (2009) (36) | 0 | 1 | 0 | 0 | 0 | 0 | 1 | 1 | 3 |
| Mittal et al. (2011) (37) (Study 1) | 0 | 1 | 0 | 1 | 1 | 1 | 1 | 1 | 6 |
| Mittal et al. (2011) (37) (Study 2) | 0 | 1 | 0 | 0 | 1 | 0 | 1 | 0 | 3 |
| Oldham-Cooper et al. (2010) (38) | 0 | 1 | 0 | 0 | 1 | 0 | 1 | 0 | 3 |
| Higgs et al. (2015) (39) (Study 1) | 0 | 1 | 0 | 0 | 0 | 1 | 1 | 1 | 4 |
| Higgs et al. (2015) (39) (Study 2) | 0 | 1 | 0 | 0 | 0 | 0 | 1 | 0 | 2 |
| Morris et al. (2020) (40) | 0 | 1 | 0 | 1 | 1 | 0 | 1 | 0 | 4 |
| Whitelock et al. (2018) (41)(Study 1) | 0 | 1 | 0 | 0 | 0 | 0 | 1 | 0 | 2 |
| van Meer et al. (2023) (42) | 0 | 1 | 0 | 0 | 1 | 0 | 1 | 0 | 3 |
| Duif et al. (2020) (43) | 0 | 1 | 0 | 0 | 1 | 0 | 0 | 0 | 2 |

# List of excluded studies with reasons for exclusions

Table S7. List of studies which were excluded after full-text screening, with reasons for exclusion stated.

| **Study** | **Reason(s) for exclusion** |
| --- | --- |
| Alblas et al. (2023)(44) | Does not use an experimental design. |
| Bravo-Moncayo et al. (2020)(45) | Does not have an appropriate control condition. |
| Chapman et al. (2014) (46) | The study does not have a clear control condition – all conditions involve reading or watching television. |
| Dieze et al. (2017)(47) | Not a valid distractor. The study looks at TV viewing through speakers or headphones – the manipulation relates to how immersive the stimuli is. |
| Duif et al. (2020) (48) | Study does not measure energy intake. |
| Fisher et al. (2016)(49) | Distraction does not occur during consumption of energy. |
| Hock and Bagchi (2018)(50) | Study does not use a valid distractor – eating in under distraction of a crowded environment may be confounded by social factors. |
| Hoffmann-Hensel et al. (2017)(51) | Study does not measure food intake. |
| Hussain (2021)(31) | Does not manipulate level of distraction. |
| Kappattanavar et al. (2023)(52) | Distraction does not occur during consumption of energy. |
| Maruya et al. (2019)(53) | The study uses an educational dietary song as background music – this may confound energy intake. |
| Mason et al. (2016) (54) | The study does not experimentally manipulate levels of distraction. |
| Mathiesen et al. (2020)(55) | The study does not measure energy intake. |
| Mathur and Stevenson (2015)(56) | All conditions are exposed to television (there is no appropriate control condition). |
| Michels and Hamers (2023) (57) | No appropriate control condition and distraction does not occur during energy intake. |
| Migliavada et al. (2024) (58) | Does not include an appropriate control condition – only music tempo is manipulated across conditions. |
| Morris et al. (2020) (59) | Does not measure energy intake. |
| Murphy et al. (2024) (60) | Energy intake is measured using a self-report Likert scale. |
| Murphy et al. (2022) (61) | The study does not use an experimental design. |
| Norberg et al. (2023) (62) | The study does not use a valid distractor (untidy room). |
| Ogden et al. (2018) (63) | The study does not experimentally manipulate levels of distraction. |
| Periman (2015) (64) | All participants were exposed to television – there was no appropriate control condition. |
| Raynor et al. (2013) (65) | The study does not explicitly require participants to reduce television watching whilst consuming energy. |
| Robinson et al. (2014) (66) | The study does not include a distraction condition. |
| Ruda et al. (2024) (67) | The study does not measure energy intake. |
| Ruda et al. (2024) (68) | The study does not measure energy intake. |
| Sato et al. (2023) (69) | The study does not include an appropriate control condition. |
| Seguias and Tapper (2022) (70) | The study does not manipulate the level of distraction. |
| Seguias et al. (2025) (71) | The study does not manipulate the level of distraction. |
| Seguias (2019) (72) | The studies in this thesis do not manipulate the level of distraction. |
| Siangphloen et al. (2024) (73) | The study does not measure energy intake. |
| Siervo et al. (2018) (74) | The distraction phase does not occur during energy intake. |
| Tsai et al. (2017) (75) | The distraction phase occurs after energy intake. |
| van Meer et al. (2023) (76) | The study does not measure energy intake. |
| Whitelock et al. (2019) (77) | The study does not manipulate the level of distraction. |
| Whitelock and Robinson (2018) (78) | The study does not manipulate the level of distraction. |
| Whitelock et al. (2019) (79) | The study does not manipulate the level of distraction. |
| Zimmerman and Shimoga (2014) (80) | The distraction phase does not occur during energy intake. |
| Albajri (2020) (81) | The study does not manipulate the level of distraction. |
| Bourn et al. (2015) (82) | The study does not manipulate the level of distraction. |
| Hurst (2023) (83) | Compares intake when eating with someone else vs eating alone (not a valid distractor) |
| Kaiwa et al. (2023) (84) | The study does not use a valid distractor. |
| Korsgaard (2019) (85) | The study does not manipulate the level of distraction. |
| Lemke and Schifferstein (2021) (86) | Uses qualitative methodology. |
| Lock et al. (2016) (87) | The study does not use an experimental design. |
| Mantzios et al. (2020) (88) | The study does not manipulate the level of distraction. |
| Ogden et al. (2021) (89) | The distraction phase does not occur during energy intake. |
| Privitera et al. (2014) (90) | The study does not include a control condition. |
| Putri et al. (2024) (91) | The study does not use an experimental design. |
| Seguias and Tapper (2018) (92) | The study does not manipulate the level of distraction. |
| van Nee et al. (2016) (93) | The study does not manipulate the level of distraction. |
| Veldhuizen (2017) (94) | The article reports a summary of a different empirical study already reviewed. |
| Wischmann (2020) (95) | The study does not use an experimental design. |
| Bolhuis (2012) (96) | The study compares intake between a distraction condition and a focussed attention condition – there is no appropriate control condition. |
| Dibay Moghadam (2017) (97) | The study does not manipulate the level of distraction. |
| Rogers et al. (2021) (98) | The study instructed participants to consume all of the food given to them (not ad libitum intake). |
| Van Dillen and Andrade (2016) (99) | The study does not measure energy intake. |
| McAlister and Kononova (2022) (100) | The study uses the same sample as a study already included in this review. |
| Hunter et al. (2019) (101) | The study does not measure energy intake. |
| Moynihan et al. (2015) (102) | The study does not manipulate the level of distraction. |
| Hulbert-Williams et al. (2019) (103) | The study does not use a valid distractor. |
| Van den Tol et al. (2022) (104) | The distraction phase does not occur during energy intake. |
| La Marra et al. (2020) (105) | This is a review article. |
| Eschenbeck et al. (2016) (106) | The study does not measure energy intake. |
| Trooper (2016) (107) | The distraction phase does not occur during energy intake. |
| Ogden et al. (2017) (108) | The distraction phase does not occur during energy intake. |
| Argo and White (Study 5) (2012) (109) | Unable to acquire energy intake data. |
| Volz et al. (Study 8) (2021) (23) | Unable to acquire energy intake data. |
| Van der Wal (Study 4) (2013) (21) | The study does not measure energy intake. |

# References

1. Alblas MC, Mollen S, Wennekers AM, Fransen ML, van den Putte B. Consuming media, consuming food: investigating concurrent TV viewing and eating using a 7-d time use diary survey. Public health nutrition. 2023;26(4):748-57.

2. Bravo-Moncayo L, Reinoso-Carvalho F, Velasco C. The effects of noise control in coffee tasting experiences. Food Quality and Preference. 2020;86:104020.

3. Chapman CD, Nilsson VC, Thune HÅ, Cedernaes J, Le Grevès M, Hogenkamp PS, et al. Watching TV and food intake: the role of content. PLoS One. 2014;9(7):e100602.

4. Dieze A, Stephan T, Hilzendegen C, Stroebele-Benschop N. The impact of viewing a video with and without head phones on snack intake: A pilot study. Plos one. 2017;12(12):e0188457.

5. Duif I, Wegman J, de Graaf K, Smeets PA, Aarts E. Distraction decreases rIFG-putamen connectivity during goal-directed effort for food rewards. Scientific reports. 2020;10(1):19072.

6. Fisher N, Lattimore P, Malinowski P. Attention with a mindful attitude attenuates subjective appetitive reactions and food intake following food-cue exposure. Appetite. 2016;99:10-6.

7. Hock SJ, Bagchi R. The impact of crowding on calorie consumption. Journal of Consumer Research. 2018;44(5):1123-40.

8. Hoffmann-Hensel SM, Sijben R, Rodriguez-Raecke R, Freiherr J. Cognitive load alters neuronal processing of food odors. Chemical senses. 2017;42(9):723-36.

9. Hussain M. Exploring the effects of mindful eating, self-kindness and self-distancing on promoting healthier eating behaviours: Birmingham City University; 2021.

10. Kappattanavar AM, Hecker P, Moontaha S, Steckhan N, Arnrich B. Food choices after cognitive load: an affective computing approach. Sensors. 2023;23(14):6597.

11. Maruya S, Sato Y, Nakai H, Takachi R. The effect of educational background music on reducing salt intake at a university canteen. Open Access J Public Health. 2019;2:028.

12. Mason AE, Epel ES, Kristeller J, Moran PJ, Dallman M, Lustig RH, et al. Effects of a mindfulness-based intervention on mindful eating, sweets consumption, and fasting glucose levels in obese adults: data from the SHINE randomized controlled trial. Journal of behavioral medicine. 2016;39:201-13.

13. Mathiesen SL, Mielby LA, Byrne DV, Wang QJ. Music to eat by: A systematic investigation of the relative importance of tempo and articulation on eating time. Appetite. 2020;155:104801.

14. Mathur U, Stevenson RJ. Television and eating: repetition enhances food intake. Frontiers in psychology. 2015;6:1657.

15. Michels N, Hamers P. Nature sounds for stress recovery and healthy eating: a lab experiment differentiating water and bird sound. Environment and Behavior. 2023;55(3):175-205.

16. Migliavada R, Luceri F, Torri L. Chew that beat! How music tempo influences eating behaviors and emotions. Food Quality and Preference. 2024;118:105195.

17. Morris J, Yeomans MR, Forster S. Testing a load theory framework for food-related cognition. Journal of Experimental Psychology: General. 2020;149(12):2406.

18. Murphy SL, van Meer F, van Dillen L, van Steenbergen H, Hofmann W. Underwhelming pleasures: Toward a self-regulatory account of hedonic compensation and overconsumption. Journal of Personality and Social Psychology. 2024.

19. Murphy SL, van Meer F, van Steenbergen H, Hofmann W. Hedonic Compensation and Overconsumption: An Experience Sampling Investigation. 2022.

20. Norberg MM, Stevenson RJ, Wong G, Tame J, Aldrich P, Meares S, et al. Situational, emotional, and individual dispositions to weight gain in people with hoarding problems. British Journal of Clinical Psychology. 2023;62(2):501-17.

21. Ogden J, Wood C, Payne E, Fouracre H, Lammyman F. ‘Snack’versus ‘meal’: The impact of label and place on food intake. Appetite. 2018;120:666-72.

22. Periman SA. The Effect of Perceived Healthy and Unhealthy Commercials on Intake of Perceived Healthy and Unhealthy Snack foods in Normal Weight, College-Aged, Dietary Restrained Women. 2015.

23. Raynor HA, Steeves EA, Bassett Jr DR, Thompson DL, Gorin AA, Bond DS. Reducing TV watching during adult obesity treatment: two pilot randomized controlled trials. Behavior therapy. 2013;44(4):674-85.

24. Robinson E, Kersbergen I, Higgs S. Eating ‘attentively’reduces later energy consumption in overweight and obese females. British Journal of Nutrition. 2014;112(4):657-61.

25. Ruda I, Chellapandian DC, Freiherr J. The impact of cognitive distraction on gustatory perception in volunteers with obesity. Scientific Reports. 2024;14(1):14268.

26. Ruda I, Chellapandian DC, Rott M, Scheid S, Freiherr J. Beyond Distracted Eating: Cognitive Distraction Downregulates Odor Pleasantness and Interacts with Weight Status. Nutrients. 2024;16(17):2871.

27. Sato N, Miyamoto M, Santa R, Homma C, Shibuya K. The effect of background music tempo on eating speed and food intake volume within in healthy women. Nutrition and Health. 2023:02601060231158234.

28. Seguias L, Tapper K. A randomized controlled trial examining the effects of mindful eating and eating without distractions on food intake over a three-day period. Nutrients. 2022;14(5):1043.

29. Seguias L, Ferriday D, Hinton EC, McCaw T, Tapper K. Mindful eating and food intake: Effects and mechanisms of action. Journal of Experimental Psychology: Applied. 2025.

30. Seguias L. The effects of mindful eating on food intake and diet: City, University of London; 2019.

31. Siangphloen P, Shepherd D, Kantono K, Hamid N. Lunch melodies: Investigating the impact of music on emotions, hunger, liking, and psychophysiology while viewing a lunch meal. Food Research International. 2024;192:114825.

32. Siervo M, Gan J, Fewtrell MS, Cortina-Borja M, Wells JC. Acute effects of video-game playing versus television viewing on stress markers and food intake in overweight and obese young men: A randomised controlled trial. Appetite. 2018;120:100-8.

33. Tsai A, Hughes EK, Fuller-Tyszkiewicz M, Buck K, Krug I. The differential effects of mindfulness and distraction on affect and body satisfaction following food consumption. Frontiers in psychology. 2017;8:1696.

34. van Meer F, van Steenbergen H, van Dillen LF. The effect of cognitive load on preference and intensity processing of sweet taste in the brain. Appetite. 2023;188:106630.

35. Whitelock V, Gaglione A, Davies-Owen J, Robinson E. Focused attention during eating enhanced memory for meal satiety but did not reduce later snack intake in men: A randomised within-subjects laboratory experiment. Appetite. 2019;136:124-9.

36. Whitelock V, Robinson E. Remembered meal satisfaction, satiety, and later snack food intake: a laboratory study. Nutrients. 2018;10(12):1883.

37. Whitelock V, Kersbergen I, Higgs S, Aveyard P, Halford JC, Robinson E. A smartphone based attentive eating intervention for energy intake and weight loss: results from a randomised controlled trial. BMC Public Health. 2019;19:1-11.

38. Zimmerman FJ, Shimoga SV. The effects of food advertising and cognitive load on food choices. BMC public health. 2014;14:1-10.

39. Albajri E. Modulation of Cognitive Restraint Mediated Effects on the Prefrontal Cortex Response During Eating of Preferred High Fat/High Sugar Foods in Women as Measured by fNir: Drexel University; 2020.

40. Bourn R, Prichard I, Hutchinson AD, Wilson C. Watching reality weight loss TV. The effects on body satisfaction, mood, and snack food consumption. Appetite. 2015;91:351-6.

41. Hurst K. Social Influences on Eating: Analyzed by the Mandometer and Questionnaire 2023.

42. Kaiwa M, Kinoshita N, Inaba H. Is Food More Delicious When Eaten Alone or When via the Internet? Journal of Japanese Society of Shokuiku. 2023;17(2):81-9.

43. Korsgaard DM. Immersive Eating: The virtually enhanced solitary meal context as a strategy to promote positive meal experiences and sufficient energy intake for future generations of older adults. 2019.

44. Lemke M, Schifferstein HN. The use of ICT devices as part of the solo eating experience. Appetite. 2021;165:105297.

45. Lock C, Brindal E, Hendrie GA, Cox DN. Contextual and environmental influences on reported dietary energy intake at evening eating occasions. Eating behaviors. 2016;21:155-60.

46. Mantzios M, Egan H, Asif T. A randomised experiment evaluating the mindful raisin practice as a method of reducing chocolate consumption during and after a mindless activity. Journal of Cognitive Enhancement. 2020;4(3):250-7.

47. Ogden J, Biliraki C, Ellis A, Lammyman F, May E. The impact of active or passive food preparation versus distraction on eating behaviour: An experimental study. Appetite. 2021;160:105072.

48. Privitera GJ, Diaz M, Haas MC. Enhanced auditory arousal increases intake of less palatable and healthier foods. Global Journal of Health Science. 2014;6(3):1.

49. Putri WA, Widodo A, Solihat R. Is there any Difference between Males and Females in Mindful Eating? Prisma Sains: Jurnal Pengkajian Ilmu dan Pembelajaran Matematika dan IPA IKIP Mataram. 2024;12(1):49-60.

50. Seguias L, Tapper K. The effect of mindful eating on subsequent intake of a high calorie snack. Appetite. 2018;121:93-100.

51. van Nee RL, Larsen JK, Fisher JO. Direct effects of food cues seen during TV viewing on energy intake in young women. Appetite. 2016;101:80-5.

52. Veldhuizen MG. Distracted sniffing of food odors leads to diminished behavioral and neural responses. Chemical Senses. 2017;42(9):719-22.

53. Wischmann M. An experience sampling study on binge watching and its relation to healthy and unhealthy snacking: University of Twente; 2020.

54. Bolhuis DP. The role of oral exposure to taste on meal termination: Wageningen University and Research; 2012.

55. Dibay Moghadam S. Stress, Obesogenic Behaviors, Measures of Obesity Risk Among Hispanic and Non-Hispanic White Women 2017.

56. Rogers PJ, Drumgoole FD, Quinlan E, Thompson Y. An analysis of sensory-specific satiation: Food liking, food wanting, and the effects of distraction. Learning and Motivation. 2021;73:101688.

57. van Dillen LF, Andrade J. Derailing the streetcar named desire. Cognitive distractions reduce individual differences in cravings and unhealthy snacking in response to palatable food. Appetite. 2016;96:102-10.

58. McAlister AR, Kononova A. Consumption of fruits, vegetables, and nuts can be increased when multitasking with screen devices. Health Communication. 2022;37(2):141-51.

59. Hunter J, Hollands G, Pilling M, Marteau T. Impact of proximity of healthier versus less healthy foods on intake: A lab-based experiment. Appetite. 2019;133:147-55.

60. Moynihan AB, Tilburg WAv, Igou ER, Wisman A, Donnelly AE, Mulcaire JB. Eaten up by boredom: consuming food to escape awareness of the bored self. Frontiers in psychology. 2015;6:369.

61. Hulbert-Williams L, Hulbert-Williams NJ, Nicholls W, Williamson S, Poonia J, Hochard KD. Ultra-brief non-expert-delivered defusion and acceptance exercises for food cravings: A partial replication study. Journal of health psychology. 2019;24(12):1698-709.

62. van den Tol AJ, Coulthard H, Lang V, Wallis DJ. Are music listening strategies associated with reduced food consumption following negative mood inductions; a series of three exploratory experimental studies. Appetite. 2022;172:105947.

63. La Marra M, Caviglia G, Perrella R. Using smartphones when eating increases caloric intake in young people: an overview of the literature. Frontiers in Psychology. 2020;11:587886.

64. Eschenbeck H, Heim-Dreger U, Steinhilber A, Kohlmann C-W. Self-regulation of healthy nutrition: automatic and controlled processes. BMC psychology. 2016;4:1-8.

65. Tropper A. Effects of Self-Regulation and cognitive load on accessibility of temptation thoughts in restrained eaters: Hofstra University; 2016.

66. Ogden J, Oikonomou E, Alemany G. Distraction, restrained eating and disinhibition: an experimental study of food intake and the impact of ‘eating on the go’. Journal of health psychology. 2017;22(1):39-50.

67. Argo JJ, White K. When do consumers eat more? The role of appearance self-esteem and food packaging cues. Journal of Marketing. 2012;76(2):67-80.

68. Volz S, Ward A, Mann T. Eating up cognitive resources: Does attentional consumption lead to food consumption? Appetite. 2021;162:105165.

69. van der Wal RC, van Dillen LF. Leaving a flat taste in your mouth: task load reduces taste perception. Psychological science. 2013;24(7):1277-84.

1. Robinson E, Aveyard P, Daley A, Jolly K, Lewis A, Lycett D, et al. Eating attentively: a systematic review and meta-analysis of the effect of food intake memory and awareness on eating. The American journal of clinical nutrition. 2013;97(4):728-42.

2. Simonsohn U, Nelson LD, Simmons JP. P-curve: a key to the file-drawer. Journal of experimental psychology: General. 2014;143(2):534.

3. Morey RD, Davis-Stober CP. On the poor statistical properties of the P-curve meta-analytic procedure. Journal of the American Statistical Association. 2025(just-accepted):1-19.

4. Bellisle F, Dalix A-M. Cognitive restraint can be offset by distraction, leading to increased meal intake in women. The American journal of clinical nutrition. 2001;74(2):197-200.

5. Bellisle F, Dalix AM, Slama G. Non food-related environmental stimuli induce increased meal intake in healthy women: comparison of television viewing versus listening to a recorded story in laboratory settings. Appetite. 2004;43(2):175-80.

6. Bellisle F, Dalix A-M, Airinei G, Hercberg S, Péneau S. Influence of dietary restraint and environmental factors on meal size in normal-weight women. A laboratory study. Appetite. 2009;53(3):309-13.

7. Blass EM, Anderson DR, Kirkorian HL, Pempek TA, Price I, Koleini MF. On the road to obesity: Television viewing increases intake of high-density foods. Physiology & behavior. 2006;88(4-5):597-604.

8. Boon B, Stroebe W, Schut H, Jansen A. Does cognitive distraction lead to overeating in restrained eaters? Behavioural and Cognitive Psychotherapy. 1997;25(4):319-27.

9. Boon B, Stroebe W, Schut H, IJntema R. Ironic processes in the eating behaviour of restrained eaters. British journal of health psychology. 2002;7(1):1-10.

10. Hetherington MM, Anderson AS, Norton GN, Newson L. Situational effects on meal intake: A comparison of eating alone and eating with others. Physiology & behavior. 2006;88(4-5):498-505.

11. Long S, Meyer C, Leung N, Wallis DJ. Effects of distraction and focused attention on actual and perceived food intake in females with non-clinical eating psychopathology. Appetite. 2011;56(2):350-6.

12. Martin CK, Coulon SM, Markward N, Greenway FL, Anton SD. Association between energy intake and viewing television, distractibility, and memory for advertisements. The American journal of clinical nutrition. 2009;89(1):37-44.

13. Kononova A, McAlister A, Oh HJ. Screen overload: Pleasant multitasking with screen devices leads to the choice of healthful over less healthful snacks when compared with unpleasant multitasking. Computers in human behavior. 2018;80:1-11.

14. Ding F, Hamid N, Shepherd D, Kantono K. How is satiety affected when consuming food while working on a computer? Nutrients. 2019;11(7):1545.

15. Arch JJ, Brown KW, Goodman RJ, Della Porta MD, Kiken LG, Tillman S. Enjoying food without caloric cost: The impact of brief mindfulness on laboratory eating outcomes. Behaviour research and therapy. 2016;79:23-34.

16. Lyons EJ, Tate DF, Ward DS, Wang X. Energy intake and expenditure during sedentary screen time and motion-controlled video gaming. The American journal of clinical nutrition. 2012;96(2):234-9.

17. Stämpfli AE, Brunner TA. The art of dieting: Exposure to thin sculptures effortlessly reduces the intake of unhealthy food in motivated eaters. Food Quality and Preference. 2016;50:88-93.

18. Brunner TA. It takes some effort. How minimal physical effort reduces consumption volume. Appetite. 2013;71:89-94.

19. da Mata Gonçalves RF, de Almeida Barreto D, Monteiro PI, Zangeronimo MG, Castelo PM, van der Bilt A, et al. Smartphone use while eating increases caloric ingestion. Physiology & behavior. 2019;204:93-9.

20. Ogden J, Coop N, Cousins C, Crump R, Field L, Hughes S, et al. Distraction, the desire to eat and food intake. Towards an expanded model of mindless eating. Appetite. 2013;62:119-26.

21. van der Wal RC, van Dillen LF. Leaving a flat taste in your mouth: task load reduces taste perception. Psychological science. 2013;24(7):1277-84.

22. Liguori CA, Nikolaus CJ, Nickols-Richardson SM. Cognitive distraction at mealtime decreases amount consumed in healthy young adults: A randomized crossover exploratory study. The Journal of nutrition. 2020;150(5):1324-9.

23. Volz S, Ward A, Mann T. Eating up cognitive resources: Does attentional consumption lead to food consumption? Appetite. 2021;162:105165.

24. Mathiesen SL, Hopia A, Ojansivu P, Byrne DV, Wang QJ. The sound of silence: Presence and absence of sound affects meal duration and hedonic eating experience. Appetite. 2022;174:106011.

25. Francis HM, Stevenson RJ, Oaten MJ, Mahmut MK, Yeomans MR. The immediate and delayed effects of TV: impacts of gender and processed-food intake history. Frontiers in psychology. 2017;8:1616.

26. Braude L, Stevenson RJ. Watching television while eating increases energy intake. Examining the mechanisms in female participants. Appetite. 2014;76:9-16.

27. Çetin C, Ayaz A, Samur G, Akyol A. Music genre and volume do not modulate energy intake, short-term satiety, and mood states: A randomized controlled trial. Psychology of Music. 2023;51(4):1101-18.

28. Kaiser D, Silberberger S, Hilzendegen C, Stroebele-Benschop N. The influence of music type and transmission mode on food intake and meal duration: An experimental study. Psychology of Music. 2016;44(6):1419-30.

29. Mamalaki E, Zachari K, Karfopoulou E, Zervas E, Yannakoulia M. Presence of music while eating: Effects on energy intake, eating rate and appetite sensations. Physiology & Behavior. 2017;168:31-3.

30. Rosenthal R, Raynor H. The effect of television watching and portion size on intake during a meal. Appetite. 2017;117:191-6.

31. Hussain M. Exploring the effects of mindful eating, self-kindness and self-distancing on promoting healthier eating behaviours: Birmingham City University; 2021.

32. Ward A, Mann T. Don't mind if I do: disinhibited eating under cognitive load. Journal of personality and social psychology. 2000;78(4):753.

33. Lattimore P, Maxwell L. Cognitive load, stress, and disinhibited eating. Eating Behaviors. 2004;5(4):315-24.

34. Shin S. Effects of distractions such as audio, audiovisual, and hand-use on food intake and satiety ratings. Journal of Nutrition and Health. 2024;57(3):275-81.

35. Mann T, Ward A. To eat or not to eat: implications of the attentional myopia model for restrained eaters. Journal of Abnormal Psychology. 2004;113(1):90.

36. Higgs S, Woodward M. Television watching during lunch increases afternoon snack intake of young women. Appetite. 2009;52(1):39-43.

37. Mittal D, Stevenson RJ, Oaten MJ, Miller LA. Snacking while watching TV impairs food recall and promotes food intake on a later TV free test meal. Applied Cognitive Psychology. 2011;25(6):871-7.

38. Oldham-Cooper RE, Hardman CA, Nicoll CE, Rogers PJ, Brunstrom JM. Playing a computer game during lunch affects fullness, memory for lunch, and later snack intake. The American journal of clinical nutrition. 2011;93(2):308-13.

39. Higgs S. Manipulations of attention during eating and their effects on later snack intake. Appetite. 2015;92:287-94.

40. Morris J, Vi CT, Obrist M, Forster S, Yeomans MR. Ingested but not perceived: response to satiety cues disrupted by perceptual load. Appetite. 2020;155:104813.

41. Whitelock V, Higgs S, Brunstrom JM, Halford JC, Robinson E. No effect of focused attention whilst eating on later snack food intake: Two laboratory experiments. Appetite. 2018;128:188-96.

42. van Meer F, Murphy SL, Hofmann W, Van Steenbergen H, Van Dillen LF. Driven to snack: Simulated driving increases subsequent consumption. Journal of Trial & Error. 2023;3(1):57-71.

43. Duif I, Wegman J, Mars MM, De Graaf C, Smeets PA, Aarts E. Effects of distraction on taste-related neural processing: a cross-sectional fMRI study. The American journal of clinical nutrition. 2020;111(5):950-61.

44. Alblas MC, Mollen S, Wennekers AM, Fransen ML, van den Putte B. Consuming media, consuming food: investigating concurrent TV viewing and eating using a 7-d time use diary survey. Public health nutrition. 2023;26(4):748-57.

45. Bravo-Moncayo L, Reinoso-Carvalho F, Velasco C. The effects of noise control in coffee tasting experiences. Food Quality and Preference. 2020;86:104020.

46. Chapman CD, Nilsson VC, Thune HÅ, Cedernaes J, Le Grevès M, Hogenkamp PS, et al. Watching TV and food intake: the role of content. PLoS One. 2014;9(7):e100602.

47. Dieze A, Stephan T, Hilzendegen C, Stroebele-Benschop N. The impact of viewing a video with and without head phones on snack intake: A pilot study. Plos one. 2017;12(12):e0188457.

48. Duif I, Wegman J, de Graaf K, Smeets PA, Aarts E. Distraction decreases rIFG-putamen connectivity during goal-directed effort for food rewards. Scientific reports. 2020;10(1):19072.

49. Fisher N, Lattimore P, Malinowski P. Attention with a mindful attitude attenuates subjective appetitive reactions and food intake following food-cue exposure. Appetite. 2016;99:10-6.

50. Hock SJ, Bagchi R. The impact of crowding on calorie consumption. Journal of Consumer Research. 2018;44(5):1123-40.

51. Hoffmann-Hensel SM, Sijben R, Rodriguez-Raecke R, Freiherr J. Cognitive load alters neuronal processing of food odors. Chemical senses. 2017;42(9):723-36.

52. Kappattanavar AM, Hecker P, Moontaha S, Steckhan N, Arnrich B. Food choices after cognitive load: an affective computing approach. Sensors. 2023;23(14):6597.

53. Maruya S, Sato Y, Nakai H, Takachi R. The effect of educational background music on reducing salt intake at a university canteen. Open Access J Public Health. 2019;2:028.

54. Mason AE, Epel ES, Kristeller J, Moran PJ, Dallman M, Lustig RH, et al. Effects of a mindfulness-based intervention on mindful eating, sweets consumption, and fasting glucose levels in obese adults: data from the SHINE randomized controlled trial. Journal of behavioral medicine. 2016;39:201-13.

55. Mathiesen SL, Mielby LA, Byrne DV, Wang QJ. Music to eat by: A systematic investigation of the relative importance of tempo and articulation on eating time. Appetite. 2020;155:104801.

56. Mathur U, Stevenson RJ. Television and eating: repetition enhances food intake. Frontiers in psychology. 2015;6:1657.

57. Michels N, Hamers P. Nature sounds for stress recovery and healthy eating: a lab experiment differentiating water and bird sound. Environment and Behavior. 2023;55(3):175-205.

58. Migliavada R, Luceri F, Torri L. Chew that beat! How music tempo influences eating behaviors and emotions. Food Quality and Preference. 2024;118:105195.

59. Morris J, Yeomans MR, Forster S. Testing a load theory framework for food-related cognition. Journal of Experimental Psychology: General. 2020;149(12):2406.

60. Murphy SL, van Meer F, van Dillen L, van Steenbergen H, Hofmann W. Underwhelming pleasures: Toward a self-regulatory account of hedonic compensation and overconsumption. Journal of Personality and Social Psychology. 2024.

61. Murphy SL, van Meer F, van Steenbergen H, Hofmann W. Hedonic Compensation and Overconsumption: An Experience Sampling Investigation. 2022.

62. Norberg MM, Stevenson RJ, Wong G, Tame J, Aldrich P, Meares S, et al. Situational, emotional, and individual dispositions to weight gain in people with hoarding problems. British Journal of Clinical Psychology. 2023;62(2):501-17.

63. Ogden J, Wood C, Payne E, Fouracre H, Lammyman F. ‘Snack’versus ‘meal’: The impact of label and place on food intake. Appetite. 2018;120:666-72.

64. Periman SA. The Effect of Perceived Healthy and Unhealthy Commercials on Intake of Perceived Healthy and Unhealthy Snack foods in Normal Weight, College-Aged, Dietary Restrained Women. 2015.

65. Raynor HA, Steeves EA, Bassett Jr DR, Thompson DL, Gorin AA, Bond DS. Reducing TV watching during adult obesity treatment: two pilot randomized controlled trials. Behavior therapy. 2013;44(4):674-85.

66. Robinson E, Kersbergen I, Higgs S. Eating ‘attentively’reduces later energy consumption in overweight and obese females. British Journal of Nutrition. 2014;112(4):657-61.

67. Ruda I, Chellapandian DC, Freiherr J. The impact of cognitive distraction on gustatory perception in volunteers with obesity. Scientific Reports. 2024;14(1):14268.

68. Ruda I, Chellapandian DC, Rott M, Scheid S, Freiherr J. Beyond Distracted Eating: Cognitive Distraction Downregulates Odor Pleasantness and Interacts with Weight Status. Nutrients. 2024;16(17):2871.

69. Sato N, Miyamoto M, Santa R, Homma C, Shibuya K. The effect of background music tempo on eating speed and food intake volume within in healthy women. Nutrition and Health. 2023:02601060231158234.

70. Seguias L, Tapper K. A randomized controlled trial examining the effects of mindful eating and eating without distractions on food intake over a three-day period. Nutrients. 2022;14(5):1043.

71. Seguias L, Ferriday D, Hinton EC, McCaw T, Tapper K. Mindful eating and food intake: Effects and mechanisms of action. Journal of Experimental Psychology: Applied. 2025.

72. Seguias L. The effects of mindful eating on food intake and diet: City, University of London; 2019.

73. Siangphloen P, Shepherd D, Kantono K, Hamid N. Lunch melodies: Investigating the impact of music on emotions, hunger, liking, and psychophysiology while viewing a lunch meal. Food Research International. 2024;192:114825.

74. Siervo M, Gan J, Fewtrell MS, Cortina-Borja M, Wells JC. Acute effects of video-game playing versus television viewing on stress markers and food intake in overweight and obese young men: A randomised controlled trial. Appetite. 2018;120:100-8.

75. Tsai A, Hughes EK, Fuller-Tyszkiewicz M, Buck K, Krug I. The differential effects of mindfulness and distraction on affect and body satisfaction following food consumption. Frontiers in psychology. 2017;8:1696.

76. van Meer F, van Steenbergen H, van Dillen LF. The effect of cognitive load on preference and intensity processing of sweet taste in the brain. Appetite. 2023;188:106630.

77. Whitelock V, Gaglione A, Davies-Owen J, Robinson E. Focused attention during eating enhanced memory for meal satiety but did not reduce later snack intake in men: A randomised within-subjects laboratory experiment. Appetite. 2019;136:124-9.

78. Whitelock V, Robinson E. Remembered meal satisfaction, satiety, and later snack food intake: a laboratory study. Nutrients. 2018;10(12):1883.

79. Whitelock V, Kersbergen I, Higgs S, Aveyard P, Halford JC, Robinson E. A smartphone based attentive eating intervention for energy intake and weight loss: results from a randomised controlled trial. BMC Public Health. 2019;19:1-11.

80. Zimmerman FJ, Shimoga SV. The effects of food advertising and cognitive load on food choices. BMC public health. 2014;14:1-10.

81. Albajri E. Modulation of Cognitive Restraint Mediated Effects on the Prefrontal Cortex Response During Eating of Preferred High Fat/High Sugar Foods in Women as Measured by fNir: Drexel University; 2020.

82. Bourn R, Prichard I, Hutchinson AD, Wilson C. Watching reality weight loss TV. The effects on body satisfaction, mood, and snack food consumption. Appetite. 2015;91:351-6.

83. Hurst K. Social Influences on Eating: Analyzed by the Mandometer and Questionnaire 2023.

84. Kaiwa M, Kinoshita N, Inaba H. Is Food More Delicious When Eaten Alone or When via the Internet? Journal of Japanese Society of Shokuiku. 2023;17(2):81-9.

85. Korsgaard DM. Immersive Eating: The virtually enhanced solitary meal context as a strategy to promote positive meal experiences and sufficient energy intake for future generations of older adults. 2019.

86. Lemke M, Schifferstein HN. The use of ICT devices as part of the solo eating experience. Appetite. 2021;165:105297.

87. Lock C, Brindal E, Hendrie GA, Cox DN. Contextual and environmental influences on reported dietary energy intake at evening eating occasions. Eating behaviors. 2016;21:155-60.

88. Mantzios M, Egan H, Asif T. A randomised experiment evaluating the mindful raisin practice as a method of reducing chocolate consumption during and after a mindless activity. Journal of Cognitive Enhancement. 2020;4(3):250-7.

89. Ogden J, Biliraki C, Ellis A, Lammyman F, May E. The impact of active or passive food preparation versus distraction on eating behaviour: An experimental study. Appetite. 2021;160:105072.

90. Privitera GJ, Diaz M, Haas MC. Enhanced auditory arousal increases intake of less palatable and healthier foods. Global Journal of Health Science. 2014;6(3):1.

91. Putri WA, Widodo A, Solihat R. Is there any Difference between Males and Females in Mindful Eating? Prisma Sains: Jurnal Pengkajian Ilmu dan Pembelajaran Matematika dan IPA IKIP Mataram. 2024;12(1):49-60.

92. Seguias L, Tapper K. The effect of mindful eating on subsequent intake of a high calorie snack. Appetite. 2018;121:93-100.

93. van Nee RL, Larsen JK, Fisher JO. Direct effects of food cues seen during TV viewing on energy intake in young women. Appetite. 2016;101:80-5.

94. Veldhuizen MG. Distracted sniffing of food odors leads to diminished behavioral and neural responses. Chemical Senses. 2017;42(9):719-22.

95. Wischmann M. An experience sampling study on binge watching and its relation to healthy and unhealthy snacking: University of Twente; 2020.

96. Bolhuis DP. The role of oral exposure to taste on meal termination: Wageningen University and Research; 2012.

97. Dibay Moghadam S. Stress, Obesogenic Behaviors, Measures of Obesity Risk Among Hispanic and Non-Hispanic White Women 2017.

98. Rogers PJ, Drumgoole FD, Quinlan E, Thompson Y. An analysis of sensory-specific satiation: Food liking, food wanting, and the effects of distraction. Learning and Motivation. 2021;73:101688.

99. van Dillen LF, Andrade J. Derailing the streetcar named desire. Cognitive distractions reduce individual differences in cravings and unhealthy snacking in response to palatable food. Appetite. 2016;96:102-10.

100. McAlister AR, Kononova A. Consumption of fruits, vegetables, and nuts can be increased when multitasking with screen devices. Health Communication. 2022;37(2):141-51.

101. Hunter J, Hollands G, Pilling M, Marteau T. Impact of proximity of healthier versus less healthy foods on intake: A lab-based experiment. Appetite. 2019;133:147-55.

102. Moynihan AB, Tilburg WAv, Igou ER, Wisman A, Donnelly AE, Mulcaire JB. Eaten up by boredom: consuming food to escape awareness of the bored self. Frontiers in psychology. 2015;6:369.

103. Hulbert-Williams L, Hulbert-Williams NJ, Nicholls W, Williamson S, Poonia J, Hochard KD. Ultra-brief non-expert-delivered defusion and acceptance exercises for food cravings: A partial replication study. Journal of health psychology. 2019;24(12):1698-709.

104. van den Tol AJ, Coulthard H, Lang V, Wallis DJ. Are music listening strategies associated with reduced food consumption following negative mood inductions; a series of three exploratory experimental studies. Appetite. 2022;172:105947.

105. La Marra M, Caviglia G, Perrella R. Using smartphones when eating increases caloric intake in young people: an overview of the literature. Frontiers in Psychology. 2020;11:587886.

106. Eschenbeck H, Heim-Dreger U, Steinhilber A, Kohlmann C-W. Self-regulation of healthy nutrition: automatic and controlled processes. BMC psychology. 2016;4:1-8.

107. Tropper A. Effects of Self-Regulation and cognitive load on accessibility of temptation thoughts in restrained eaters: Hofstra University; 2016.

108. Ogden J, Oikonomou E, Alemany G. Distraction, restrained eating and disinhibition: an experimental study of food intake and the impact of ‘eating on the go’. Journal of health psychology. 2017;22(1):39-50.

109. Argo JJ, White K. When do consumers eat more? The role of appearance self-esteem and food packaging cues. Journal of Marketing. 2012;76(2):67-80.
